# A pore-forming toxin initiates ABI1 complex switching to promote bacterial cell-to-cell spread

**DOI:** 10.1038/s41467-026-71510-z

**Published:** 2026-04-13

**Authors:** He Sun, Arlon Wizzard, John H. Brumell, Darren E. Higgins

**Affiliations:** 1 Department of Microbiology, Blavatnik Institute, Harvard Medical School, Boston, MA USA; 2 Cell & Systems Biology Program and SickKids IBD Centre, Hospital for Sick Children, Toronto, ON Canada; 3 Department of Molecular Genetics and Institute of Medical Science, University of Toronto, Toronto, ON Canada

**Keywords:** Cellular microbiology, Pathogens, Actin, Bacterial pathogenesis, Bacterial toxins

## Abstract

*Listeria monocytogenes* (*Lm*), a prototypical intracellular bacterial pathogen, expresses ActA to initiate ARP2/3-mediated actin-based motility in the cytosol of host cells. Motile bacteria generate *Lm*-containing protrusions at the cell surface, facilitating direct cell-to-cell spread and dissemination within the host. Protrusion formation is an active, spatially regulated process, yet how *Lm* coordinates bacterial and host factors to orchestrate this process remains unclear. Here, we identify Abelson-interactor 1 (ABI1) as a host factor required for efficient *Lm* protrusion formation and cell-to-cell spread. Conditional knockout of *Abi1* in mice significantly reduces susceptibility to *Lm* infection, while deletion of *actA* abrogates the protective effect of *Abi1* knockout. During *Lm* infection, ABI1 is uncoupled from spectrin at the cell cortex and binds to EPS8 within protrusions. This ABI1 “complex switching” is initiated by the pore-forming toxin LLO, which perforates the host plasma membrane and triggers Ca^2+^ influx, leading to calpain-mediated cleavage of the spectrin cytoskeleton. Spectrin cleavage mobilizes ABI1, allowing ABI1 to bind EPS8 and activate EPS8’s actin capping activity to facilitate local actin recycling necessary for efficient protrusion elongation and cell-to-cell spread. These findings reveal an unrecognized host-pathogen interaction, in which a bacterial pore-forming toxin induces spatially confined cytoskeletal remodeling to promote cell-to-cell spread.

## Abstract (web summary)

A pore-forming toxin produced by *Listeria monocytogenes* induces changes in the host cell cytoskeleton that facilitate cell-to-cell spread of this intracellular bacterial pathogen. Here, Sun et al. show how the toxin modulates the activities and interactions of host protein complexes, enhancing local actin recycling necessary for cell-to-cell spread.

## Introduction

*Listeria monocytogenes* (*Lm*) is a foodborne intracellular bacterial pathogen that can cause life-threatening infections in newborns, immunocompromised individuals, pregnant people, and the elderly^1–3^. During infection of host cells, *Lm* exploits actin-based motility to propel itself within the host cytosol, spread between cells, and evade host immune responses^4^. This motility is driven by the *Lm* surface protein ActA^5,6^, which recruits host actin-binding proteins, including the ARP2/3 complex^7,8^ to assemble a polarized “comet tail” that propels *Lm* through the cytosol^9,10^. Continuous actin polymerization pushes *Lm* against the host cell plasma membrane (PM), leading to the formation of finger-like membrane extensions termed “*Lm*-containing protrusions”. These protrusions can be engulfed by adjacent cells to enable direct cell-to-cell spread, thus avoiding humoral immune responses^11^. Actin organization within protrusions is distinct from cytosolic comet tails^12,13^. Unlike the fully branched network generated exclusively by the ARP2/3 complex within comet tails, the actin filaments in *Lm*-containing protrusions display bundled filaments mediated by Diaphanous-related formins at the base of protrusions^14^ and branched filaments proximal to the bacteria where the ARP2/3 complex is localized^14^. Additional host factors—including EZRIN, MYO10, and the AIP1/CFL1/GMF/TWF2 actin depolymerization machinery—contribute to regulation of protrusion architecture and actin turnover^15–17^. While these findings define the structural organization of protrusions, how *Lm* actively manipulates host factor pathways to drive protrusion formation for efficient cell-to-cell spread remains unclear.

Pore-forming toxins (PFTs) are a widespread class of virulence factors expressed by many pathogenic bacteria and other organisms across all kingdoms of life^18^. Listeriolysin O (LLO), a cholesterol-dependent PFT secreted by *Lm*, is essential for vacuolar escape. Traditionally considered as a phagosome-specific lysin due to its optimal activity in acidic environments and limited cytosolic stability, LLO has been shown to damage the host PM at neutral pH^19^. Recent studies also highlighted a broader role for LLO in modulating host cell processes such as mitochondrial dynamics, SUMOylation, and histone modifications^20^. Notably, LLO also facilities cell-to-cell spread via efferocytosis by altering the PM lipid asymmetry and releasing *Lm*-containing protrusions^21^. Whether LLO contributes to other aspects of *Lm* dissemination, such as actin-based motility during protrusion formation, remains unknown.

A previous genome-wide RNAi screen identified Abelson-interactor 1 (ABI1) as a potential host factor required for *Lm* infection^22^. ABI1 is a key regulator of ABL kinase and plays an essential role in cytoskeletal reorganization and epidermal growth factor receptor (EGFR) signaling^23^. ABI1 interacts with multiple proteins within mammalian cells, generating distinct heteromeric complexes that support a range of cellular processes^24–29^. ABI1 is a core component of the WAVE regulatory complex (WRC), where it contributes to WAVE2-mediated ARP2/3 activation and lamellipodial actin assembly^28^. At the PM, ABI1 associates with spectrin, a core component of the spectrin-actin membrane cytoskeleton that supports PM architecture^30^, suggesting a structural role for ABI1 in maintaining cortical integrity and spatially restricting ABI1 availability^31^. ABI1 can also form a heteromeric complex with EPS8, contributing to the regulation of actin-rich structures^24,32^. The factors that regulate ABI1 recruitment to distinct heteromeric protein complexes, and how this recruitment partitions the functions of the resulting complexes to control specific cellular activities, remain unclear.

In this study, we demonstrate that conditional loss of *Abi1* confers significant protection against *Lm* infection in mice, underscoring its critical role in pathogenesis. Mechanistically, *Lm* is known to continuously secrete LLO after vacuole escape, resulting in PM damage^21^. We find that LLO-mediated PM perforation triggers Ca^2+^ (calcium) influx, which activates calpain, a calcium-dependent, cysteine protease. Calpain subsequently cleaves spectrin, which liberates ABI1 from its cortical PM, spectrin-anchored pool, thus enabling ABI1 recruitment to *Lm*-containing protrusions. ABI1 then forms a new complex with EPS8 within protrusions and switches EPS8 activity from F-actin bundling to barbed-end capping, thereby enhancing local actin recycling and facilitating protrusion extension. Our findings uncover a pathogen-driven, PFT-triggered ABI1 complex switching that allows rapid actin remodeling for bacterial cell-to-cell spread.

## Results

### *Abi1* knockout reduces *Lm* virulence during mouse infection

**Fig. 1:**
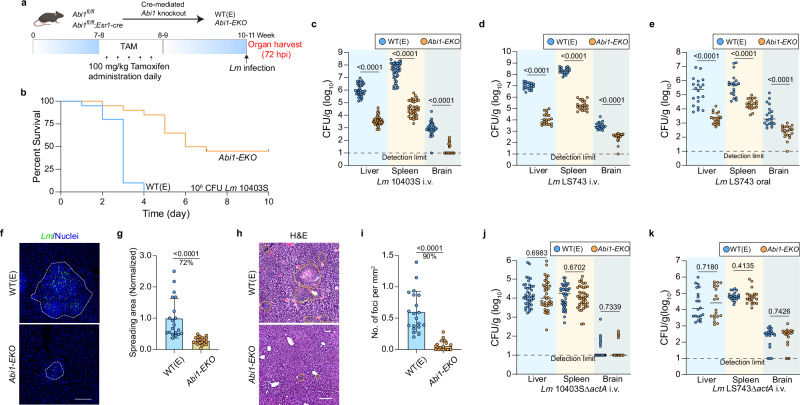
*Abi1* knockout reduces *Lm* virulence and efficient cell-to-cell spread during mouse infection. **a** Seven to eight-week-old *Abi1*^*fl/fl*^ and *Abi1*^*fl/fl*^; *Esr1-cre* mice were administered 100 mg/kg tamoxifen intraperitoneally for five consecutive days. *Abi1*^*fl/fl*^ or *Abi1*^*fl/fl*^; *Esr1-cre* mice that were treated with tamoxifen are referred to as WT(E) and *Abi1-EKO*, respectively. Fourteen days post-induction, mice were infected with *Lm* as indicated. Target organs were harvested 72 hpi for analysis of bacterial burdens. **b** Kaplan-Meier curve showing the percent survival of mice infected with 1 × 10^6^ CFU *Lm* 10403S via intravenous injection. *N* = 20 mice per each group. **c** WT(E) and *Abi1-EKO* mice were infected with 5×10^4^ CFU *Lm* 10403S via intravenous injection. At 72 hpi, bacterial burden in organs was determined. *N* = 38 for WT(E) and 40 for *Abi1-EKO* mice. **d** WT(E) and *Abi1-EKO* mice were infected with 1×10^4^ CFU *Lm* LS743 via intravenous injection. At 72 hpi, bacterial burden in organs was determined. *N* = 20 mice per each group. **e** WT(E) and *Abi1-EKO* mice were infected with 2×10^9^ CFU *Lm* LS743 via oral gavage. At 72 hpi, bacterial burden in organs was determined. *N* = 20 mice per each group. **f** Representative images of infection foci in the liver of WT(E) and *Abi1-EKO* mice at 72 hpi. Tissues were stained with *Lm* antibody and Hoechst 33342 (cell nuclei). Dotted lines delineate edge of the infection foci. Scale bar=100 μm. **g** Quantification of the spreading area of infection foci. The mean spreading area of WT(E) infection foci was calculated and used as the reference value. *N* = 20 foci quantified from liver sections derived from five mice. Data are presented as mean ± SD with all data points shown and percent reduction in spreading area indicated. **h** Representative images of infection foci in the liver of WT(E) and *Abi1-EKO* mice at 72 hpi. Tissues were stained with H&E. Dotted lines delineate edge of the infection foci. Scale bar=200 μm. **i** Quantification of the number of infection foci. *N* = 20 images of liver sections derived from five mice. Data are presented as mean ± SD with all data points shown and percent reduction in the number of infection foci per square millimeter indicated. **j** WT(E) and *Abi1-EKO* mice were infected with 1 × 10^7^ CFU *Lm* 10403SΔ*actA* via intravenous injection. At 72 hpi, bacterial burden in organs was determined. Data are shown as medians. *N* = 40 mice. **k** WT(E) and *Abi1-EKO* mice were infected with 1 × 10^7^ CFU *Lm* LS743Δ*actA* via intravenous injection. At 72 hpi, bacterial burden in organs was determined. Data are shown as medians. *N* = 20 mice. Horizontal bars indicate median values with data from individual mice plotted (**c**–**e**,**j**,**k**). The dashed line indicates a limit of detection of 10 CFU (**c**–**e**,**j**,**k**). The Mann-Whitney *U* test (two-sided) was used to assess statistical significance (**c**–**e**,**j**,**k**). The two-tailed unpaired Welch’s *t*-test (unequal variance) was used to assess statistical significance (**g**,**i**). Exact *P* values are shown in the figures. The diagram in (**a)** was created in BioRender. Sun, H. (2026) https://BioRender.com/wwbb7ml. Source data are provided as a Source Data file.

*Abi1* knockout mice are embryonic lethal due to ABI1’s crucial roles in cardiovascular and placental development^29,33^. Thus, to investigate the role of ABI1 during *Lm* infection in vivo, we generated conditional *Abi1* knockout (KO) mice (*Abi1*^*fl/fl*^; *Esr1-cre*) by crossing *Abi1*^*fl/fl*^ mice with *Esr1-cre* mice^29,34^. Seven to eight-week-old *Abi1*^*fl/fl*^ and *Abi1*^*fl/fl*^; *Esr1-cre* mice were administered tamoxifen (100 mg/kg) intraperitoneally for five consecutive days (Fig. 1a). We refer to tamoxifen-treated *Abi1*^*fl/fl*^ or tamoxifen-treated *Abi1*^*fl/fl*^; *Esr1-cre* mice as WT(E) or *Abi1-EKO*, respectively. Fourteen days post-induction, we confirmed ABI1 depletion in the liver, spleen, and brain (Supplementary Fig. 1a), and subsequently used these mice for *Lm* infection studies (Fig. 1a).

To assess the requirement of ABI1 for *Lm* virulence in mice, WT(E) and *Abi1-EKO* mice were injected intravenously with 10^6^ CFU *Lm* 10403S (a widely used *Lm* laboratory strain)^35^ and monitored for 10-day survival. *Abi1-EKO* mice were protected against infection, with an increased percentage of mice survival (Fig. 1b). Next, WT(E) and *Abi1-EKO* mice were intravenously infected with 5 × 10^4^ CFU of *Lm* 10403S, and bacterial burdens in the spleen, liver and brain of each mouse were determined 72 hours post-infection (hpi). *Abi1-EKO* mice displayed significantly reduced bacteria harvested from the liver (2.5 logs reduction), spleen (2.9 logs reduction), and brain (1.8 logs reduction) compared to WT(E) animals (Fig. 1c). Control experiments ruled out potential tamoxifen side effects, as bacterial burdens were comparable in tamoxifen or corn oil (carrier control)-treated WT mice. *Abi1-EKO* mice consistently showed 2-3 log CFU reductions in livers and spleens, and 1-2 log reductions in the brain (Supplementary Fig. 1b–d). Moreover, an increased deduced LD_50_ for *Abi1-EKO* mice (~ 8 × 10^5^ to 10^6^ CFU *Lm* 10403S) compared to WT(E) mice (~ 5 × 10^4^ CFU *Lm* 10403S) indicated a critical role of ABI1 in *Lm* virulence in vivo (Fig. 1b and Supplementary Fig. 1e, f). We also evaluated infection with *Lm* LS743, a clinical strain with characterized increased virulence that was isolated from a sustained 2011 U.S. cantaloupe outbreak^35^. Following tail vein injection of 10^4^ CFU *Lm* LS743, *Abi1-EKO* mice exhibited similar reductions in bacterial burdens in the liver (2.7 logs reduction), spleen (2.9 logs reduction), and brain (1 log reduction) (Fig. 1d). Additionally, in an orogastric infection model (the natural route of *Lm* infection in humans), WT(E) and *Abi1-EKO* mice were orally inoculated with 2 × 10^9^ CFU *Lm* LS743. *Abi1-EKO* mice again demonstrated protection against *Lm* LS743 showing a 2.5 logs CFU reduction in the liver, 1.9 logs CFU reductions in the spleen, and 1.6 logs CFU reductions in the brain at 72 hpi (Fig. 1e).

To rule out the potential for mouse genetic background effects, we generated a different conditional *Abi1* KO mouse, *Abi1*^*fl/fl*^; *Mx1-cre*, by crossing *Abi1*^*fl/fl*^ mice to *Mx1-cre* mice^36^. *Abi1* KO was induced in seven to eight-week-old *Abi1*^*fl/fl*^ and *Abi1*^*fl/fl*^; *Mx1-cre* mice by intraperitoneal Poly(I:C) injections (10 mg/kg) every other day for four doses. Poly(I:C)-treated *Abi1*^*fl/fl*^ or Poly(I:C)-treated *Abi1*^*fl/fl*^; *Mx1-cre* mice were referred to as WT(M) or *Abi1-MKO*, respectively (Supplementary Fig. 1g). Fourteen days post-induction, these mice were used for *Lm* infection experiments. *Abi1-MKO* mice showed increased survival compared to WT(M) mice after infection with 10^6^ *Lm* 10403S (Supplementary Fig. 1h). Furthermore, *Abi1-MKO* mice exhibited markedly reduced bacterial burdens in the liver (2.9 logs reduction), spleen (2.6 logs reduction), and brain (2 logs reduction) following infection with 5 × 10^4^ CFU of *Lm* 10403S (Supplementary Fig. 1i). Similarly, both tail vein and oral gavage infections with *Lm* LS743 in *Abi1-MKO* mice resulted in reduced bacterial organ burdens compared to infections of WT(M) mice (Supplementary Fig. 1j, k).

Consistent with a previous report^37^, the efficiency of ABI1 KO in *Abi1-MKO* mice was confirmed in the liver and spleen, with levels of ABI1 in the brain remaining unchanged compared to WT(M) mice (Supplementary Fig. 1l). This suggests that the late-stage brain infection may depend on *Lm* propagation in other organs. Alternatively, ABI1 may be specifically depleted in brain endothelial cells^38^. Since traversal of brain endothelial cells is a major obstacle for *Lm* crossing the blood-brain barrier to infect the brain, this may result in reduced bacterial burdens in the brain (Supplementary Fig. 1i–k) despite no significant reduction in ABI1 protein levels in the brains of *Abi1-MKO* mice (Supplementary Fig. 1l)^38,39^. Future studies employing brain endothelial cell-specific *Abi1* KO models will be valuable for dissecting the contribution of ABI1 to *Lm* translocation across the blood-brain barrier. Furthermore, to rule out Poly(I:C)-related effects, we also compared bacterial burdens in *Abi1*^*fl/fl*^ and *Abi1*^*fl/fl*^; *Mx1-cre* mice administered either Poly(I:C) or physiological buffer (PB, 0.9% NaCl, used as the Poly(I:C) solvent). No significant differences in bacterial burdens were found in the liver, spleen or brain of *Abi1*^*fl/fl*^ mice treated with Poly(I:C) or PB and *Abi1*^*fl/f*l^; *Mx1-cre* mice treated with PB (Supplementary Fig. 1m–o). Notably, an increased deduced LD_50_ for *Abi1-MKO* mice (1 × 10^6^ to 3 × 10^6^ CFU *Lm* 10403S) compared to WT(M) mice (~ 5 × 10^4^ CFU *Lm* 10403S) was also observed, indicating similar protection against *Lm* infection when compared to *Abi1-EKO* mice (Supplementary Fig. 1e, f, p, q). Collectively, our results indicate that *Abi1* KO leads to reduced *Lm* virulence within two different in vivo mouse models, using different *Lm* strains and infection routes.

### ABI1 contributes to efficient *Lm* cell-to-cell spread in vivo

To understand the cause of reduced *Lm* virulence in *Abi1* KO mice, we examined tissue-level infection outcomes. Liver sections from *Abi1-EKO* and *Abi1-MKO* mice exhibited markedly smaller infection foci (72% reduction for *Abi1-EKO*, 59% reduction for *Abi1-MKO*) and fewer infection foci per square millimeter of liver tissue compared to WT mice (90% reduction for *Abi1-EKO*, 87% reduction for *Abi1-MKO*) (Fig. 1f–i and Supplementary Fig. 2a–d). This suggests that ABI1 is important for cell-to-cell spread in vivo. Supporting this hypothesis, infection with *Lm* strains deficient in actin-based motility (10403SΔ*actA* or LS743Δ*actA*) eliminated the difference in bacterial burdens between WT and *Abi1*-deficient mice (Fig. 1j, k and Supplementary Fig. 2e, f). Taken together, these results suggest an important role of ABI1 in promoting *Lm* cell-to-cell spread in vivo.

### *ABI1* deletion impairs *Lm* cell-to-cell spread in vitro

**Fig. 2:**
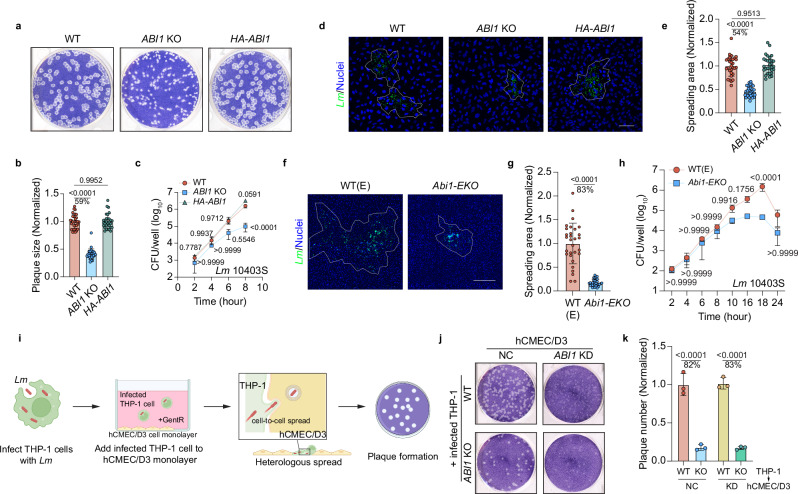
Deletion of *ABI1* impairs *Lm* cell-to-cell spread. **a** WT, *ABI1 KO*, and *HA-ABI1* HeLa cells were infected with *Lm* 10403S. After 1 hour, infected monolayers were washed and overlaid with medium containing gentamicin. At 72 hpi, the monolayers were fixed and stained with crystal violet to visualize plaque formation. **b** Plaque size measurements from (**a**). Data are presented as the mean ± SD plaque area size normalized to WT HeLa cells with all data points shown and the percent reduction in plaque size indicated. *N* = 30 plaques. **c** WT, *ABI1 KO*, and *HA-ABI1* HeLa cells were infected with *Lm* 10403S. After 1 hour, infected monolayers were washed, and gentamicin-containing medium was added. HeLa cells were lysed at 2-hour intervals post-infection and intracellular bacteria were quantified by plating serial dilutions on agar plates. Data are presented as the mean ± SD of a representative experiment performed in triplicate and repeated three times with similar results. Statistical analysis was performed as described and differences compared to the WT group are indicated. **d**,**e** Representative images (**d**) and quantification (**e**) from an infection focus assay assessing *Lm* 10403S cell-to-cell spread in WT, *ABI1 KO*, and *HA-ABI1* HeLa cells. Dotted lines delineate the edges of infection foci. Scale bar=100 μm. Data are presented as mean ± SD with all data points shown and percent reduction in spreading area indicated. *N* = 30 foci. **f**,**g** Representative images (**f**) and quantification (**g**) from an infection focus assay to measure *Lm* 10403S cell-to-cell spread in BMDM isolated from WT(E) and *Abi1-EKO* mice. Dotted lines delineate the edges of infection foci. Scale bar=300 μm. Data are presented as mean ± SD with all data points shown and percent reduction in spreading area indicated. *N* = 30 foci. **h** BMDM isolated from WT(E) and *Abi1-EKO* mice were infected with *Lm* 10403S and intracellular growth analyzed as described in (**c**). Data are presented as mean ± SD of a representative experiment performed in triplicate and repeated three times with similar results. **i** Schematic of the heterologous plaque formation assay. **j** WT, and *ABI1 KO* THP-1 cells were infected with *Lm* 10403S. After 1 h, infected THP-1 cells were washed, then overlaid with gentamicin-containing medium for an additional hour. The infected THP-1 cells were detached and transferred to a confluent monolayer of either NC (scrambled shRNA control) or *ABI1 KD* hCMEC/D3 cells. At 72 hpi, monolayers were fixed and stained with crystal violet to visualize plaques. **k** Quantification of plaque numbers from (**j**). *N* = 3 biological replicates with total plaques counted per biological replicate ranging from 174 to 228 for WT THP-1, and 29 to 43 for *ABI1* KO THP-1 cell conditions. Data are presented as mean ± SD and percent reduction in plaque number indicated. One-way ANOVA (equal variance) analysis followed by Tukey’s post-hoc test was performed to assess statistical significance (**b**). Repeated-Measures two-way ANOVA model analysis followed by Šidák’s post-hoc test was performed to assess statistical significance (**c**,**h**). Welch’s one-way ANOVA (unequal variance) analysis followed by Dunnett’s T3 post-hoc test was performed to assess statistical significance (**e**). The two-tailed unpaired Welch’s *t*-test (unequal variance) was used to assess statistical significance (**g**). Two-way ANOVA (equal variance) analysis followed by Tukey’s post-hoc test was performed to assess statistical significance (**k**). Exact *P* values are shown in the figures. The diagram in (**i**) was created in BioRender. Sun, H. (2026) https://BioRender.com/wwbb7ml. Source data are provided as a Source Data file.

To investigate the mechanism of ABI1 function during *Lm* cell-to-cell spread, we used CRISPR-Cas9-mediated gene editing to delete *ABI1* in HeLa cells (Supplementary Fig. 3a). An ABI1 complementation cell line (*HA-ABI1*) was also generated by inserting an N-terminal HA-tagged ABI1 expression cassette into *ABI1* KO HeLa cells (Supplementary Fig. 3b, c)^40^. We first assessed infection using a plaque formation assay, which assesses efficiency of bacterial uptake (number of plaques formed) and cell-to-cell spread (size of plaques generated). A previous study reported reduced invasion of *Lm* in a HeLa cell model with siRNA-mediated *ABI1* knockdown^41^. However, our findings revealed no significant difference in plaque numbers (Supplementary Fig. 3d), suggesting that ABI1 may not be directly involved in bacterial uptake in HeLa cells. However, we found a significant ~59% reduction in plaque area size in *ABI1* KO HeLa cells compared to wild-type (WT) HeLa cells, which was restored with *HA-ABI1* complementation (Fig. 2a, b). This suggested a potential role for ABI1 in cell-to-cell spread in HeLa cells.

Intracellular growth of *Lm* was not affected in *ABI1* KO HeLa cells up to 6 hpi (Fig. 2c), indicating that ABI1 does not play a role in *Lm* vacuole escape or early intracellular replication. However, we observed significantly less intracellular bacteria in *ABI1* KO HeLa cells at 8 hpi, suggesting a cell-to-cell spread defect. In support of this notion, the late-stage replication defect observed in *ABI1* KO cells was not observed following infection with Δ*actA* bacteria (10403SΔ*actA*) (Supplementary Fig. 4a). We also examined infection foci in monolayers of WT, *ABI1* KO, and *HA-ABI1* cells infected with GFP-expressing *Lm* 10403S (GFP-*Lm*). Confocal fluorescence microscopy at 6 hpi revealed a 54% reduction in focus area in *ABI1* KO cells compared to WT and *HA-ABI1* cells (Fig. 2d, e), indicating lower cell-to-cell spread between *ABI1* KO cells. These findings support that ABI1 is required for efficient cell-to-cell spread in HeLa cells.

To further investigate ABI1 function in cell types relevant to *Lm* infection, we generated *ABI1* KO Caco2 (intestinal epithelial), HepG2 (hepatocyte), JEG-3 (feto-placental) and THP-1 (macrophage) human-derived cell lines. We were unable to achieve *ABI1* KO in hCMEC/D3 cells (brain endothelial), thus *ABI1* knockdown (KD) hCMEC/D3 cells were generated using lentivirus-mediated shRNA interference. ABI1 expression was undetectable in the KO cell lines and markedly reduced in the hCMEC/D3 KD cell line (Supplementary Fig. 3a). Consistent with plaque formation assay results in HeLa *ABI1* KO cells, we observed a 45% reduction in plaque size in Caco2 *ABI1* KO cells, a 21% reduction in HepG2 *ABI1* KO cells, and a 27% reduction in hCMEC/D3 *ABI1* KD cells (Supplementary Fig. 3e, f, j, k, t, u). Notably, JEG-3 *ABI1* KO cells showed a significant 69% reduction in plaque size (Supplementary Fig. 3o, p), suggesting a potential role of ABI1 in *Lm* crossing the maternal-fetal barrier. Interestingly, a 41% reduction in plaque numbers was observed in *ABI1* KO HepG2 cells but not in other cell lines tested, suggesting a potential role for ABI1 in bacterial uptake in specific cell types (Supplementary Fig. 3f, k, p, u). This observation also provides a plausible explanation for the reduced number of liver infection foci observed in *Abi1* KO mice (Fig. 1h, i and Supplementary Fig. 2c, d). Intracellular growth assays in Caco2, HepG2, JEG-3 and hCMEC/D3 cells indicated that *ABI1* KO/KD did not affect intracellular replication rates until 6–8 hpi (Supplementary Fig. 3g, l, q, v) suggesting that ABI1 does not play a role in *Lm* vacuole escape, and early intracellular replication in these cell types, but is critical for late-stage cell-to-cell spread. Similarly, the replication defects were not observed when using 10403SΔ*actA*, highlighting the role of ABI1 specifically in cell-to-cell spread (Supplementary Fig. 4b, c, d, e). Furthermore, fluorescence microscopy of infection foci revealed reductions in spread in the absence of ABI1: 53% in Caco2, 60% in HepG2, 58% in JEG-3 and a 52% reduction in hCMEC/D3 cells with a reduced level of ABI1 (Supplementary Fig. 3h, i, m, n, r, s, w, x).

Infection of macrophages is characterized by slower bacterial growth and less efficient cell-to-cell spread compared with epithelial cells^42^. To explore the role of ABI1 during cell-to-cell spread in professional phagocytes, we examined *Lm* infections over a 24-hour period in bone marrow-derived macrophages (BMDM) from *Abi1-EKO* and *Abi1-MKO* mice (Supplementary Fig. 1a, l). *Lm* spread was reduced by 83% in *Abi1-EKO* BMDM and by 79% in *Abi1-MKO* BMDM at 18 hpi (Fig. 2f, g and Supplementary Fig. 4g, h). Notably, intracellular growth of *Lm* remained unaffected until 16-18 hpi in BMDM from both *Abi1-EKO* and *Abi1-MKO* mice (Fig. 2h and Supplementary Fig. 4i). Moreover, the late-stage reduction in intracellular growth observed in *Abi1* KO BMDM was absent during infection with the non-spreading *Lm* 10403SΔ*actA* mutant (Supplementary Fig. 4f, j). Collectively, these results suggest that cell-to-cell spread was specifically impaired in *Abi1* KO BMDM.

The ability of *Lm* to cross the blood-brain barrier (BBB) and invade the central nervous system (CNS) can result in life-threatening infections such as meningitis and encephalitis^43,44^. One critical route of brain invasion involves the traversal of *Lm* from infected phagocytes across the BBB via cell-to-cell spread into endothelial cells^45^. To model phagocyte-to-endothelial cell spread, THP-1 WT or *ABI1* KO cells were infected with *Lm*, washed after one hour, and incubated in gentamicin-containing medium for an additional hour. No invasion defects were detected in THP-1 *ABI1* KO cells (Supplementary Fig. 4k). The infected THP-1 WT or *ABI1* KO cells were then resuspended and added to a confluent monolayer of hCMEC/D3 cells, either NC (negative control) or *ABI1* KD cells (Fig. 2i). Plaque formation served as an indicator of successful heterologous spread from THP-1 cells to hCMEC/D3 cells with subsequent cell-to-cell spreading within the hCMEC/D3 cell monolayer. *ABI1* KO in THP-1 cells markedly reduced plaque numbers, suggesting a significant defect in heterologous spreading (Fig. 2j, k). Consistent with prior findings, a reduction in plaque size was observed in hCMEC/D3 *ABI1* KD cells (Supplementary Fig. 4l). These findings collectively highlight that ABI1 is required for efficient *Lm* cell-to-cell spread in different cell types.

### ABI1 localizes to *Lm*-containing protrusions during *Lm* cell-to-cell spread

**Fig. 3:**
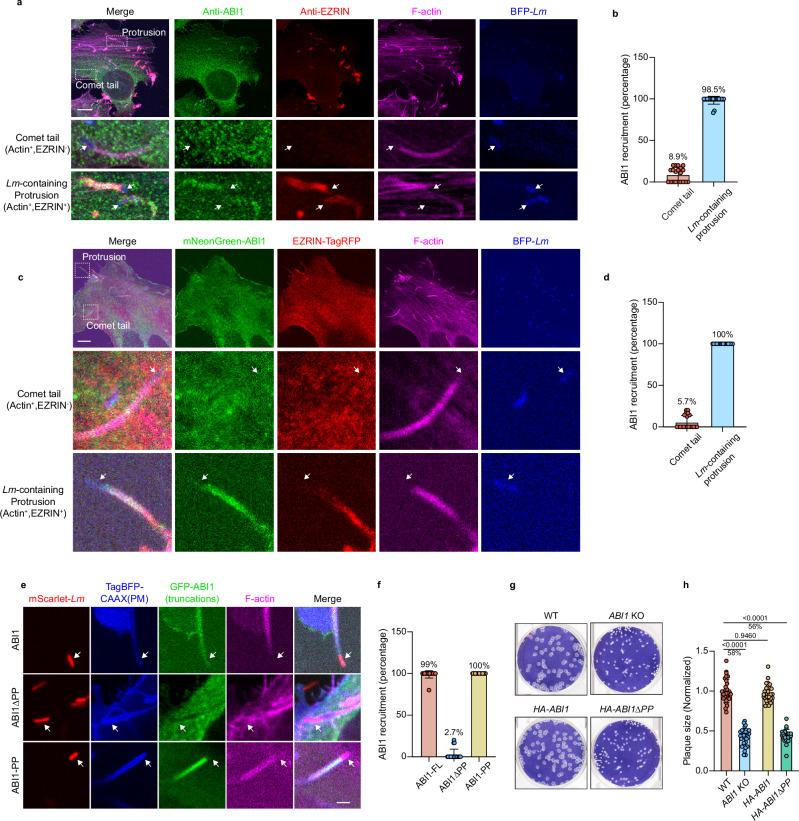
ABI1 localizes to *Lm*-containing protrusions during *Lm* cell-to-cell spread. **a** WT HeLa cells were infected with *Lm* 10403S expressing TagBFP (BFP-*Lm*) for 6 hours, then immunostained with Alexa Fluor^TM^ 647 phalloidin (to visualize F-actin) and antibodies to EZRIN and ABI1. Comet tail (middle panels) and protrusion (lower panels) structures are shown. Arrowheads indicate bacteria associated with either protrusions or comet tails. Scale bar=10 μm. **b** Recruitment of ABI1 to comet tails (Actin^+^, EZRIN^-^) and *Lm*-containing protrusions (Actin^+^, EZRIN^+^) from (**a**). ABI1 recruitment was calculated as the number of ABI1-positive comet tails or *Lm*-containing protrusions divided by the total number of quantified comet tails or *Lm*-containing protrusions within a single infected cell (*N* = 20 cells). Data are presented as mean ± SD with all data points shown and the percent ABI1 recruitment indicated. **c** HeLa cells expressing mNeonGreen-ABI1 (green channel) and EZRIN-TagRFP (red channel) were infected with *Lm* 10403S expressing TagBFP (BFP-*Lm*) (blue channel). F-actin was visualized using SiR-actin stain (magenta channel). Images were captured at 5-7 hpi. Arrowheads mark *Lm* associated with a comet tail (middle panels) or protrusion (lower panels). Scale bar=10 μm. **d** Recruitment of mNeonGreen-ABI1 to comet tails (Actin^+^, EZRIN^-^) and *Lm*-containing protrusions (Actin^+^, EZRIN^+^) from (**c**). ABI1 recruitment was calculated as the number of mNeonGreen-ABI1-positive comet tails or *Lm*-containing protrusions divided by the total number of quantified comet tails or *Lm*-containing protrusions within a single infected cell (*N* = 20 cells). Data are presented as mean ± SD with all data points shown and the percent ABI1 recruitment indicated. **e** *ABI1* KO HeLa cells were transfected with constructs expressing GFP-ABI1, GFP-ABI1ΔPP, or GFP-ABI1-PP along with TagBFP-CAAX (to visualize the plasma membrane (PM)), and LifeAct-iRFP670 (to visualize F-actin), then infected with *Lm* 10403S expressing mScarlet (mScarlet-*Lm*). Images were captured at 5-7 hpi. Arrowheads mark *Lm* associated with a protrusion. Scale bar=2 μm. **f** Recruitment of GFP-ABI1, GFP-ABI1ΔPP, and GFP-ABI1-PP to *Lm*-containing protrusions (Actin^+^, TagBFP-CAAX^+^) from (**e**). ABI1 recruitment was calculated as the number of GFP-positive *Lm*-containing protrusions divided by the total number of quantified *Lm*-containing protrusions within a single infected cell (*N* = 20 cells). Data are presented as mean ± SD with all data points shown and the percent ABI1 recruitment indicated. **g** WT, *ABI1* KO, *HA-ABI1* and *HA-ABI1*Δ*PP* HeLa cells were infected with *Lm* 10403S and plaque formation at 72 hpi visualized as previously described. **h** Plaque size measurement from (**g**). *N* = 30 plaques. Data are presented as mean ± SD with all data points shown and percent reduction in plaque size indicated. Welch’s one-way ANOVA (unequal variance) analysis followed by Dunnett’s T3 post-hoc test was performed to assess statistical significance (**h**). Exact *P* values are shown in the figures. Source data are provided as a Source Data file.

We next examined the subcellular localization of ABI1 during *Lm* infection. HeLa cells infected with *Lm* 10403S expressing TagBFP (BFP-*Lm*) were immunostained with an ABI1-specific antibody. EZRIN was used as a marker for *Lm*-containing protrusions^15^ to help distinguish *Lm* comet tails localized to protrusion structures. High-resolution immunofluorescence microscopy revealed that ABI1 colocalizes specifically with *Lm*-containing protrusions marked by EZRIN and actin (EZRIN^+^, Actin^+^), while ABI1 was absent from actin comet tails in the cytosol (EZRIN^-^, Actin^+^) (Fig. 3a, b).

To rule out potential non-specific antibody staining and limitations in epitope accessibility within dense actin-rich protrusions, as well as to further explore the spatiotemporal dynamics of ABI1 recruitment, we used CRISPR-Cas9-mediated homology-directed repair to tag ABI1 endogenously with mNeonGreen in HeLa cells (Supplementary Fig. 5a)^46^. Consistent with previous reports, ABI1 localizes to lamellipodia, filopodia, and concentrates at cell-cell contact sites (Supplementary Fig. 5b)^47,48^. Additionally, EZRIN was tagged with TagRFP to label protrusions using a modified CRISPaint technique^49^. Spinning disk confocal microscopy in live cells revealed that, consistent with immunofluorescence (IF) results, mNeonGreen-ABI1 is recruited specifically to *Lm*-containing protrusions during their initiation and elongation, but not to cytosolic actin comet tails (Fig. 3c, d and Supplementary Movie 1). Together, these IF and live-cell imaging results suggest that ABI1 is recruited to *Lm*-containing protrusions.

### The Polyproline Rich region of ABI1 is required for localization to *Lm*-containing protrusions

ABI1 interacts with diverse binding partners through multiple domains (Supplementary Fig. 5c)^23^. To determine the region responsible for ABI1’s localization to *Lm*-containing protrusions, we expressed a GFP-ABI1 full-length and various truncated ABI1 constructs in *ABI1* KO HeLa cells (Supplementary Fig. 5d). TagBFP-CAAX (CAAX sequence derived from KRAS4B) was co-transfected to label the PM, and LifeAct-iRFP670 was used to visualize F-actin^50,51^. Cells were infected with *Lm* 10403S expressing mScarlet (mScarlet-*Lm*) to track bacterial localization. Notably, all ABI1 constructs containing the Polyproline Rich (PP) region co-localized with *Lm*-containing protrusions (Supplementary Fig. 6a–m), while deletion of the PP region abolished this localization (Fig. 3e, f). To further test the role of the PP region, we generated a complementation cell line expressing an HA-tagged ABI1 lacking the PP region (*HA-ABI1*Δ*PP*) in HeLa *ABI1* KO cells (Supplementary Fig. 3b, c). Expression of ABI1 lacking the PP region failed to complement the reduced plaque size observed in the *ABI1* KO HeLa cells (Fig. 3g, h). These findings indicate that the PP region of ABI1 is crucial for its recruitment to *Lm*-containing protrusions and is necessary for efficient cell-to-cell spread.

### *ABI1* KO impairs protrusion dynamics

**Fig. 4:**
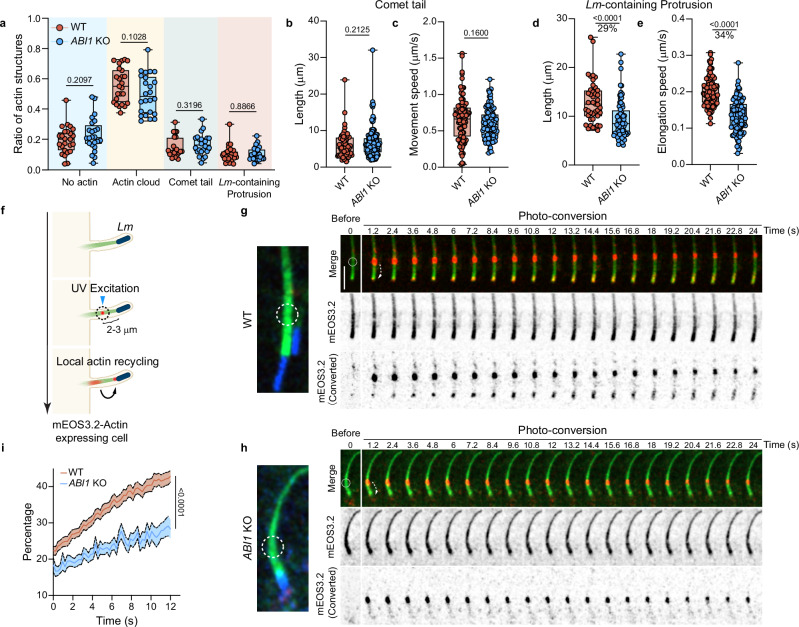
*ABI1* KO impairs protrusion dynamics. **a** Ratio of *Lm* associated with different actin structures (no actin, actin cloud, comet tail, or *Lm*-containing protrusion) in WT or *ABI1* KO HeLa cells at 6 hpi. *N* = 25 (WT), 24 (*ABI1* KO) infected cells. **b** Length measurements of actin comet tails associated with *Lm* in WT or *ABI* KO HeLa cells at 6 hpi. *N* = 62 (WT), 118 (*ABI1* KO) comet tails. **c** Movement speed of *Lm* associated with actin comet tails in WT or *ABI* KO HeLa cells at 6 hpi. *N* = 100 *Lm* associated with actin comet tails. **d** Length measurements of *Lm-*containing protrusions in WT or *ABI1* KO HeLa cells at 6 hpi. *N* = 42 (WT), 72 (*ABI1* KO) *Lm-*containing protrusions. Percent reduction in protrusion length is indicated. **e** Elongation speed of *Lm-*containing protrusions in WT or *ABI1* KO HeLa cells at 6 hpi. *N* = 100 *Lm-*containing protrusions. Percent reduction in elongation speed is indicated. **f** Schematic of the local actin recycling tracking experiment. **g**,**h** Representative images of mEOS3.2-Actin (green) and photo-converted mEOS3.2-Actin (red) dynamics within 24 s after photo-conversion in *Lm*-containing protrusions in WT (**g**) and *ABI1* KO (**h**) HeLa cells. Dashed arrows indicate the direction of actin flow. Scale bar=5 μm. **i** Local actin recycling quantification analysis. The quantification of photo-converted mEOS3.2-Actin was initiated from the first frame acquired immediately after photo-conversion (time interval = 0.24 s). The percentage of photo-converted mEOS3.2-Actin that traveled to the bacterial pole of the total photo-converted mEOS3.2-Actin within 12 s is shown. *N* = 20 photo-conversion and local actin recycling tracking experiments. Data are presented as mean ± SEM, plotted as a function of time. The shaded area represents the SEM. Box and Whisker plots representing the 25 to 75th percentiles (box) with the min and max values (whiskers) for all data points for each measurement is indicated (**a**–**e**). The line in the middle of the box is plotted at the median. The two-tailed unpaired student’s *t*-test (equal variance) was used to assess statistical significance (**a**,**b**,**d**,**e**). The two-tailed unpaired Welch’s *t*-test (unequal variance) was used to assess statistical significance (**c**). Mixed-effects model analysis (two-sided) was performed to assess statistical significance (**i**). Exact *P* values are shown in the figures. The diagram in (**f)** was created in BioRender. Sun, H. (2026) https://BioRender.com/wwbb7ml. Source data are provided as a Source Data file.

To investigate the specific role of ABI1 in actin-based motility, we compared actin structure dynamics between WT and *ABI1* KO HeLa cells. Cells were infected with *Lm* for 1 hour, followed by an additional 5-hour incubation in gentamicin containing media before fixation. F-actin was stained with Texas red™-X Phalloidin, and we quantified different *Lm*-associated actin structures (actin clouds, actin comet tails, and *Lm*-containing protrusions). The comparable ratios of actin clouds and comet tails between WT and *ABI1* KO cells, together with the specific localization of ABI1 to *Lm*-containing protrusions, indicate that ABI1 is apparently not required for actin cloud or comet tail formation (Fig. 4a). Moreover, we observed no significant differences in the frequency of *Lm*-containing protrusions between WT and *ABI1* KO cells, suggesting that ABI1 is likely dispensable for the initial formation of *Lm*-containing protrusions (Fig. 4a). Live-cell imaging showed that comet tail length and the movement speed of *Lm* with comet tails were comparable between WT and *ABI1* KO cells (Fig. 4b, c). However, *Lm*-containing protrusions in *ABI1* KO cells were approximately 29% shorter and extended at a 34% slower rate than in WT cells (Fig. 4d, e). Combined with our previous findings of ABI1 localization to protrusions during infection, these results suggest that ABI1 contributes to protrusion elongation.

Efficient elongation of *Lm*-containing protrusions relies on the local recycling of actin monomers and the ARP2/3 complex^17^. Actin recycling is supported by the actin depolymerization process, which disassembles the distal actin network to fuel ARP2/3-mediated assembly at the bacterial pole within the protrusion, generating forces against the PM^17^. We hypothesized that ABI1 might play a role in this localized actin recycling process. To test this, we engineered HeLa WT and *ABI1* KO cell lines to express a mEOS3.2-tagged actin cassette at the AAVS1 inert locus ensuring consistent expression. The mEOS3.2 is a photo-convertible fluorescent protein that shifts from green to red upon near-UV exposure, allowing real-time monitoring of actin redistribution^52^. Using this mEOS3.2-Actin reporter system, we analyzed the redistribution of actin and the lateral mobility of actin flow within protrusions. A circular region of interest (ROI) within ~2-3 μm of the proximal bacterial pole (i.e. the bacterial pole associated with the comet tail) was photo-converted and the redistribution of the converted mEOS3.2 (red) was tracked (Fig. 4f). As expected, disassembly of the red-labeled actin in the distal actin network recycled back toward the bacterial pole fueling the assembly of the proximal network and driving protrusion elongation (Fig. 4f–h and Supplementary Movie 2). To quantify local actin recycling efficiency within *Lm*-containing protrusions, we applied Gaussian blur filters for enhanced image contrast and performed single-particle tracking using the Trackmate plugin (Fiji). The proportion of recycled actin (proximal red signal) was calculated (Supplementary Fig. 6n). Strikingly, *ABI1* KO cells showed a substantial reduction in local actin recycling efficiency, suggesting potential impaired actin polymerization and force generation (Fig. 4g–i and Supplementary Movie 2). These findings indicated that ABI1 is required for efficient local actin recycling within *Lm*-containing protrusions to promote their elongation.

### ABI1 interacts with EPS8 and promotes EPS8 actin capping activity in *Lm*-containing protrusions

**Fig. 5:**
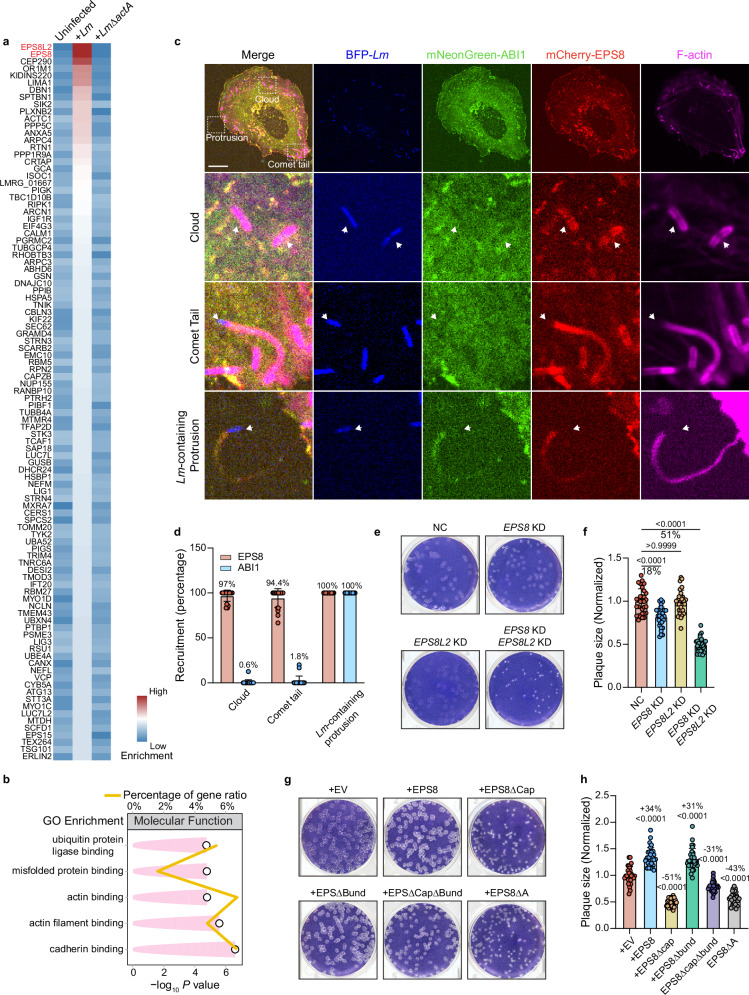
ABI1 interacts with EPS8 and switches EPS8 activities in *Lm*-containing protrusions. **a** ABI1 interactome data obtained by Quantitative Immunoprecipitation Mass Spectrometry (Quant IP-MS) analysis. Legend denotes relative protein abundance among uninfected, *Lm* 10403S infected and *Lm* 10403SΔ*actA* infected groups. Three biological replicates were performed. The top 100 proteins enriched in the *Lm* 10403S infected group but not in the uninfected and *Lm* 10403SΔ*actA* infected groups are shown. **b** Functional annotation clustering of the enriched Gene Ontology (GO) terms in the molecular function (MF) category for the 320 ABI1-interacting protein candidates enriched in the *Lm* 10403S group but not in the uninfected and *Lm* 10403SΔ*actA* infected groups. Over-representation analysis was conducted based on a one-sided hypergeometric test to assess whether the input gene set was significantly enriched in MF GO category. *P* values were adjusted for multiple testing using the Benjamini-Hochberg method and GO terms with an adjusted *P* value < 0.05 were considered significant. For visualization, -log_10_-transformed *P* values were plotted. The corresponding *P*-values (pink bars) for each enriched GO MF term are shown, and the yellow connecting line indicates the percentage of gene ratio. **c** HeLa cells expressing mNeonGreen-ABI1and mCherry-EPS8 were infected with BFP-*Lm*. F-actin was visualized using SiR-actin stain. Enlarged images depict *Lm* with actin clouds, comet tails and within protrusions. Arrowheads mark *Lm* associated with actin clouds, comet tails and protrusions. Scale bar=10 μm. **d** Recruitment of mNeonGreen-ABI1 and mCherry-EPS8 to actin clouds (Cloud), comet tails, and *Lm*-containing protrusions from (**c**). EPS8 or ABI1 recruitment was calculated as the number of mCherry-EPS8-positive or mNeonGreen-ABI1-positive Clouds, comet tails or *Lm*-containing protrusions divided by the total number of quantified Clouds, comet tails or *Lm*-containing protrusions within a single infected cell (*N* = 20 cells). Data are presented as mean ± SD with all data points shown and the percent EPS8 or ABI1 recruitment indicated. **e** HeLa cells were transfected with control (NC) endoribonuclease-prepared small interfering RNA (esiRNA) or esiRNA targeting *EPS8*, *EPS8L2*, or both *EPS8* and *EPS8L2*. Forty-eight hours post-transfection, cells were infected with *Lm* 10403S and plaque formation at 72 hpi visualized as previously described. **f** Plaque size measurements from (**e**). *N* = 30 plaques. Data are presented as mean ± SD with all data points shown and percent reduction in plaque size indicated. **g** HeLa cells were transfected with plasmids encoding EPS8, EPS8ΔCap, EPS8ΔBund, EPS8ΔCapΔBund, EPS8ΔA, or an empty vector (EV) as a negative control. Forty-eight hours post-transfection, cells were infected with *Lm* 10403S and plaque formation at 72 hpi visualized as previously described. **h** Plaque size measurements from (**g**). *N* = 30 plaques. Data are presented as mean ± SD with all data points shown and the percent increase (+) or reduction (−) in plaque size compared with the EV group indicated. Welch’s one-way ANOVA (unequal variance) analysis followed by Dunnett’s T3 post-hoc test was performed to assess statistical significance (**f**, **h**). Exact *P* values are shown in the figures. Source data are provided as a Source Data file.

Given that ABI1 lacks direct actin-binding capacity, its role in regulating local actin recycling within protrusions likely involves indirect mechanisms. Therefore, to identify ABI1 interaction partners that are specifically associated with *Lm*-containing protrusion formation, we next examined the interactome of ABI1 during *Lm* infection using Quantitative Immunoprecipitation Mass Spectrometry (Quant IP-MS) analysis (Supplementary Fig. 7a). Briefly, *HA-ABI1* cells were infected with either *Lm* 10403S or 10403SΔ*actA* for 6 hours, followed by DSP (dithiobis(succinimidyl propionate)) crosslinking to stabilize weak and transient protein-protein interactions^53^. HA-ABI1 interaction partners were co-immunoprecipitated, digested, TMT (Tandem mass tag) labeled, and analyzed by mass spectrometry. A total of 3207 proteins were detected from the analysis and different ABI1 interaction partners were revealed after *Lm* infection (Supplementary Fig. 7b). Known ABI1 interaction partners, such as WASF2 and ENAH, were detected from uninfected control groups and *Lm-*infected groups (Supplementary Fig. 7c–e). A total of 320 proteins were highly enriched in *Lm*-infected groups but not in uninfected or *Lm* 10403SΔ*actA*-infected samples, and gene ontology (GO) enrichment analysis showed that these enriched proteins are mainly involved in binding actin, cadherin, ubiquitin protein ligase, as well as misfolded protein molecular function groups (Fig. 5a, b). Of particular interest, EPS8 and EPS8L2 were highly enriched after *Lm* infection compared with uninfected and *Lm* 10403SΔ*actA* infected groups (Fig. 5a). The EPS8 family, comprising EPS8, EPS8L1, EPS8L2, and EPS8L3, functions as part of the EGFR signaling pathway^54^. Notably, *EPS8* appears to be the predominant isoform in HeLa cells while *EPS8L2* is also expressed, albeit at a lower level (Supplementary Fig. 8a).

EPS8, the most characterized isoform, is a multi-domain protein (Supplementary Fig. 8b) and has diverse functions in actin dynamics^55^. EPS8 can crosslink actin filaments through dimerization and direct F-actin side binding and influences actin-based motility by efficiently capping barbed ends if associated with ABI1^24^. Moreover, EPS8 has been observed to localize specifically with actin comet tails during *Lm* intracellular motility, indicating a potential role in actin-based motility^24^. Furthermore, EPS8 family members have at least partially redundant functions, with conserved ABI1-binding sites and shared F-actin capping and bundling activities for EPS8, EPS8L1, and EPS8L2 (Supplementary Fig. 8b–d)^54^. Thus, we hypothesized that ABI1, in coordination with EPS8 (and EPS8L2), is recruited to *Lm*-containing protrusions to regulate actin dynamics for efficient cell-to-cell spread.

Co-immunoprecipitation (Co-IP) experiments confirmed that ABI1 interacts with EPS8 and EPS8L2. ABI1 lacking the PP region (ABI1ΔPP) or EPS8 harboring a mutation in the SH3 domain (EPS8ΔA) disrupted this interaction (Supplementary Fig. 8e–h). Given the observations that ABI1 requires its PP region to localize to *Lm*-containing protrusions (Fig. 3e, f and Supplementary Fig. 6a–m) and that the PP region of ABI1 was necessary for interaction with EPS8 and EPS8L2 (Supplementary Fig. 8g, h), we further investigated the role of the ABI1-EPS8 complex during infection. Endogenously expressed mCherry-tagged EPS8 localized to actin clouds, comet tails, and *Lm*-containing protrusions, underscoring EPS8’s role in *Lm* actin-based motility (Fig. 5c, d, Supplementary Fig. 9a and Supplementary Movie 3). However, ABI1 only co-localized with EPS8 within *Lm*-containing protrusions, indicating that the ABI1-EPS8 interaction is regulated in a spatiotemporal manner (Fig. 5c, d and Supplementary Movie 3). To assess the role of EPS8 in *Lm* cell-to-cell spread, we conducted plaque formation assays following *EPS8* depletion using esiRNA-mediated gene silencing^56^. *EPS8* knockdown reduced the plaque size by 18%, indicating an impairment in cell-to-cell spread, albeit to a lesser extent than *ABI1* deletion (Fig. 5e, f and Supplementary Fig. 9b). Following *EPS8* knockdown, we observed an upregulation in both the transcript and protein level of EPS8L2 (Supplementary Fig. 9c, d). Transient expression of mCherry-EPS8L2 showed that subcellular localization of EPS8L2 during infection mirrored that of EPS8, prompting us to explore its functional redundancy (Supplementary Fig. 9e–h). While *EPS8L2* depletion alone did not affect plaque size, likely due to its low expression, simultaneous knockdown of both *EPS8* and *EPS8L2* resulted in a substantial 51% reduction in plaque size (Fig. 5e, f). Therefore, we conclude that EPS8 plays a major role in regulating *Lm* cell-to-cell spread, while EPS8L2 shares similar functions and can partially compensate for the loss of EPS8 expression.

*EPS8* overexpression increased the plaque size by 34%, aligning with previous findings that EPS8 enhances optimal actin-based motility (Fig. 5g, h)^24^. To dissect which EPS8 activity influences plaque size, we expressed EPS8 mutants deficient in either capping (EPS8ΔCap) or bundling activities (EPS8ΔBund) (Supplementary Figs. 8d, e and 9i)^32^. Intriguingly, expression of capping-deficient EPS8 (EPS8ΔCap) reduced the plaque size by 51%, while bundling-deficient EPS8 (EPS8ΔBund) increased plaque size by 31%, suggesting that the distinct activities of EPS8 have opposing effects on cell-to-cell spread (Fig. 5g, h). Furthermore, expressing an EPS8 mutant lacking ABI1-binding capability (EPS8ΔA) (Supplementary Figs. 8c, e and 9i) reduced the plaque size by 43%, highlighting the importance of the ABI1-EPS8 interaction for effective *Lm* cell-to-cell spread (Fig. 5g, h). Combined with co-localization analysis, our results indicate that EPS8 forms a complex with ABI1 during protrusion formation, where EPS8 transitions to a capping role to promote efficient cell-to-cell spread.

### LLO-induced Ca²⁺ influx activates calpain to cleave spectrin and alter ABI1 dynamics

**Fig. 6:**
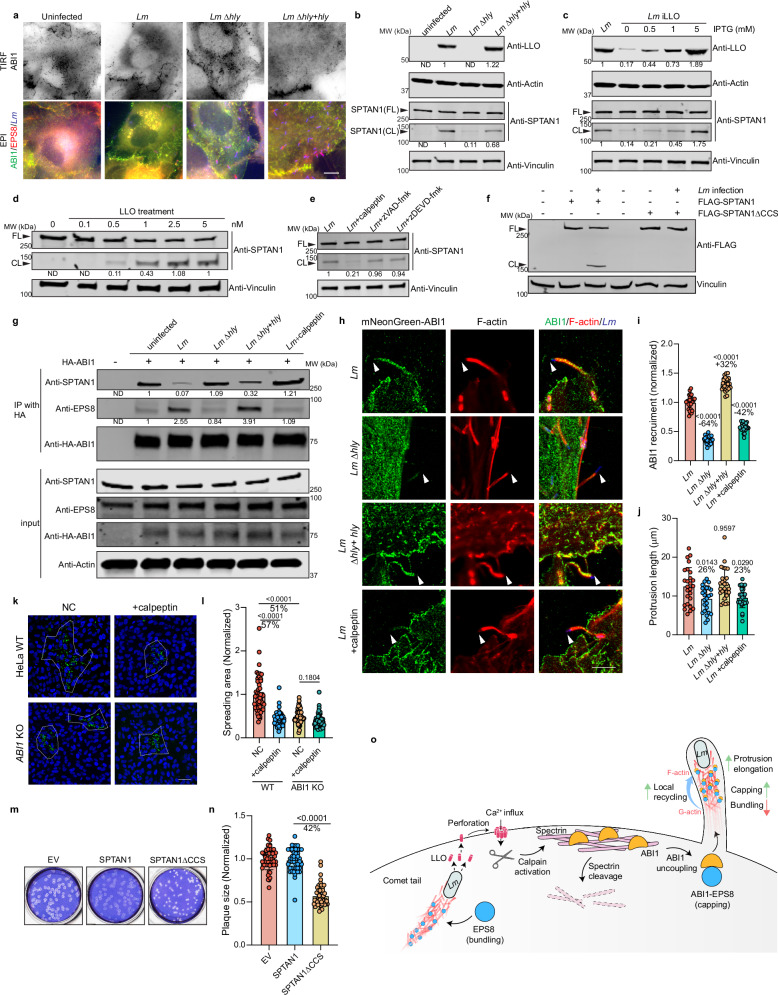
LLO triggers ABI1 complex switching via calpain-mediated spectrin cleavage to promote cell-to-cell spread. **a** HeLa cells expressing mNeonGreen-ABI1 (green) and mCherry-EPS8 (red) were infected with *Lm* 10403S (*Lm*), *Lm* Δ*hly*, or complemented *Lm* Δ*hly+hly* strains expressing TagBFP (blue). Images were captured at 5–7 hpi using either TIRF (upper panels) or epifluorescence (EPI, lower panels) microscopy. Vacuolar escape of the *Lm* Δ*hly* strain is clearly indicated by EPS8 recruitment. Scale bar=10 μm. **b** Western blot analysis of SPTAN1 cleavage in HeLa cells at 6 hpi with *Lm* 10403S (*Lm*), *Lm* Δ*hly*, or complemented *Lm* Δ*hly+hly* strains. Full-length (FL) or cleaved (CL) SPTAN1 were detected using an anti-SPTAN1 antibody with Vinculin as a loading control. LLO secretion during *Lm* infection was assessed using an anti-LLO antibody with β-Actin as a loading control. LLO and SPTAN1(CL) band intensities are shown below the respective bands. LLO band intensities were normalized to actin, and SPTAN1(CL) band intensities were normalized to Vinculin, with the *Lm* infection sample set to 1. ND=not detected. **c** Western blot analysis of SPTAN1 cleavage in HeLa cells at 6 hpi with *Lm* 10403S (*Lm*) or *Lm* iLLO at the indicated IPTG concentrations. Full-length (FL) or cleaved (CL) SPTAN1 were detected using an anti-SPTAN1 antibody with Vinculin as a loading control. LLO secretion during *Lm* infection was assessed using an anti-LLO antibody with β-Actin as a loading control. LLO and SPTAN1(CL) band intensities are shown below the respective bands. LLO band intensities were normalized to actin, and SPTAN1(CL) band intensities were normalized to Vinculin, with the *Lm* infection sample set to 1. ND=not detected. **d** Western blot analysis of SPTAN1 cleavage in HeLa cells treated for 30 minutes with purified recombinant LLO at the indicated concentrations. Full-length (FL) or cleaved (CL) SPTAN1 were detected using an anti-SPTAN1 antibody with Vinculin as a loading control. SPTAN1(CL) band intensities are shown below the respective bands. SPTAN1(CL) band intensities were normalized to Vinculin, with the 5 nM LLO treatment sample set to 1. ND=not detected. **e** Western blot analysis of SPTAN1 cleavage in HeLa cells at 6 hpi with *Lm* in the presence of calpeptin (20 μM, calpain inhibitor), zVAD-fmk (20 μM, pan-caspase inhibitor) or zDEVD-fmk (30 μM, caspase-3 inhibitor). Full-length (FL) or cleaved (CL) SPTAN1 were detected using an anti-SPTAN1 antibody with Vinculin as a loading control. SPTAN1(CL) band intensities are shown below the respective bands. SPTAN1(CL) band intensities were normalized to Vinculin, with the *Lm* infection sample set to 1. **f** Western blot analysis of SPTAN1 cleavage in WT Hela cells or HeLa cells expressing FLAG-SPTAN1 or calpain cleavage-resistant FLAG-SPTAN1ΔCCS infected with *Lm* for 6 hours. Full-length (FL) or cleaved (CL) SPTAN1 were detected using an anti-FLAG antibody with Vinculin as a loading control. **g** Co-immunoprecipitation analysis of ABI1 interaction with SPTAN1 and EPS8 in WT HeLa cells or HeLa cells expressing HA-tagged ABI1. Cells were left uninfected or infected with *Lm* 10403S (*Lm*), *Lm* Δ*hly*, complemented *Lm* Δ*hly+hly* or *Lm* with calpeptin treatment (20 μM). At 6 hpi, cells were crosslinked with DSP and lysates were immunoprecipitated with anti-HA beads and probed with anti-SPTAN1, anti-EPS8, and anti-HA antibodies. Input lysates were analyzed in parallel for expression of the indicated proteins. β-Actin served as a loading control. SPTAN1 and EPS8 band intensities are shown below the respective bands. Band intensities were normalized to HA-ABI1, with the uninfected sample set to 1. ND=not detected. **h** Representative airyscan confocal images of HeLa cells expressing mNeonGreen-ABI1 (green) infected with TagBFP expressing (blue) *Lm*, *Lm* Δ*hly*, complemented *Lm* Δ*hly+hly* or *Lm* in the presence of calpeptin (20 μM). Cells were fixed at 6 hpi and F-actin was stained with Texas Red™-X Phalloidin (red). Arrowheads indicate *Lm*-containing protrusions. Scale bar=5 μm. **i** Quantification of ABI1 recruitment to protrusions from (**h**). ABI1 recruitment was determined by measuring the average fluorescence intensity of mNeonGreen within visibly apparent BFP-*Lm* associated actin comet tails extending from the cell body (protrusions). Values are normalized to the *Lm* infection group. Data are presented as mean ± SD with all data points shown and percent increase (+) or reduction (−) relative to the *Lm* infection group indicated. *N* = 26 protrusions. **j** Quantification of protrusion length from (**h**, **i**). Data are presented as mean ± SD with all data points shown and percent reduction compared to the *Lm* infection group indicated. *N* = 26 protrusions. **k**,**l** Representative images (**k**) and quantification (**l**) from an infection focus assay assessing *Lm* cell-to-cell spread at 6 hpi in WT or *ABI1 KO* HeLa cells in the absence (NC) or presence of calpeptin (20 μM). Dotted lines delineate the edges of infection foci. Scale bar=50 μm. Data are presented as mean ± SD with all data points shown and percent reduction in spreading area indicated. *N* = 50 foci. **m** HeLa cells were transfected with plasmids encoding FLAG-SPTAN1, FLAG-SPTAN1ΔCCS or an empty vector (EV) as a negative control. Forty-eight hours post-transfection, cells were infected with *Lm* 10403S and plaque formation at 72 hpi visualized as previously described. **n** Plaque size measurements from (**m**). *N* = 50 plaques. Data are presented as mean ± SD with all data points shown and the percent reduction in plaque size compared with the SPTAN1 group indicated. **o** Proposed model for LLO-initiated ABI1 complex switching to promote protrusion elongation. After escaping the vacuole, *Lm* initiates actin polymerization to drive intracellular motility and recruits EPS8 to stabilize actin filaments (bundling activity). *Lm* secretes the pore-forming toxin LLO into the host cytosol, which perforates the PM and triggers Ca^2+^ influx. This influx activates calpain, leading to cleavage of the spectrin cytoskeleton and destabilization of ABI1-spectrin interaction beneath the PM. During protrusion formation, *Lm* engages the released ABI1 at cell-cell junctions, where ABI1 forms a complex with EPS8 and converts EPS8 from an actin-bundling protein to a barbed-end capping protein (capping activity). This functional switch enhances local actin recycling within protrusions, facilitating protrusion elongation and efficient cell-to-cell spread. One-way ANOVA (equal variance) analysis followed by Tukey’s post-hoc test was performed to assess statistical significance (**i**, **j**, **l**, **n**). Exact *P* values are shown in the figures. Source data are provided as a Source Data file.

Next, we examined the mechanism of ABI1 recruitment to *Lm*-containing protrusions. Ca^2+^ influx has been shown to redistribute ABI1 at the cell periphery^57^. Under resting conditions, ABI1 appeared mainly as stable puncta, with a smaller fraction of a fast-diffusing cortical pool beneath the PM (Fig. 6a and Supplementary Movie 4). Treatment with a Ca^2+^ ionophore (ionomycin) greatly reduced the proportion of stationary puncta and increased the fast-diffusing cortical pool, as visualized by TIRF microscopy (Supplementary Fig. 10a and Supplementary Movie 4). This ionomycin-induced ABI1 redistribution was abolished by co-treatment with the calcium chelator (EGTA), indicating a dependence on Ca^2+^ influx. Subsequent to host cell invasion, *Lm* escapes the phagocytic vacuole primarily through secretion of LLO. However, in human-derived epithelial cells, vacuole escape can be facilitated by the *Lm* phospholipase C (PC-PLC), and LLO is dispensable for *Lm* access to the cytosol and bacterial replication^58–60^. Moreover, LLO is continuously secreted by *Lm* during replication in the host cytosol, where it can perforate the PM and trigger Ca^2+^ influx^21,61^. Notably, *Lm* 10403S infection recapitulated the ionomycin-induced changes in ABI1 dynamics, whereas infection with a LLO-deficient mutant (*Lm* Δ*hly*) had no detectable effect on ABI1 localization, despite similar numbers of bacteria in the host cell cytosol (Fig. 6a, Supplementary Fig. 10a and Supplementary Movie 5). Infection with a complemented *Lm* Δ*hly*+*hly* strain yielded changes in ABI1 dynamics similar to that observed with *Lm* 10403S (Fig. 6a).

The spectrin-based membrane skeleton supports and stabilizes specialized PM structures within host cells. Furthermore, ABI1 has been reported to interact with αII-spectrin (SPTAN1)^62^. In addition to ionomycin-induced changes in ABI1 dynamics (Supplementary Fig. 10a), ionomycin treatment rapidly induced SPTAN1 cleavage, an effect that was blocked by EGTA (Supplementary Fig. 10b). Interestingly, SPTAN1 was cleaved during *Lm* infection, and this cleavage was largely abolished in cells infected with *Lm* Δ*hly*, implicating LLO in the process leading to SPTAN1 cleavage (Fig. 6b). Collectively, these data suggest that Ca^2+^ influx triggered by LLO could lead to SPTAN1 cleavage resulting in changes in ABI1 localization dynamics. Using an inducible LLO-expressing *Lm* strain^63–65^, in which LLO production is controlled by IPTG, we observed an LLO concentration-dependent increase in SPTAN1 cleavage (Fig. 6c). In addition, treatment of host cells with purified recombinant LLO similarly induced SPTAN1 cleavage in a concentration-dependent manner (Fig. 6d and Supplementary Fig. 10c). Moreover, addition of a sublytic concentration of purified LLO reproduced the ABI1 localization dynamic changes observed with ionomycin or *Lm* infection (Supplementary Fig. 10d and Supplementary Movie 6).

Because SPTAN1 can be targeted by caspases or Ca^2+^-activated calpains^66^, we used inhibitors against both protease families to determine if they mediated SPTAN1 cleavage during *Lm* infection. Calpeptin (a calpain inhibitor) blocked *Lm*-induced SPTAN1 cleavage, whereas zVAD-fmk (a pan-caspase inhibitor) or zDEVD-fmk (a caspase-3 inhibitor) had no effect, indicating Ca^2+^-activated calpains are responsible for SPTAN1 cleavage during *Lm* infection (Fig. 6e and Supplementary Fig. 10e). This conclusion was further supported by expressing a calpain cleavage-resistant SPTAN1 mutant lacking the calpain cleavage sequence (SPTAN1ΔCCS) (Supplementary Fig. 10f). Notably, SPTAN1ΔCCS was resistant to *Lm*-induced cleavage but remained susceptible to caspase-dependent cleavage upon treatment with the caspase-3 activator raptinal but not Ca^2+^ influx triggered by ionomycin (Fig. 6f and Supplementary Fig. 10 g, h).

### ABI1 complex switching facilitates protrusion extension and cell-to-cell spread

Since the ABI1 PP region interacts with the SH3 domain of SPTAN1^31,62^ and the calpain cleavage site in SPTAN1 lies adjacent to its SH3 domain (Supplementary Fig. 10 f), we hypothesized that SPTAN1 cleavage might promote a complex switch, releasing ABI1 from the spectrin cytoskeleton and enabling the ABI PP region to interact with the EPS8 SH3 domain (Supplementary Figs. 5c and 8e). This binding partner switch would allow for activation of EPS8 actin-capping activity, thereby facilitating protrusion elongation. Consistent with this hypothesis, co-immunoprecipitation revealed that *Lm* infection enhanced ABI1-EPS8 interaction, whereas ABI1-SPTAN1 interaction predominated during *Lm* Δ*hly* infection or *Lm* infection with calpeptin treatment (Fig. 6g). This result was also supported by microscopy observations that despite similar numbers of cytosolic *Lm*, ABI1 recruitment to protrusions was markedly reduced in cells infected with *Lm* Δ*hly* (64% reduction) and in calpeptin-treated cells (42% reduction) (Fig. 6h, i). Moreover, infection with *Lm* Δ*hly* resulted in protrusions that were 26% shorter, an effect closely mirrored by calpeptin treatment (23% reduction in protrusion length) (Fig. 6j). Infection foci analysis showed that calpeptin treatment impaired cell-to-cell spread in HeLa WT cells (57% reduction), but not in *ABI1* KO cells (Fig. 6k, l). The inability of calpeptin treatment to cause a further spreading defect in *ABI1* KO cells indicates that ABI1 functions downstream of SPTAN1 cleavage in promoting *Lm* cell-to-cell spread (Fig. 6k, l and Supplementary Fig. 10i). Furthermore, expression of the calpain cleavage-resistant SPTAN1ΔCCS resulted in a reduction in plaque size by 42% compared to SPTAN1 (Fig. 6m, n). Collectively, these results highlight the importance of LLO-induced calpain activation and spectrin cleavage for initiating ABI1 complex switching from ABI1-SPTAN1 to ABI1-EPS8 to facilitate protrusion elongation and *Lm* cell-to-cell spread.

## Discussion

Our findings support a model (Fig. 6o) in which *Lm*-containing protrusions are not passive byproducts of actin-based motility but rather dynamic, actively regulated structures optimized for cell-to-cell spread^67^. Following vacuole escape, *Lm* expresses ActA to drive ARP2/3-dependent actin polymerization and engages EPS8 to stabilize actin structures during cytosolic motility. Concurrently, continuous secretion of the pore-forming toxin LLO perforates the host PM, elevating intracellular Ca^2+^ levels resulting in the activation of calpain proteases. Activated calpains cleave SPTAN1, releasing ABI1 from its spectrin cytoskeleton anchor beneath the PM. As bacteria approach cell-cell junctions, ABI1 is actively recruited into *Lm*-containing protrusions, where ABI1 forms a complex with EPS8. The ABI1-EPS8 complex interaction switches EPS8 activity from an actin-bundling factor into a barbed-end actin capping protein. This functional switch in EPS8 activity promotes local actin recycling by destabilizing bundled filaments and capping filament barbed ends near the bacterial pole yielding an increase in the available free actin pool within the *Lm*-containing protrusion. Such dual activity becomes particularly critical in generating long *Lm*-containing protrusions, which can extend up to ~100 μm and where local actin availability is limiting^13,68^. We propose that the ABI1-EPS8 complex supports an “actin funneling” mechanism^69,70^, directing recycled actin monomers toward available barbed ends to sustain filament assembly and maintain protrusion elongation (Fig. 6o).

Although our data indicates that ABI1 acts upstream of EPS8 to regulate protrusion dynamics, they do not exclude partially independent, context-dependent roles for these factors during *Lm* infection. Future genetic epistasis analyses, such as combined perturbation of ABI1 and EPS8, will be required to further define their functional relationship and coordination in supporting efficient *Lm* cell-to-cell spread. Our model suggests that LLO-induced extracellular Ca^2+^ influx contributes to ABI1 redistribution during infection; however, Ca^2+^ influx alone may not fully account for all aspects of ABI1 dynamics. Additional LLO-triggered, Ca^2+^-independent signaling pathways, such as membrane damage sensing or stress-responsive mechanisms, may also contribute to ABI1 redistribution and warrants further investigation.

Pore-forming toxins (PFTs) are classically recognized for breaching host membranes, causing cytolysis, or subverting immune responses. While such destructive activities are central to many pathogen virulence strategies, our findings uncover an additional unrecognized function. Rather than acting through sustained or global cytotoxicity, we propose that LLO functions in a highly localized and transient manner at the host PM to promote bacterial cell-to-cell spread. LLO activity is known to be tightly regulated by factors such as cholesterol-dependent membrane binding, pH sensitivity, and host-mediated clearance mechanisms, including endocytic pathways that limit toxin persistence at the plasma membrane. Within this constrained spatial and temporal window, restricted LLO pore formation induces localized Ca^2+^ influx sufficient to activate calpain-dependent spectrin cleavage, thereby enhancing local actin recycling within *Lm*-containing protrusions. This mechanism illustrates how pathogens can couple spatially confined membrane perforation to precise modulation of host actin dynamics. In this context, LLO pore formation is not merely destructive but is tightly regulated and integrated into a mechanism to fine-tune host cell architecture in a manner that favors efficient bacterial dissemination while limiting broader cytotoxicity.

Spectrins are ubiquitous, membrane-associated cytoskeletal scaffolds in metazoan cells. Spectrins are localized beneath the PM where they interact with actin and scaffolding partners such as ABI1, thereby supporting cortical stability, membrane organization, and barrier function^71^. By targeting spectrin, *Lm* exploits a structural element that is essential for cellular integrity yet also linked to cytoskeletal plasticity. Spectrin breakdown is also a recurring theme in human disease, where spectrin breakdown products (SBDPs) accumulate in traumatic brain injury, stroke, and neurodegeneration, serving as biomarkers of cellular damage^30^. Aging is also associated with increased spectrin fragility, raising the possibility that increased spectrin breakdown predisposes elderly individuals to severe *Lm* infections. Indeed, older adults suffer disproportionately from *Lm* invasive disease and mortality^72^, and spectrin deterioration may be a significant component of this susceptibility. Furthermore, given the role of spectrin in membrane barrier integrity, proteolysis of spectrin could facilitate *Lm* traversal of the intestinal epithelium and blood-brain barrier, offering a mechanistic basis for the central nervous system infections observed in elderly hosts.

“Complex switching” is a pervasive regulatory principle across biological systems, including signal transduction^73^, cytoskeletal rearrangement^74^, and transcriptional regulation^75^. Our findings highlight that complex switching regulation can be actively exploited by a bacterial pathogen, with ABI1 serving as a versatile signaling hub capable of partnering with diverse effectors^76^. ABI1 can assemble into distinct complexes, including: the ABI1-EPS8 complex, which caps actin barbed ends^24^; the ABI1-SOS1-p85 complex, which acts as a Rac guanine nucleotide-exchange factor to initiate cytoskeletal rearrangements^77^; and the ABI1-WAVE2 complex, which activates ARP2/3 to drive actin nucleation in lamellipodia^28^. *Lm* exploits the flexibility in ABI1 protein complex formation by rerouting ABI1 from a PM spectrin cytoskeleton localization to association with EPS8 in *Lm*-containing protrusions. This pathogen-driven complex switching underscores how microbes act as master cell biologists, exploiting the regulatory plasticity of host scaffolds to orchestrate localized actin remodeling. Beyond infection, the controlled recruitment of ABI1 has parallels in fundamental physiological processes involving actin-based motility, including lamellipodia formation at the leading edge^78^, mitochondrial inheritance during cell division^79^, and propulsion of PtdIns(4,5)P_2_-enriched vesicles^80^. Understanding how ABI1 complex formation is dynamically fine-tuned in response to cellular signals may therefore illuminate both host-pathogen interactions and core mechanisms of cell motility and membrane dynamics.

Finally, our findings highlight potential therapeutic avenues against infections by intracellular pathogens. Inhibiting the ABI1-EPS8 interaction, stabilizing spectrin, or blocking calpain activity may limit *Lm* dissemination. More broadly, because many pathogens exploit actin-based motility, the ABI1-EPS8 complex may represent a shared vulnerability that could be selectively targeted without broadly disrupting host cytoskeletal function. Indeed, ABI1 and EPS8 have also been implicated in *Shigella flexneri* pathogenesis^24,81^, suggesting the ABI1-EPS8 interaction may be co-opted across diverse microbes, including *Rickettsia*, *Burkholderia*, and vaccinia virus that all spread cell to cell using actin-based motility. Using *Lm* as a model intracellular pathogen, our study positions the ABI1-EPS8 interaction as a potential target for future therapeutic intervention against multiple intracellular pathogens.

## Methods

### Ethics Statement

All research was conducted in compliance with all relevant institutional, national, and international regulations. Microbiological work was approved by the Harvard University Committee on Microbiological Safety (COMS; protocol no. 25-097). All animal experiments were conducted in accordance with Association for Assessment and Accreditation of Laboratory Animal Care International (AAALAC International) guidelines and were approved by the Harvard Medical School Institutional Animal Care and Use Committee (IACUC; protocol no. IS-065-9), in compliance with all applicable federal, state, and local regulations.

### Animals

*Esr1-cre* mice (B6.Cg-Tg(CAG-cre/Esr1*)5Amc/J, The Jackson Laboratory, Cat.# 004682) and *Mx1-cre* mice (B6.Cg-Tg(Mx1-cre)1Cgn/J, The Jackson Laboratory, Cat.# 003556) were bred in-house at The Harvard Institutes of Medicine BL-1 Animal Facility at Harvard Medical School. *Abi1*^*fl/fl*^ mice (Floxed alleles for *Abi1*) were kindly provided by Dr. Leszek Kotula (SUNY Upstate Medical University)^29^. All experiments were performed with 7-12-week-old sex- and age-matched mice that were maintained on a 12-hour light-dark cycle, with food and water available *ad libitum*. All animals were bred separately and housed in specific pathogen-free barrier rooms kept at 22 °C–26 °C and 45–55% humidity. Experiments were not blinded, and mice were not randomized. All animal care and experiments were conducted in compliance with the Association for Assessment and Accreditation of Laboratory Animal Care International regulations. All experimental protocols (IACUC; protocol no. IS-065-9) were approved by the Harvard Medical School Institutional Animal Care and Use Committee and were in compliance with all federal, state and local laws.

### Bacterial culture

*Listeria monocytogenes* (*Lm*) was grown in filter-sterilized brain-heart infusion (BHI) broth (BD DIFCO^TM^, Cat.# 237200) and the following strains were used: 10403S, 10403SΔ*actA*, 10403SΔ*hly*, 10403SΔ*hly+hly*, 10403S expressing TagBFP, 10403SΔ*hly* expressing TagBFP, 10403SΔ*hly+hly* expressing TagBFP, 10403S expressing GFP, 10403S expressing mScarlet, LS743, LS743Δ*actA*, and 10403SΔ*hly* + iLLO (*Lm* iLLO). *Escherichia coli* strains were grown in Lysogeny Broth (LB) medium. Antibiotics were used at the following concentrations: streptomycin (VWR, Cat.# 0382-50 G) (100 μg/mL) for *Lm* 10403S-derived strains; nalidixic acid (Millipore Sigma, Cat.# N8878) (50 μg/mL) for *Lm* LS743-derived strains; chloramphenicol (VWR, Cat.# 0230-100 G) (20 μg/mL) for selection of pLOV-derived plasmids in *E. coli*, chloramphenicol (7.5 μg/mL) for selection of pAM401-derived plasmids in *Lm*, chloramphenicol (7.5 μg/mL) for selection of single-copy pLOV-derived plasmids integrated at the tRNA^ARG^ locus in *Lm*, and erythromycin (Millipore Sigma, Cat.# E6376) (3 μg/mL) with lincomycin (RPI, Cat.# L22020-5.0) (25 μg/mL) for selection of the single-copy pHHFe plasmid integrated at the tRNA^ARG^ locus in *Lm*.

### Cell Lines

HeLa (female, Cat.# CCL-2), HEK293T (female, Cat.# CRL-3216), HepG2 (male, Cat.# HB-8065), JEG-3 (female, Cat.# HTB-36), THP-1 (male, Cat.# TIB-202), L929 (female, murine, Cat.# CCL-1), hCMEC/D3 (female, Millipore Sigma, Cat.# SCC066), and Caco2 (male, Cat.# HTB-37) cell lines were originally obtained from ATCC or commercial vendors as indicated, or from academic sources (Dr. Judy Lieberman and Dr. Marcia Goldberg). Cell lines generated in this study include *ABI1* knockout (KO), knockdown (KD), and stable expression derivatives of HeLa, Caco2, HepG2, JEG-3, hCMEC/D3, and THP-1 cells, as well as HeLa cells expressing tagged ABI1, EPS8, EZRIN, or mEOS3.2-actin constructs. All derivative cell lines retain the sex of the parental line. Bone marrow-derived macrophages (BMDM) were generated from C57BL/6J mice using standard differentiation protocols. Both male and female mice were used for BMDM isolation.

HeLa, Caco2, HepG2, JEG-3, and HEK293T cells were grown in Dulbecco’s modified Eagle’s medium (DMEM) (Gibco, Cat.# 11995-065) supplemented with 10% fetal bovine serum (FBS) (Gibco, Cat.# 10437-028). THP-1 cells were grown in RPMI 1640 medium (CORNING, Cat.# 10-040-CV) supplemented with 10% FBS. hCMEC/D3 cells were grown in complete EndoGRO-MV Basal Medium (endothelial cell medium, ECM) containing 5% (v/v) FBS, 0.2% (v/v) EndoGRO-LS supplement, 5 ng/ml recombinant human epidermal growth factor, 10 mM L-glutamine, 1 μg/mL hydrocortisone-hemisuccinate, 0.75 U/mL heparin-sulfate, and 50 μg/ml ascorbic acid (Millipore Sigma, Cat.# SCME004).

Bone marrow-derived macrophages (BMDM) were cultured as previously described^82^. Briefly, 8-12-week-old mice were euthanized and femurs removed. Bone marrow was then flushed from the femurs with DMEM with 100 μg/mL penicillin-streptomycin (Gibco, Cat.# 15140122). Cells were then cultured in BMM medium (DMEM supplemented with 10% FBS, 2 mM glutamine (Gibco, Cat.# 25030081), 1 mM sodium pyruvate (Corning, Cat.# 25-000-CI), 100 μg/mL penicillin-streptomycin (Gibco, Cat.# 15140122), 55 μM β-mercaptoethanol (Gibco, Cat.# 21985023), and 30% L929-cell conditioned medium) in 150 mm non-tissue-culture treated petri dishes (Nalge Nunc International). On day 3, the culture medium was replaced with fresh BMM medium. On day 7, media was removed from the cells and BMDM were harvested.

All cells were maintained at 37 °C in 5% CO_2_. In the following sections, “cell culture medium” refers to the growth medium designated for each cell line mentioned above, unless otherwise specified.

### Bacterial strains

To generate *Lm* strains expressing TagBFP, the reporter construct pLOV-TagBFP was generated by digestion and ligation cloning. Primers were designed and used to amplify the *Lm*-codon optimized TagBFP sequence from genomic DNA isolated from the pEB400::10403S *Lm* strain^83^ and ligated to the pLOV empty vector using PstI and KpnI. The resulting plasmid pLOV-TagBFP was electroporated into *Lm* to generate the *Lm* 10403S-derived strains expressing TagBFP. To generate the 10403SΔ*hly+hly* strain expressing TagBFP, the pHHFe plasmid was electroporated into 10403SΔ*hly* to yield 10403SΔ*hly+hly* encoding erythromycin resistance. The pAM-Spac-TagBFP plasmid was then electroporated into erythromycin-resistant 10403SΔ*hly+hly* to yield the 10403SΔ*hly+hly* strain expressing TagBFP. The strain was grown in the presence of chloramphenicol (7.5 μg/mL), erythromycin (3 μg/mL) with lincomycin (25 μg/mL) to maintain the episomal pAM-Spac-TagBFP plasmid. To generate an isogenic *actA* deletion mutant of *Lm* LS743, the *actA* gene was deleted from the genome of *Lm* LS743. The pKSV7-Δ*actA* plasmid was electroporated into LS743 and allelic exchange was performed to generate strain *Lm* LS743Δ*actA*. All bacterial strains and plasmids used are listed in Supplementary Data 1. All primers used are listed in Supplementary Data 2.

### Protein Expression and Purification

Recombinant C-terminal 6xHis-tagged LLO protein lacking its secretion signal (amino acids 1–24) was heterologously expressed in *E. coli* JM109(DE3) carrying the plasmid pET29b-LLO-6xHis. A single colony was inoculated into 25 mL of LB medium containing 30 μg/mL kanamycin (Millipore Sigma, Cat.# K4000) and grown 16–18 h at 37 °C with shaking at 220 rpm. A 10 mL aliquot of the culture was subsequently diluted into 500 mL of fresh LB medium containing 30 μg/mL kanamycin and incubated at 37 °C with shaking at 220 rpm for 90 minutes. LLO protein expression was then induced by adding 5 mM isopropyl β-D-1-thiogalactopyranoside (IPTG) (Adipogen, Cat.# AG-CC1-0002-G025), and cultures were incubated at 30 °C for an additional 6 h with shaking at 180 rpm. The bacterial cells were harvested by centrifugation and stored at −80 °C until further purification.

The bacterial pellet was thawed in warm water and resuspended in 40 mL of ice-cold Sonication Buffer (50 mM phosphate, pH 8, 1 M NaCl, 1 mM PMSF (Roche, Cat.# 10837091001)). Cells were lysed by pulse sonication (10 sec ON/10 sec OFF, 40% output) for 15 min on ice. Lysates were clarified by centrifugation at 12,000 x *g* for 20 minutes at 4 °C, and the resulting supernatant containing LLO-6xHis was loaded onto a 3 mL pre-equilibrated HisPur^TM^ Cobalt Spin Column (Thermo Scientific^TM^, Cat.# 89969). The column was washed with Wash Buffer (50 mM phosphate/acetate pH 6, 1 M NaCl, 0.1% Tween-20 (Promega, Cat.# H5151), 10% glycerol (Aqua Solutions, Cat.# G7500-1L)) until the A280 of the flow-through was <0.01. Bound protein was eluted with 6 mL Elution Buffer (50 mM phosphate/acetate, pH 6, 1 M NaCl, 800 mM imidazole (Millipore Sigma, Cat.# I2399)). Eluates were dialyzed for 16-20 hours against 1 L of Storage Buffer (50 mM phosphate/acetate, pH 6, 1 M NaCl, 1 mM EDTA (VWR, Cat.# BDH-7830-1L), 5 mM DTT (Alfa Aesar, Cat.# A15797)) using a Slide-A-Lyzer^TM^ Dialysis Cassette (MWCO 10 K, Thermo Scientific^TM^, Cat.# 66810). The dialyzed protein was concentrated and buffer-exchanged into LLO Long Term Storage Buffer (50 mM phosphate pH 6, 500 mM NaCl, 1 mM EDTA, 5 mM DTT, 50% glycerol) using an Amicon^®^ Ultra Centrifugal Filter, 30 kDa MWCO (Millipore Sigma, Cat.# UFC903008) and stored at −80 °C. The protein concentration was determined using the Pierce™ BCA Protein Assay Kit (Thermo Scientific^TM^, Cat.# 23250). All purification steps were performed at 4 °C unless otherwise specified. The activity of the recombinant LLO was confirmed by the ability to lyse sheep red blood cells in Alsever’s solution (~ 15–20% hematocrit; Hemostat Labs, Cat.# SBA030).

### Lentivirus production

For lentiviral packaging, HEK293T cells cultured in plain DMEM were transfected with 2.5:1:1.5 of the transfer plasmid, VSV-G envelope expressing plasmid pMD2.G (Addgene, Cat.# 12259) and lentiviral packaging psPAX2 (Addgene, Cat.# 12260) using the calcium phosphate transfection kit (Takara, Cat.# 631312). After 12 h, the medium was replaced with DMEM containing 10% FBS and the cells were incubated for 48 hours for lentiviral particle production. The virus containing supernatant was collected at 24-, 36-, and 48-hours post-transfection, then filtered through a 0.45 μm syringe filter.

### CRISPR design, targeting and generation of knockout cell lines

To generate the HeLa *ABI1* knockout (KO) cell line, crRNA targeting *ABI1* (Design ID: Hs.Cas9.ABI1.1.AD) was selected from IDT’s predesigned guide RNA library and synthesized. The RNP complex, containing *ABI1*-specific crRNA, tracrRNA-ATTO™ 550 (IDT, Cat.# 075927), and HiFi Cas9 Nuclease (IDT, Cat.# 1078727) was assembled at room temperature (22 °C–25 °C) following the manufacturer’s protocol. HeLa cells were reverse transfected with assembled RNP complex using Lipofectamine CRISPRMAX (Invitrogen™, Cat.# CMAX00008) and incubated for 24 h. Single ATTO™ 550-positive cells were isolated via fluorescence-activated cell sorting (FACS) and cultured for 2-3 weeks to allow expansion. Clonal populations were screened for KO validation by immunoblotting to confirm the absence of target protein expression. To generate *ABI1* KO cell lines in Caco2, HepG2, JEG-3 and THP-1 cells, gRNA sequences targeting *ABI1* were designed using CRISPick^84^. Complementary DNA oligonucleotides encoding the crRNA sequences were annealed and cloned into a BsmBI-digested pLentiCRISPR-v2 (Addgene, Cat.# 52961) backbone using Quick Ligation^TM^ kit (NEB, Cat.# M2200S). Lentiviral particles were produced as described above and used to transduce the target cells. After transduction, cells were selected with puromycin (Gibco, Cat.# A1113803) for 7 days. Single cells were isolated by serial dilution into 96-well plates and clones were expanded over 2-3 weeks. Finally, successful *ABI1* KO clones were validated by immunoblotting to confirm the absence of the target protein expression. All primers used are listed in Supplementary Data 2.

### Generation of the hCMEC/D3 knockdown cell line

To generate the hCMEC/D3 *ABI1* knockdown (KD) cell line, shRNA sequence targeting *ABI1* was selected from the RNAi Consortium shRNA Library (Broad Institute, TRCN0000018496) and cloned into the pLKO.1 vector (Addgene, Cat.# 8453). pLKO.1-Scrambled vector (Addgene, Cat.# 136035) served as a negative control. hCMEC/D3 cells were transduced with lentiviral particles as described above. Following 14 days of selection with puromycin, polyclonal hCMEC/D3 *ABI1* KD cell lines were expanded and *ABI1* expression levels were validated by immunoblotting.

### Generation of knock-in cell lines

Adeno-associated virus integration site 1 (AAVS1) locus knockin was performed as previously described^40^. To generate *HA-ABI1* and *HA-ABI1*Δ*PP* HeLa complementation cell lines, donor plasmids pDEST-AAVS1-HA-ABI1(BlastR) or pDEST-AAVS1-HA-ABI1ΔPP(BlastR) and Cas9 plasmid targeting the AAVS1 insertion site pX459-HypaCas9-mR2-AAVS1_sgRNA (Addgene, Cat.# 183890) were transfected into HeLa *ABI1* KO cells using Lipofectamine 3000. Three days post-transfection,15 μg/mL blasticidin (InvivoGen, Cat.# ant-bl-05) was added to select for successful knockin cells. Seven days post-selection, surviving cells were collected and single cells were isolated into 96-well plates by FACS. Single colonies were expanded, and successful knockin cell lines were validated by Sanger sequencing and immunoblot analysis for *ABI1* expression.

To generate HeLa cells expressing mEOS3.2-Actin, donor plasmid pDEST-AAVS1-mEOS3.2-Actin(PuroR) and the same Cas9 plasmid targeting AAVS1 insertion site were transfected into wild-type (WT) HeLa or *ABI1* KO HeLa cells using Lipofectamine 3000. Three days post-transfection, 2 μg/mL puromycin was added to select the successful knockin cells. After seven days of selection, surviving cells were collected, and single GFP-positive cells were isolated into 96-well plates by FACS. Single colonies were expanded, and successful knockin cell lines were validated using Sanger sequencing and examined under a microscope for mEOS.2-Actin expression.

The mNeonGreen-ABI1 (mNG-ABI1) reporter HeLa cell line was generated as previously described^46^. Briefly, donor plasmid pUC19-mNeonGreen-ABI-HDR and Cas9 plasmid pSpCas9(BB)−2A-Blast-sgABI1-N targeting *ABI1* exon 1 were co-transfected into HeLa cells using Lipofectamine 3000. Three days post-transfection, single GFP-positive cells were isolated into 96-well plates by FACS. Single colonies were expanded, and successful knockin cell lines were validated by Sanger sequencing and screened under a microscope for mNeonGreen-ABI1 expression. To generate the mCherry-EPS8 reporter HeLa cell line, donor plasmid pUC19-mCherry-EPS8-HDR and Cas9 plasmid pSpCas9(BB)−2A-Blast-sgEPS8-N targeting *EPS8* exon 2 were co-transfected into HeLa cells using Lipofectamine 3000. Three days post-transfection, single mCherry-positive cells were isolated into 96-well plates by FACS. Single colonies were expanded, and successful knockin cell lines were validated by Sanger sequencing and screened under a microscope for mCherry-EPS8 expression.

To generate the EZR-TagRFP reporter cell line, an optimized CRISPaint method was used to tag endogenous EZR protein with TagRFP in mNG-ABI1 HeLa cells^49^. Donor plasmid pCRISPaint-TagRFP-PuroR (Addgene, Cat.# 80971), a modified frame selector plasmid pCAS9-Frame+1, and Cas9 plasmid pSpCas9(BB)−2A-Blast-sgEZR-C were co-transfected into mNG-ABI1 HeLa cells using Lipofectamine 3000. Three days post-transfection, single TagRFP-positive cells were isolated into 96-well plates by FACS. Single colonies were expanded, and successful knockin cell lines were validated using Sanger sequencing and screened under a microscope for EZR-TagRFP expression.

### Plaque formation assay

HeLa, HepG2, Caco2, JEG-3, or hCMEC/D3 cells were seeded at 1 × 10^6^ cells per well in 6-well plates and grown in cell culture medium for 18–24 h. *Lm* 10403S was cultured in BHI medium at 30 °C without shaking for 16 h. Diluted bacterial cultures were added to cells in 3 mL of cell culture medium (MOI of 2 for HeLa cells; MOI of 0.1 for HepG2 cells; MOI of 0.2 for Caco2 cells; MOI of 0.002 for JEG-3 cells; MOI of 0.2 for hCMEC/D3 cells). Infected cells were incubated for 1 h at 37 °C in a tissue culture incubator, washed three times with PBS and overlaid with cell culture medium containing 30 μg/mL gentamicin (IBI Scientific, Cat.# IB02030). Cells were incubated for three days to allow plaque formation. To visualize plaques, cells were washed three times with PBS and fixed in 4% paraformaldehyde (PFA) (Electron Microscopy Sciences, Cat.# 15710-S) for 15 min at room temperature and washed again three time with PBS. Cells were stained with crystal violet (HARLECO^®^, Cat.# 65092A-95) imaged, and the relative plaque areas were measured using Fiji software (Version: 2.3.0/1.54 m, NIH).

For the heterologous plaque formation assay, THP-1 cells were differentiated in RPMI medium containing 10% FBS and 50 ng/mL Phorbol 12-myristate 13-acetate (PMA) (InvivoGen, Cat.# tlrl-pma) for three days. One day prior to infection, differentiated THP-1 cells were seeded at 1 × 10^6^cells per well in 6-well plates and cultured in RPMI medium with 10% FBS. hCMEC/D3 cells were seeded at 1 × 10^6^ cells per well in collagen-coated 6-well plates and grown in complete EndoGRO-MV Basal Medium. A *Lm* 10403S bacterial culture was diluted and added to differentiated THP-1 cells in 3 mL of cell culture medium at an MOI of 50. At 1 hour post-infection, THP-1 cells were washed three times with PBS and cultured in medium containing 30 μg/mL gentamicin. At 2 hours post-infection, infected THP-1 cells were detached, resuspended in complete EndoGRO-MV Basal Medium containing 30 μg/mL gentamicin, and 1 × 10^5^ THP-1 cells/well were added to hCMEC/D3 cells. The co-cultures were incubated for 3 days to allow plaques to form. Plaques were visualized, imaged, and analyzed as described above.

### Infection focus assay

The infection focus assay was performed as previously described with minor modifications^85^. BMDM were plated at 2 × 10^6^ cells per well in 24-well glass bottom plates (Cellvis, Cat.# P24-1.5H-N) and cultured in RPMI supplemented with 10% FBS for 18–24 h. HeLa cells were plated at 2 × 10^5^ cells per well in 24-well glass bottom plates and grown in DMEM with 10% FBS for 18–24 h. Caco2 and JEG-3 cells were plated at 5 × 10^5^ cells per well in 12-well plates with glass-like polymer bottoms (Cellvis, Cat.# P12-1.5 P) and cultured in DMEM with 10% FBS for 18–24 h. Similarly, hCMEC/D3 and HepG2 cells were seeded at 5 × 10^5^ cells per well in collagen-coated 12-well plates with glass-like polymer bottom and cultured in complete EndoGRO-MV Basal Medium or in DMEM with 10% FBS, respectively, for 18–24 h. The cells were infected with *Lm* 10403S expressing GFP (MOI of 0.001 for BMDM cells; MOI of 20 for HeLa cells; MOI of 2 for HepG2 cells; MOI of 1 for Caco2 cells; MOI of 0.05 for JEG-3 cells; MOI of 10 for hCMEC/D3 cells) for 1 h in culture medium at 37 °C in a tissue culture incubator. Cells were then washed three times with PBS, and fresh medium containing 30 μg/mL gentamicin was added to kill extracellular bacteria. At 6 h post-infection (for HeLa, Caco2, HepG2, JEG-3 and hCMEC/D3 cells) or 18 h post-infection (for BMDM), cells were fixed and counterstained with Hoechst 33342 (Thermo Scientific, Cat.# 62249) to label nuclei and imaging was performed on a Nikon Ti2 W1 Yokogawa spinning disk confocal microscope equipped with a Plan Apo λ 20x/0.8 DIC I objective. The spreading area of infection foci was quantified by manually delineating individual foci in ImageJ based on the fluorescent signal, followed by measurement of the selected area.

### Gentamicin protection assay

Prior to infection, THP-1 cells were differentiated in RPMI supplemented with 10% FBS and 50 ng/mL PMA for three days. 2 × 10^5^ differentiated THP-1 cells were seeded in 24-well tissue culture plates and cultured in RPMI with 10% FBS for 18–24 h. After two washes with PBS, bacteria grown in BHI for 16 h were added to the cells at a MOI of 50:1 in cell culture medium and incubated for 1 h at 37 °C. Cells were then washed three times with PBS, and extracellular bacteria were killed by incubating infected cells for 1 h in cell culture medium containing 30 μg/mL gentamicin. At 2 h post-infection, the cells were washed twice with PBS and lysed with PBS supplemented with 1% Triton X-100 (Millipore Sigma, Cat.# 9002-93-1). Serial dilutions of the lysates were plated on LB agar plates to enumerate viable intracellular bacteria.

### Intracellular growth assay

HeLa, Caco2, HepG2, JEG-3, hCMEC/D3 cells (2 × 10^5^ cells/well), and BMDM (2 × 10^6^ cells/well) were seeded into 24-well plates 18–24 h prior to infection. *Lm* 10403S or *Lm* 10403SΔ*actA* were grown for 16 h in 3 mL of BHI medium at 30 °C without shaking. The bacterial cultures were washed twice with PBS and used to infect host cells (MOI of 50 for HeLa cells; MOI of 10 for HepG2 cells; MOI of 2 for Caco2 cells; MOI of 0.5 for JEG-3 cells; MOI of 10 for hCMEC/D3 cells; MOI of 0.001 for BMDM). At 1 h post-infection, infected cells were washed three times with PBS, and fresh cell culture medium containing 30 μg/mL gentamicin was added to kill extracellular bacteria. At the indicated time points, bacteria were collected by lysing host cells in 200 μL of PBS containing 1% Triton X-100. Serial dilutions of the lysates were then plated on LB agar plates and incubated 24–36 h at 37 °C to determine bacterial colony-forming units (CFU).

### Histology and immunofluorescence of liver tissue

Liver samples obtained from WT(E), WT(M), *Abi1-EKO* and *Abi1-MKO* mice infected with *Lm* 10403S for 72 hours were fixed with 4% PFA 16–20 h at 4 °C. The tissues were cryoprotected in 30% sucrose-PBS (w/v) 16–20 h at 4 °C, embedded in O.C.T. compound (Tissue-Tek, Cat.# 4583), and snap-frozen in pre-chilled 2-methylbutane (Millipore Sigma, Cat.# M32631). Sections of 5 μm thickness were prepared using a Cryostat (Leica CM 1950) and mounted onto Superfrost Plus^TM^ slides (VWR, Cat.# 48311-703).

For hematoxylin and eosin (H&E) staining, slides were rehydrated and sequentially stained: Hematoxylin (VWR, Cat.# 41810-472) (3 minutes), H_2_O (1 minute), Clarifier (VWR, Cat.# 41810-468) (1 minute), H_2_O (1 minute), Bluing Reagent (VWR, Cat.# 89237-216) (3 minutes), H_2_O (1 minute), 95% EtOH (rinse), Eosin (VWR, Cat.# 41810-450) (30 seconds), 75% EtOH (rinse), 95% EtOH (rinse), 100% EtOH (1 minute × 3), Xylene (Millipore Sigma, Cat.# XX0060-4) (1 minute × 3). Tissue sections were sealed with Permount mounting media (Electron Microscopy Sciences, Cat.# 17986-01). Liver infection foci were imaged with an Olympus VS200 Slide Scanner using a 20x objective. Number of foci were quantified per square millimeter of tissue.

For immunofluorescence staining, sections were blocked in a solution of 1% BSA (AMRESCO, Cat.# 0332-100 G), 22.5 mg/mL glycine (Millipore Sigma, Cat.# G7126-1KG), 0.1% Tween 20 in PBS for 30 min at room temperature. Tissue was stained with antibody to *Lm* (BD, Cat.# 223021) 16–20 h at 4 °C, washed with PBS, and counterstained with a secondary antibody (Goat anti-rabbit AlexaFluor 488^TM^, Cat.# A32731) and Hoechst 33342. Imaging was performed on a Nikon Ti2 W1 Yokogawa spinning disk confocal microscope equipped with a Plan Apo λ 20x/0.8 DIC I objective. The spreading area of liver infection foci was quantified by manually delineating individual foci in Fiji based on the fluorescent signal, followed by measurement of the selected area.

### SDS-PAGE and Immunoblotting

For cell lysates, 2 × 10^5^ cells were seeded per well in a 24-well plate. Cells were washed with ice-cold PBS and lysed on ice for 10 min in 200 μL RIPA buffer (25 mM Tris-HCl pH 7.4, 150 mM NaCl, 1% NP-40, 0.5% sodium deoxycholate, 0.1% SDS) supplemented with protease and phosphatase inhibitors (Millipore Sigma, Cat.# 04693159001, 4906845001). Lysates were cleared by centrifugation at 21,000 × *g* for 10 min at 4 °C. Cleared samples were mixed with 4X SDS sample buffer (LI-COR Bio, Cat.# 928-40004) supplemented with 5% β-mercaptoethanol (Bio-Rad, Cat.# 161-0710), denatured at 95 °C for 10 minutes and resolved by SDS-PAGE.

For tissue lysates, mouse tissue samples were dissected, ground with a mini pestle (SP Bel-Art, Cat.# 650009002) and lysed in T-PER^TM^ Tissue Protein Extraction Reagent (Thermo Scientific™, Cat.# 78510) on ice for 10 min. Lysates were cleared by centrifugation at 21,000 × *g* for 10 min at 4 °C. Cleared samples were mixed with 4X SDS sample buffer (LI-COR Bio, Cat.# 928-40004) supplemented with 5% β-mercaptoethanol, denatured at 95 °C for 10 min and resolved by SDS-PAGE.

Following SDS-PAGE, proteins were transferred onto nitrocellulose membranes (BIO-RAD, Cat.# 1620112) using semi-dry transfer on a Power Blotter station (Invitrogen™, Cat.# PB0010). Membranes were blocked with Intercept^®^ Blocking Buffer (LI-COR Bio, Cat.# 927-60001) for 1 h at room temperature. Primary antibody incubation was performed at 4 °C 16–20 h using antibodies diluted in TrueBlack^®^ WB Antibody Diluent (BIOTIUM, Cat.# 23013B-1L). Blots were washed with TBS-T and incubated with IRDye^®^ secondary antibodies (LI-COR Bio) at a 1:10,000 dilution. Membranes were washed again and imaged using LI-COR Image Studio (v4.0) on the Odyssey® DLx Imaging System (LI-COR Bio). Antibodies used in this study can be found in Supplementary Data 2.

### Co-Immunoprecipitation (Co-IP)

Co-immunoprecipitation experiments were performed using 2 × 10^6^ HEK293T cells, which were seeded in a 60 mm dish one day prior to transfection. Cells were transfected with the required plasmids using Lipofectamine 3000 (Invitrogen™, Cat.# L3000008). Forty-eight hours post-transfection, whole-cell lysates were prepared by adding 600 μL of lysis buffer (25 mM Tris-HCl pH 7.4, 150 mM NaCl, 5% Glycerol, 1 mM EDTA and 1% NP-40) supplemented with protease and phosphatase inhibitors (Millipore Sigma, Cat.# 04693159001, 4906845001). The lysates were rotated for 30 min at 4 °C and cleared by centrifugation at 21,000 × *g* for 10 min at 4 °C. Supernatants were collected, with 40 μL saved as sample inputs. The remaining supernatants were incubated with Anti-FLAG^®^ M2 Magnetic Beads (used for FLAG-EPS8, FLAG-EPS8ΔA, FLAG-EPS8L2; Millipore Sigma, Cat.# M8823) or Pierce™ Anti-HA Magnetic Beads (used for HA-ABI1, HA-ABI1ΔPP; Thermo Scientific™, Cat.# 88836). Immunoprecipitation was performed by incubating the lysates with beads at 4 °C for 2 hours on a tube rotator. The beads containing protein complexes were collected using a magnetic rack (Thermo Scientific^TM^, Cat.# 12321D), washed four times with lysis buffer, and the bound proteins were eluted by adding 1X SDS sample buffer (LI-COR Bio, Cat.# 928-40004), followed by boiling at 95 °C for 10 min. Both input and elution fractions were collected during immunoprecipitations and analyzed by immunoblotting.

### Quantitative immunoprecipitation and mass spectrometry

2 × 10^7^ HeLa WT and *HA-ABI1* cells were seeded in DMEM supplemented with 10% FBS in 150 mm tissue culture dishes and grown 16–20 h at 37 °C in 5% CO_2_. Cells were infected with *Lm* 10403S or *Lm* 10403SΔ*actA* at an MOI of 100:1. At 1 h post-infection, cells were washed three times with PBS, and cell culture medium containing 30 μg/mL gentamicin was added to kill extracellular bacteria. At 6 h post-infection, cells were washed twice with PBS, and protein complexes were crosslinked using 0.1 mM dithiobis (succinimidyl propionate) (DSP) (Thermo Scientific, Cat.# A35393) in PBS at 37 °C for 30 min. Crosslinking reactions were quenched by adding 20 mM Tris-HCl in PBS (pH 7.4) for 15 minutes at room temperature. Cells were lysed in 1 mL lysis buffer (25 mM Tris-HCl pH 7.4, 150 mM NaCl, 5% Glycerol, 1 mM EDTA and 1% NP-40) with protease and phosphatase inhibitors (Millipore Sigma, Cat.# 04693159001, 4906845001) for 30 min at 4 °C with gentle rotation. Lysates were cleared by centrifugation at 21,000 × *g* for 10 min at 4 °C, and 40 μL of supernatants saved as input samples. The remaining supernatants were supplemented with Pierce™ Anti-HA Magnetic Beads (Thermo Scientific™, Cat.# 88836) at 4 °C for 2 h on a tube rotator to immunoprecipitate HA-tagged ABI1 protein complexes. Beads were collected using a magnetic rack, washed four times with lysis buffer, and the bound proteins were eluted using elution buffer (50 mM Tris-HCl pH 7.5, 10% SDS) by boiling at 95 °C for 10 min.

For on-bead digestion, protein samples from immunoprecipitations were washed three times with 200 mM EPPS (pH 8.5) (Thermo Scientific™, Cat.# J61476.AK) and digested with trypsin in the same buffer for 6 h at 37 °C. Digested supernatants were separated, and beads were washed with 200 mM EPPS, with wash fractions combined with supernatants. The resulting digested peptides were labeled with TMTPro 18plex reagents (Thermo Scientific™, Cat.# A52045), and a 2 μL aliquot of each sample was pooled and used for a ratio check to confirm complete TMT labeling. All 18 TMTPro-labeled samples were pooled in ratios determined from the ratio check and desalted using a StageTips (Thermo Scientific^TM^, Cat.# 87782) before analysis on an Orbitrap Eclipse mass spectrometer.

MS2 spectra were searched using the COMET algorithm against a composite Human and *Listeria* Uniprot database, which included reversed complement and known contaminants. Peptide spectral matches were filtered to a 1% false discovery rate (FDR) using the target-decoy strategy and linear discriminant analysis. Protein-level quantifications were derived from peptides meeting strict criteria, including a summed signal-to-noise threshold of >180 and isolation specificity >0.5, with proteins further filtered to a < 1% FDR.

### RNA interference (RNAi)

Dried esiRNA oligos were resuspended in TE buffer (10 mM Tris-HCl pH 8.0, 1 mM EDTA). esiRNA targeting GFP was used as a negative control for all esiRNA experiments. HeLa cells were transfected 48 hours prior to infection using Lipofectamine RNAiMax (Invitrogen™, Cat.# 13778075), following the manufacturer’s instructions. Transfections were performed at a final concentration of 10 pmol. The knockdown efficiency of target genes was confirmed by immunoblot analysis.

### RT-qPCR

RNA from HeLa cells was isolated using the TRIzol™ Reagent (Invitrogen™, Cat.# 15596018) following the manufacturer’s protocol. cDNA was synthesized from the isolated RNA using SuperScript™ IV Reverse Transcriptase (Invitrogen™, Cat.# 18090010). For quantitative PCR, 10 ng of cDNA per reaction was used, along with iTaq Universal SYBR Green Supermix (Bio-Rad, Cat.# 1725120). GAPDH served as the housekeeping gene for normalization. All primers used in the study are listed in Supplementary Data 2.

### Live-cell imaging analysis

For live-cell imaging, 5 × 10^5^ cells were seeded in a 6-well glass-bottom plate (Cellvis, Cat.# P06-1.5H-N) one day prior to transfection. The following day, cells were transfected with required plasmids using Lipofectamine 3000. Twenty-four hours post-transfection, cells were infected with fluorescent *Lm* at a MOI of 100. At 1 h post-infection, cells were washed with PBS and the culture media was replaced with live-cell imaging media (FluoroBrite™ DMEM supplemented with 10% FBS and 1X ProLong™ Live Antifade Reagent) containing 30 μg/mL gentamicin. In some experiments, F-actin was stained with SiR-actin kit (Cytoskeleton, Cat.# CY-SC001) at 200 nM concentration in live-cell imaging media. Imaging was performed at 5–7 h post-infection using a Nikon Ti2 W1 Yokogawa spinning disk confocal microscope equipped with a Plan Apo λD 60x/1.42 Oil DIC objective. Images were acquired at 37 ^o^C at 15 s intervals for 20–30 min.

Bacterial movement speed (with actin comet tail) or protrusion elongation speed was calculated using the TrackMate plugin in Fiji software. Individual bacteria in focus were tracked over their observable course, and the speed was calculated by measuring the distance traveled over the recorded time interval.

For monitoring ABI1 dynamics at the plasma membrane (PM), 3 × 10^5^ HeLa WT cells expressing mNeonGreen-ABI1 and mCherry-EPS8 were seeded in a 35 mm glass bottom dish (ibidi, Cat.# 81218-200) in DMEM with 10% FBS and incubated 16–20 h at 37 °C with 5% CO_2_. Cells were subsequently infected with the indicated *Lm* strains (MOI 200:1) or treated with drugs ionomycin (STEMCELL, Cat.# 73724) (5 μM), EGTA (Millipore Sigma, Cat.# 324626) (1 mM) or recombinant LLO (0.5 nM) in live-cell imaging media (FluoroBrite™ DMEM supplemented with 10% FBS and 1X ProLong™ Live Antifade Reagent). To visualize ABI1 protein dynamics beneath the PM, critical-angle TIRF microscopy (penetration depth ~80 nm) was performed using a Nikon Ti2E Motorized Inverted Microscope equipped with Ring-TIRF illumination and an Apo TIRF 100x/1.49 DIC N2 objective. Widefield (EPI) images were also acquired under identical settings as controls.

### Immunofluorescence microscopy

For imaging, 2 × 10^5^ HeLa cells were seeded on coverslips in 24-well plates and infected with *Lm* 10403S expressing TagBFP for 1 h at 37 °C. At 1 h post-infection, cells were washed three times with PBS, and cell culture medium containing 30 μg/mL gentamicin was added. At 6 h post-infection, cells were washed three times with PBS and fixed with 4% PFA for 30 min at room temperature. After fixation, cells were washed twice with PBS and rinsed briefly in quenching solution (QS; 0.1 M Glycine in PBS pH 7.4) followed by incubation in QS for 10 min at room temperature on an orbital shaker. The cells were then washed once with PBS and blocking and permeabilization buffer (BPB; 10% Normal goat serum, 0.1% Triton X-100 in PBS) was added for 30 min. Primary antibodies (Rabbit anti-ABI1 (Proteintech, Cat.# 27387-1-AP), and Mouse anti-EZR (Invitrogen, Cat.# 35-7300)) diluted in blocking solution (BS; 5% normal goat serum in PBS) were then added, and cells were incubated at 4 °C 16–20 h. Secondary antibodies and dyes (FluoTag^®^-X4 anti-Rabbit IgG-Atto488 (NanoTag Biotechnologies, Cat.# N2404-At488-S), FluoTag^®^-X2 anti-Mouse IgG1-Alexa647 (NanoTag Biotechnologies, Cat.# N2002-AF647-S), Hoechst 33342, and Texas Red™-X Phalloidin (Invitrogen, Cat.# T7471)) were diluted in FluoTag dilution buffer (3% Normal goat serum, 0.1% Triton X-100 in PBS) and added to the cells for 1 h at room temperature. Coverslips were removed and extensively washed with PBS, once with ddH_2_O and mounted using 5 μL ProLong™ Glass Antifade Mountant (Invitrogen™, Cat.# P36984).

Imaging was performed using a Zeiss LSM 980 point-scanning confocal microscope with Zeiss Zen Blue software. The system includes incident light fluorescence and laser illumination (405, 458, 488, 514, 561, 594, 639 and 730 nm), a GaAsP 32-channel spectral detector, two multi-alkali PMT detectors and an Airyscan2 Detector for enhanced sensitivity and resolution. A Plan Apochromat 63x/1.4 oil immersion objective (Zeiss Instruments) was used for imaging. To compare ABI1 recruitment into *Lm*-containing protrusions across different bacterial mutants, images were acquired using 2x NIR and Airyscan2 super-resolution detectors (provides a 1.7x improvement in spatial resolution beyond the theoretical diffraction limit), followed by image reconstruction in Zen Blue Acquisition Software. All raw microscopy data were processed using Fiji to generate representative images.

### Photo-conversion and local actin recycling tracking

5 × 10^5^ HeLa WT or *ABI1* KO cells expressing mEOS3.2-Actin were seeded in a 35 mm glass bottom dish (ibidi, Cat.# 81218-200) in DMEM with 10% FBS and incubated 16–20 h at 37 °C with 5% CO_2_. Cells were infected with *Lm* 10403S expressing TagBFP for 1 hour at 37 °C. At 1 h post-infection, cells were washed three times with PBS and live-cell imaging media (FluoroBrite™ DMEM supplemented with 10% FBS and 1X ProLong™ Live Antifade Reagent) containing 30 μg/mL gentamicin was added. Imaging was performed at 5–7 h post-infection using a Zeiss LSM 980 point-scanning confocal microscope. Images were taken using a Plan Apochromat 63x/1.4 oil immersion objective (Zeiss Instruments). Regions of interest (ROI), approximately 2–3 μm from a bacterial pole within protrusions, were selected using interactive photobleaching tools. The ROI surface area was kept constant across experiments. ROIs were exposed to 100% 405 laser power for ~ 1 s to excite the mEOS3.2. Time-lapse images were captured at maximum speed (~ 0.24 s intervals) for 1 min, recording the photo-converted mEOS3.2 (red) and unconverted mEOS3.2 (green) signals simultaneously to track actin local recycling.

Image analysis was performed using Fiji software. To quantify actin local recycling, raw images were enhanced with Gaussian blur filters to improve contrast. Single-particle tracking was conducted using the Fiji Trackmate plugin. The proportion of recycled actin (proximal red signal) was calculated from the tracked data accordingly. The quantification of photo-converted mEOS3.2-Actin was initiated from the first frame acquired immediately after photo-conversion.

### In vivo virulence studies and deduced LD_50_ determination

For *Lm* mice infections, sex- and age-matched mice were intravenously injected with the following bacterial doses: 5 × 10^4^ CFU/mouse of 10403S, 1 × 10^4^ CFU/mouse of LS743, or 1 × 10^7^ CFU/mouse of 10403SΔ*actA* and LS743Δ*actA* strains. For oral infections, mice were inoculated by orogastric administration with 2 × 10^9^ bacteria of LS743 using plastic feeding tubes (Instech, Cat.# FTP-20-30). At 72 h post-infection, mice were euthanized via CO_2_ exposure followed by cervical dislocation, and organs were collected for bacterial CFU enumeration.

For survival studies, mice were intravenously infected with the indicated amount of CFU of *Lm* 10403S. Animals were monitored twice daily for signs of disease, and moribund mice were humanely euthanized. The percent survival was plotted using the Kaplen-Meier function over 10 days post-infection. The deduced 50% lethal dose (LD_50_) was estimated based on survival outcomes.

All animal care and infection experiments were conducted at the Harvard Institutes of Medicine BL-2 Animal Facility in compliance with the Association for Assessment and Accreditation of Laboratory Animal Care International regulations. All experimental protocols (IACUC; protocol no. IS-065-9) were approved by the Harvard Medical School Institutional Animal Care and Use Committee (IACUC) and conduced in compliance with federal, state and local laws.

### Statistics and reproducibility

For all experiments, data are presented as mean ± SD, unless otherwise specified in the figure legends. Statistical analyses and data visualization were performed using GraphPad Prism (v9.1.1), Microsoft Excel (v16.106), and R (v4.4.2). Gene Ontology (GO) enrichment analysis was performed using the enrichGO function in the R package clusterProfiler (v4.14.6). Details of the statistical tests used are provided in the corresponding figure legends. A *P* value < 0.05 was considered statistically significant. Exact *P* values are reported in the figures where available; for comparisons yielding *P* < 0.0001, values are reported as <0.0001; for comparisons yielding *P* > 0.9999, values are reported as > 0.9999. All statistical analyses were performed using two-sided tests unless otherwise specified in the figure legends. All experiments were independently repeated at least three times with biological replicates. Mass spectrometry (MS) was performed once and identified candidate proteins were subsequently validated by co-immunoprecipitation (Co-IP).

Sample sizes were not predetermined by statistical methods. Randomization was not applied, as experiments were conducted based on predefined genotypes or experimental conditions. Investigators were not blinded to group allocation; however, control and experimental samples were processed in parallel and handled identically to ensure consistency.

### Reporting summary

Further information on research design is available in the Nature Portfolio Reporting Summary linked to this article.

## Supplementary information

Supplementary Information File

Description of Additional Supplementary Files

Supplementary Data 1

Supplementary Data 2

Supplementary Data 3

Supplementary Movie 1

Supplementary Movie 2

Supplementary Movie 3

Supplementary Movie 4

Supplementary Movie 5

Supplementary Movie 6

Reporting Summary

Transparent Peer Review file

## Source data

Source Data

## Data Availability

All data supporting the findings of this study are available within the Article and its Supplementary Information. Mass spectrometry data used for analysis can be found in Supplementary Data 3. The protein mass spectrometry raw data generated in this study have been deposited in the ProteomeXchange Consortium via the PRIDE partner repository under accession code PXD074581. Bacterial strains, plasmids, cell lines, and mouse lines will be made available from the authors upon request. Further information and requests for resources and reagents should be directed to the lead contact, Dr. Darren Higgins (darren_higgins@hms.harvard.edu). Source data are provided with this paper.

## Code availability

No software or code was generated for this study.
