# Supplementary material for: A pore-forming toxin initiates ABI1 complex switching to promote bacterial cell-to-cell spread
Source: Nat Commun. 2026 Apr 13;17:5129. doi: 10.1038/s41467-026-71510-z (PMC13247217; doi:10.1038/s41467-026-71510-z)
Supplement: Supplementary file 1 — Supplementary Information File [file 41467_2026_71510_MOESM1_ESM.pdf]

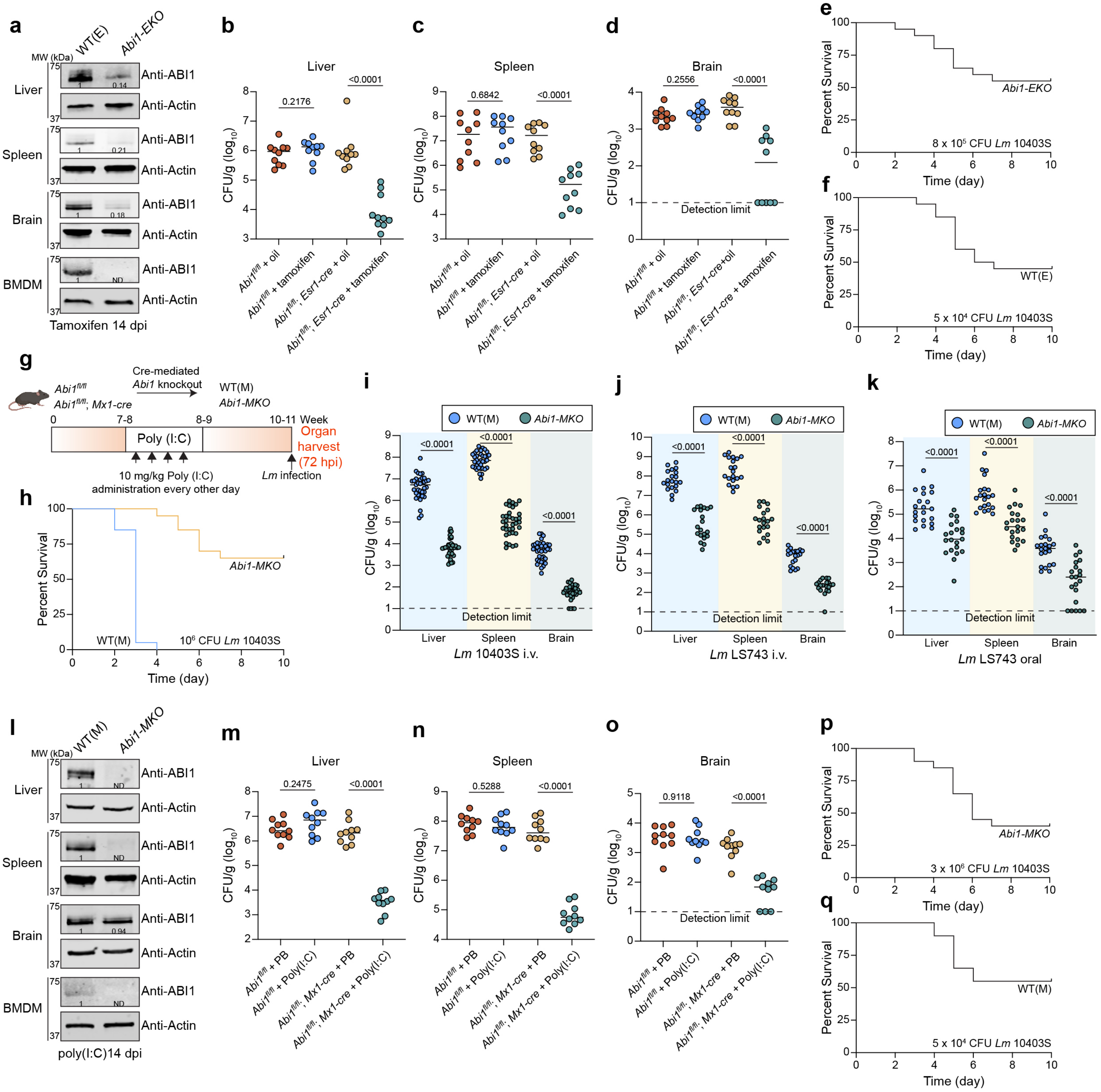

a
WT(E)
Abi1-EKO
MW (kDa)
Liver
Spleen
Brain
BMDM
Anti-ABI1
Anti-Actin
ND
Tamoxifen 14 dpi
b
Liver
c
Spleen
d
Brain
CFU/g (log10)
Detection limit
Abi1fl/fl + oil
Abi1fl/fl + tamoxifen
Abi1fl/fl; Esr1-cre + oil
Abi1fl/fl; Esr1-cre + tamoxifen
0.2176
<0.0001
0.6842
0.2556
e
Percent Survival
Abi1-EKO
8 x 10⁵ CFU Lm 10403S
Time (day)
f
WT(E)
5 x 10⁴ CFU Lm 10403S
g
Abi1fl/fl
Abi1fl/fl; Mx1-cre
Cre-mediated Abi1 knockout
WT(M)
Abi1-MKO
Week
Poly (I:C)
Organ harvest (72 hpi)
10 mg/kg Poly (I:C) administration every other day
Lm infection
h
10⁶ CFU Lm 10403S
i
Lm 10403S i.v.
j
Lm LS743 i.v.
k
Lm LS743 oral
l
WT(M)
Abi1-MKO
poly(I:C)14 dpi
m
n
o
Abi1fl/fl + PB
Abi1fl/fl + Poly(I:C)
Abi1fl/fl; Mx1-cre + PB
Abi1fl/fl; Mx1-cre + Poly(I:C)
0.2475
0.5288
0.9118
p
3 x 10⁶ CFU Lm 10403S
q
5 x 10⁴ CFU Lm 10403S

Supplementary Fig 1

a, Western blot analysis using an ABI1 antibody was performed to detect the inducible knockout of ABI1 expression in the liver, spleen, brain, and bone marrow-derived macrophages (BMDM) isolated from WT(E) and *Abi1-EKO* mice. Detection of β-Actin was used as a loading control. ABI1 band intensities of liver, spleen, brain and BMDM samples are shown below the respective bands. ABI1 band intensities were normalized to actin with the WT(E) sample set to 1. ND = not detected.

b-d, Bacterial burden in the liver (**b**), spleen (**c**) and brain (**d**) of $Abi1^{fl/fl}$ and $Abi1^{fl/fl}$; *Esr1-cre* mice induced with either tamoxifen or corn oil (oil). All mice were infected with 5 x 10^4 CFU *Lm* 10403S via intravenous injection, and organs were harvested at 72 hpi. N = 10 mice per group.

e, Kaplan-Meier survival curve showing the 10-day percent survival of *Abi1-EKO* mice infected with 8 x 10^5 CFU *Lm* 10403S via intravenous injection. N = 20 mice.

f, Kaplan-Meier survival curve showing the 10-day percent survival of WT(E) mice infected with 5 x 10^4 CFU *Lm* 10403S via intravenous injection. N = 20 mice.

g, Schematic representation of the experimental setup for $Abi1^{fl/fl}$ and $Abi1^{fl/fl}$; *Mx1-cre* mice induction and *Lm* infection. Seven to eight-week-old $Abi1^{fl/fl}$ or $Abi1^{fl/fl}$; *Mx1-cre* mice were injected intraperitoneally with 10 mg/kg Poly(I:C) every other day for four doses. $Abi1^{fl/fl}$ or $Abi1^{fl/fl}$; *Mx1-cre* mice that were treated with Poly(I:C) are referred to as WT(M) and *Abi1-MKO*, respectively. Fourteen days post-induction, mice were infected with *Lm* as indicated. Target organs were harvested at 72 hours post-infection for bacterial burden analysis.

h, Kaplan-Meier survival curve showing the 10-day percent survival of WT(M) and *Abi1-MKO* mice infected with 1 x 10^6 CFU *Lm* 10403S via intravenous injection. N = 20 mice per group.

i, Bacterial burden in the liver, spleen and brain. WT(M) and *Abi1-MKO* mice were infected with 5 x 10^4 CFU *Lm* 10403S via intravenous injection. At 72 hours post-infection, the liver, spleen, and brain of each mouse was collected and the bacterial burden determined. N = 35 mice per group.

j, Bacterial burden in the liver, spleen and brain. WT(M) and *Abi1-MKO* mice were infected with 1 x 10^4 CFU *Lm* LS743 via intravenous injection. At 72 hours post-infection, the liver, spleen, and brain of each mouse was collected and the bacterial burden determined. N = 20 mice per group.

k, Bacterial burden in the liver, spleen and brain. WT(M) and *Abi1-MKO* mice were infected with 2 x 10^9 CFU *Lm* LS743 via oral gavage. At 72 hours post-infection, the liver, spleen, and brain of each mouse was collected and the bacterial burden determined. N = 21 mice per group.

l, Western blot analysis using an ABI1 antibody was performed to detect the inducible knockout of ABI1 expression in the liver, spleen, brain, and BMDM isolated from WT(M) and *Abi1-MKO* mice. Detection of β-Actin was used as a loading control. ABI1 band intensities of liver, spleen, brain and BMDM samples are shown below the respective bands. ABI1 band intensities were normalized to actin with the WT(M) sample set to 1. ND = not detected.

m-o, Bacterial burden in the liver (**m**), spleen (**n**) and brain (**o**) of $Abi1^{fl/fl}$ and $Abi1^{fl/fl}$*; Mx1-cre* mice induced with either Poly(I:C) or physiological buffer (0.9% NaCl, PB). All mice were infected with 5 x 10^4 CFU of *Lm* 10403S via intravenous injection, and organs were harvested at 72 hpi. N = 10 mice per group.

p, Kaplan-Meier survival curve showing the 10-day percent survival of *Abi1-MKO* mice infected with 3 x 10^6 CFU of *Lm* 10403S via intravenous injection. N = 20 mice per group.

q, Kaplan-Meier survival curve showing the 10-day percent survival of WT(M) mice infected with 5 x 10^4 CFU of *Lm* 10403S via intravenous injection. N = 20 mice per group.

Data shown are medians (**b**-**d**, **i-k**, **m-o**). The dashed line indicates a limit of detection of 10 CFU (**d**, **i-k**, **o**). The Mann-Whitney *U* test (two-sided) was used to assess statistical significance (**b**-**d**, **i**-**k**, **m**-**o**). Exact *P* values are shown in the figures. The diagram in **g** was created in BioRender. Sun, H. (2026) https://BioRender.com/wwbb7ml. Source data are provided as a Source Data file.

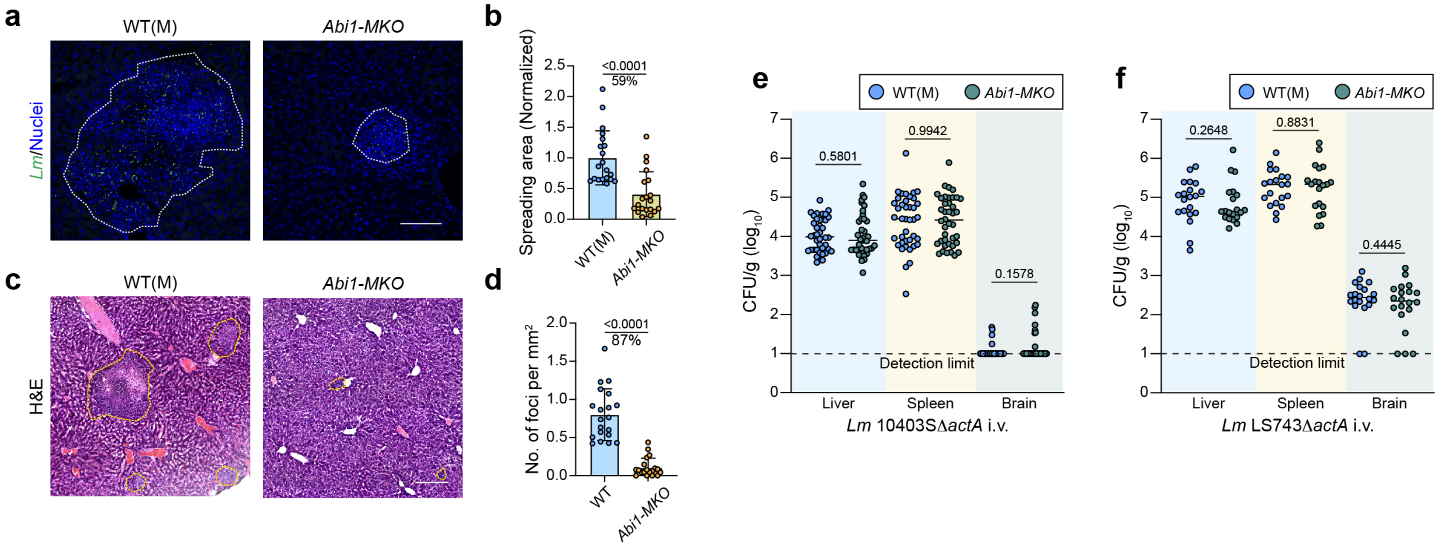

a
WT(M)
Abi1-MKO
Lm/Nuclei
b
Spreading area (Normalized)
<0.0001
59%
WT(M)
Abi1-MKO
c
WT(M)
Abi1-MKO
H&E
d
No. of foci per mm²
<0.0001
87%
WT
Abi1-MKO
e
WT(M)
Abi1-MKO
CFU/g (log10)
0.5801
0.9942
0.1578
Detection limit
Liver
Spleen
Brain
Lm 10403SΔactA i.v.
f
WT(M)
Abi1-MKO
CFU/g (log10)
0.2648
0.8831
0.4445
Detection limit
Liver
Spleen
Brain
Lm LS743ΔactA i.v.

Supplementary Fig 2

a, Representative images of infection foci in the liver of WT(M) and *Abi1-MKO* mice at 72 hpi. Tissues were stained with *Lm* antibody and Hoechst 33342 (nuclei). Dotted lines delineate the edge of the infection foci. Scale bar = 100 μm.
b, Quantification of the spreading area of infection foci. The mean spreading area of WT(M) infection foci from (a) was calculated and used as the reference value. N = 20 foci quantified from liver sections derived from five mice per group. Data are presented as mean ± SD with all data points shown and the percent reduction in spreading area is indicated.
c, Representative images of infection foci in the liver of WT(M) and *Abi1-MKO* mice at 72 hpi. Tissues were stained with H&E. Dotted lines delineate the edge of the infection foci.
d, Quantification of the number of infection foci. N = 20 images of liver sections derived from five mice per group. Data are presented as mean ± SD with all data points shown and the percent reduction in the number of foci is indicated.
e, Bacterial burden in the liver, spleen and brain. WT(M) and *Abi1-MKO* mice were infected with 1 x 10^7 CFU *Lm* 10403SΔ*actA* via intravenous injection. At 72 hours post-infection, the liver, spleen, and brain of each mouse was collected and the bacterial burden determined. Data are shown as medians. N = 40 mice.
f, Bacterial burden in the liver, spleen and brain. WT(M) and *Abi1-MKO* mice were infected with 1x 10^7 CFU *Lm* LS743Δ*actA* via intravenous injection. At 72 hours post-infection, the liver, spleen, and brain of each mouse was collected and the bacterial burden determined. Data are shown as medians. N = 20 mice.
The dotted line indicates a limit of detection of 10 CFU (**e**, **f**). The two-tailed unpaired student's *t*-test (equal variance) was used to assess statistical significance (**b**). The two-tailed unpaired Welch's *t*-test (unequal variance) was used to assess statistical significance (**d**). The Mann-Whitney *U* test (two-sided) was used to assess statistical significance (**e**, **f**). Exact *P* values are shown in the figures. Source data are provided as a Source Data file.

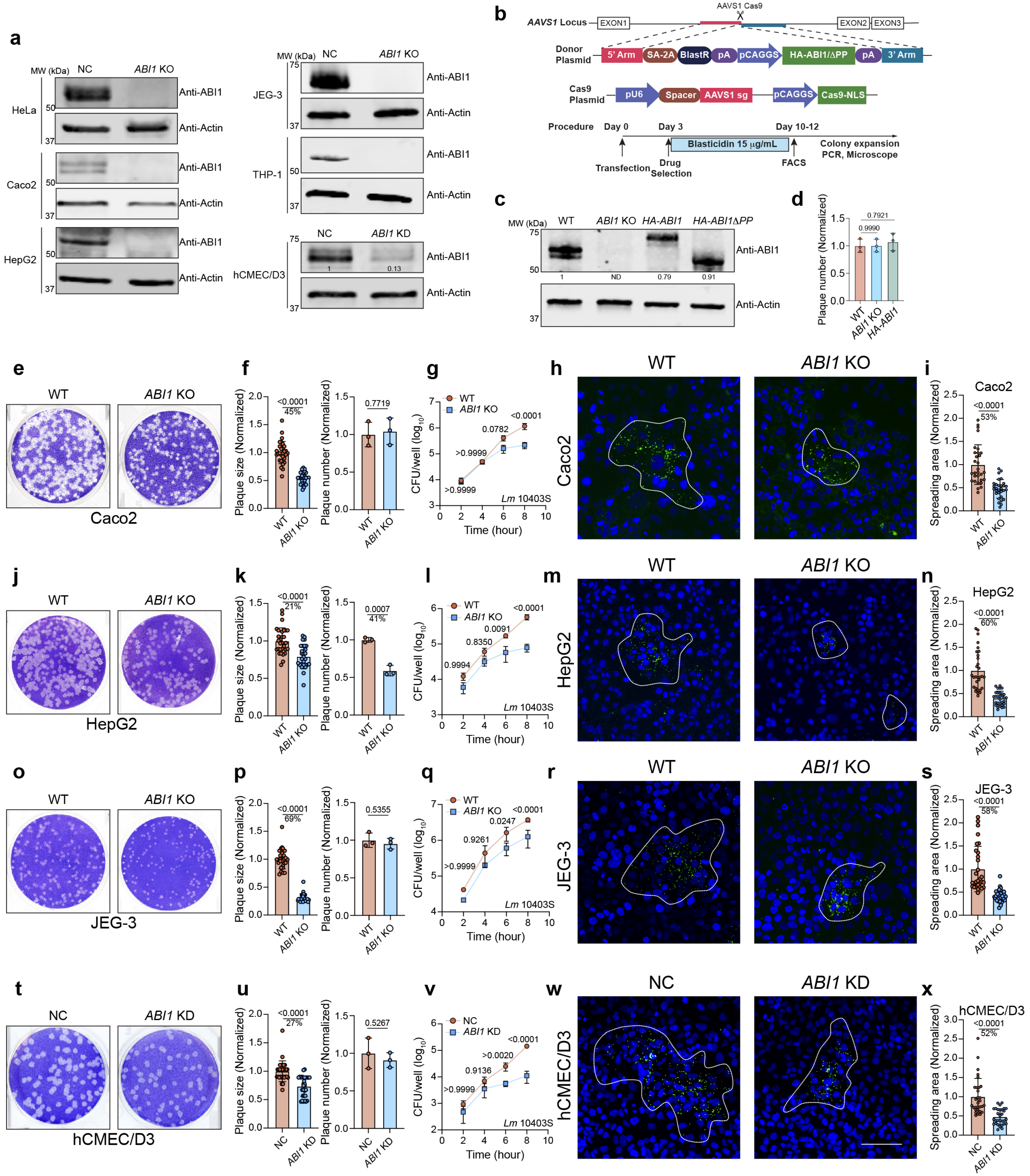
a
MW (kDa) NC ABI1 KO
HeLa
Caco2
HepG2
Anti-ABI1
Anti-Actin
JEG-3
THP-1
hCMEC/D3
NC ABI1 KD
b
AAVS1 Locus
AAVS1 Cas9
EXON1 EXON2 EXON3
Donor Plasmid
5' Arm SA-2A BlastR pA pCAGGS HA-ABI1/ΔPP pA 3' Arm
Cas9 Plasmid
pU6 Spacer AAVS1 sg pCAGGS Cas9-NLS
Procedure Day 0 Day 3 Day 10-12
Blasticidin 15 μg/mL
Transfection Drug Selection FACS
Colony expansion PCR, Microscope
c
WT ABI1 KO HA-ABI1 HA-ABI1ΔPP
d
Plaque number (Normalized)
e Caco2
f Plaque size (Normalized)
g CFU/well (log10)
Time (hour)
Lm 10403S
h WT ABI1 KO
i Spreading area (Normalized)
j HepG2
k l m n
o JEG-3
p q r s
t hCMEC/D3
u v w x

Supplementary Fig 3

a, Western blot analysis using an ABI1 antibody was performed to confirm the absence of ABI1 expression in the HeLa, Caco2, HepG2, JEG-3, and THP-1 knockout cell lines, as well as the hCMEC/D3 knockdown cell line. Detection of β-Actin was used as a loading control. ABI1 band intensities of hCMEC/D3 cells are shown below the respective bands. ABI1 band intensities were normalized to actin with the NC sample set to 1. All other KO cell lines had no detectable ABI1 band.

b, Schematic representation of the CRISPR-Cas9 strategy for the generation of complementation cell lines. Expression cassettes were integrated into the AAVS1 locus to minimize positional effects.

c, Western blot analysis using an ABI1 antibody was performed to confirm the expression of ABI1 or ABI1ΔPP in the *HA-ABI1* or *HA-ABI1ΔPP* complementation cell lines used in this study. Detection of β-Actin was used as a loading control. ABI1 band intensities are shown below the respective bands. ABI1 band intensities were normalized to actin with the WT sample set to 1. ND = not detected.

d, Quantification of plaque numbers from Figure 2**a**. N = 3 biological replicates. Data are presented as mean ± SD with all data points shown. Total plaques counted for WT, *ABI1* KO and *HA-ABI1* HeLa cells per biological replicate ranged from 129 to 175.

e, Plaque formation assay. WT and *ABI1* KO Caco2 cells were infected with *Lm* 10403S. After 1 hour, the infected monolayers were washed, and medium containing gentamicin was added. At 72-hours post-infection, monolayers were fixed and stained with crystal violet for plaque visualization.

f, Plaque size (left) and plaque number (right) measurements from (**e**). N = 30 plaques for plaque size determination. N = 3 biological replicates for plaque number quantification with total plaques counted per biological replicate ranging from 119 to 169. Data are presented as mean ± SD with all data points shown and the percent reduction in plaque size is indicated.

g, Intracellular growth in WT and *ABI1* KO Caco2 cells. Cells were infected with *Lm* 10403S. After 1 hour, the infected monolayers were washed, and medium containing gentamicin was added. At 2-hour intervals post-infection, cells were lysed, and intracellular bacteria were enumerated by plating serial dilutions of lysates. Data are presented as the mean ± SD of a representative experiment performed in triplicate and repeated three times with similar results.

h, **i**, Representative images (**h**) and quantification (**i**) of an infection focus assay measuring *Lm* 10403S cell-to-cell spread in WT and *ABI1* KO Caco2 cells. Dotted lines delineate the edge of the infection foci. Data are presented as mean ± SD with all data points shown and the percent reduction in spreading area is indicated. N = 30 foci.

j, Plaque formation assay. WT and *ABI1* KO HepG2 cells were infected with *Lm* 10403S. After 1 hour, the infected monolayers were washed, and medium containing gentamicin was added. At 72-hours post-infection, monolayers were fixed and stained with crystal violet for plaque visualization.

k, Plaque size (left) and plaque number (right) measurements from (**j**). N = 30 plaques for plaque size determination. N = 3 biological replicates for plaque number quantification with total plaques counted per biological replicate ranging from 130 to 246. Data are presented as mean ± SD with all data points shown and the percent reductions in plaque size and plaque number indicated.

l, Intracellular growth in WT and *ABI1* KO HepG2 cells. Cells were infected with *Lm* 10403S. After 1 hour, the infected monolayers were washed, and medium containing gentamicin was added. At 2-hour intervals post-infection, cells were lysed, and intracellular bacteria were enumerated by plating serial dilutions of lysates. Data are presented as the mean ± SD of one representative experiment performed in triplicate and repeated three times with similar results.

m, **n**, Representative images (**m**) and quantification (**n**) of an infection focus assay measuring *Lm* 10403S cell-to-cell spread in WT and *ABI1* KO HepG2 cells. Dotted lines delineate edge of the infection foci. Data are presented as mean ± SD with all data points shown and the percent reduction in spreading area is indicated. N = 30 foci.

o, Plaque formation assay. WT and *ABI1* KO JEG-3 cells were infected with *Lm* 10403S. After 1 hour, the infected monolayers were washed, and medium containing gentamicin was added. At 72-hours post-infection, monolayers were fixed and stained with crystal violet for plaque visualization.

p, Plaque size (left) and plaque number (right) measurements from (**o**). N = 30 plaques for plaque size determination. N = 3 biological replicates for plaque number quantification with total plaques counted per biological replicate ranging from 131 to 166. Data are presented as mean ± SD with all data points shown and the percent reduction in plaque size is indicated.

q, Intracellular growth in WT and *ABI1* KO JEG-3 cells. Cells were infected with *Lm* 10403S. After 1 hour, the infected monolayers were washed, and medium containing gentamicin was added. At 2-hour intervals post-infection,

cells were lysed, and intracellular bacteria were enumerated by plating serial dilutions of lysates. Data are presented as the mean ± SD of one representative experiment performed in triplicate and repeated three times with similar results.

r, **s**, Representative images (**r**) and quantification (**s**) of an infection focus assay measuring *Lm* 10403S cell-to-cell spread in WT and *ABI1* KO JEG-3 cells. Dotted lines delineate the edge of the infection foci. Data are presented as mean ± SD with all data points shown and the percent reduction in spreading area is indicated. N = 30 foci.

t, Plaque formation assay. NC (non-targeting shRNA control) and *ABI1* knockdown (*ABI1* KD) hCMEC/D3 cells were infected with *Lm* 10403S. After 1 hour, the infected monolayers were washed, and medium containing gentamicin was added. At 72-hours post-infection, monolayers were fixed and stained with crystal violet for plaque visualization.

u, Plaque size (left) and plaque number (right) measurements from (**t**). N = 30 plaques for plaque size determination. N = 3 biological replicates for plaque number quantification with total plaques counted per biological replicate ranging from 65 to 98. Data are presented as mean ± SD with all data points shown and the percent reduction in plaque size is indicated.

v, Intracellular growth in NC and *ABI1* KD hCMEC/D3 cells. Cells were infected with *Lm* 10403S. After 1 hour, the infected monolayers were washed, and medium containing gentamicin was added. At 2-hour intervals post-infection, cells were lysed, and intracellular bacteria were enumerated by plating serial dilutions of lysates. Data are presented as the mean ± SD of one representative experiment performed in triplicate and repeated three times with similar results.

w, **x**, Representative images (**w**) and quantification (**x**) of an infection focus assay measuring *Lm* 10403S cell-to-cell spread in NC and *ABI1* KD hCMEC/D3 cells. Dotted lines delineate the edge of the infection foci. Data are presented as mean ± SD with all data points shown and the percent reduction in spreading area is indicated. N = 30 foci.

The two-tailed unpaired Welch's *t*-test (unequal variance) was used to assess statistical significance (**f** (plaque size), **i**, **n**, **p** (plaque size), **s**, **x**). The two-tailed unpaired student's *t*-test (equal variance) was used to assess statistical significance (**d**, **f** (plaque number), **k**, **p** (plaque number), **u**). The repeated-measures two-way ANOVA model analysis followed by Šidák's post-hoc test was performed to assess statistical significance (**g**, **l**, **q**, **v**). Scale bar = 100 μm. Exact *P* values are shown in the figures. Source data are provided as a Source Data file.

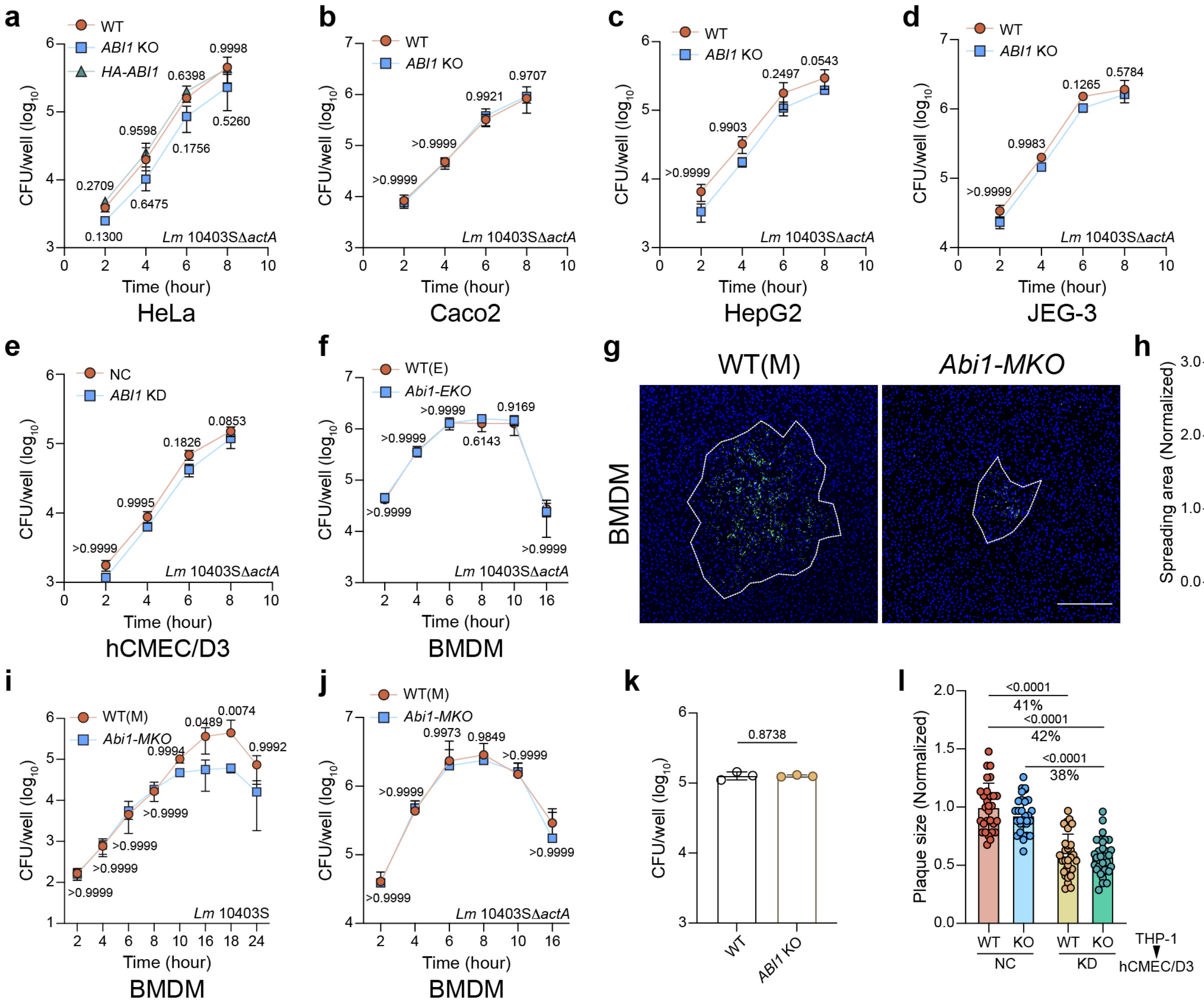

a
WT
ABI1 KO
HA-ABI1
CFU/well (log10)
Time (hour)
Lm 10403SΔactA
HeLa
b
Caco2
c
HepG2
d
JEG-3
e
NC
ABI1 KD
hCMEC/D3
f
WT(E)
Abi1-EKO
BMDM
g
WT(M)
Abi1-MKO
BMDM
h
Spreading area (Normalized)
<0.0001
79%
i
WT(M)
Abi1-MKO
Lm 10403S
BMDM
j
WT(M)
Abi1-MKO
BMDM
k
0.8738
WT
ABI1 KO
l
Plaque size (Normalized)
<0.0001
41%
<0.0001
42%
<0.0001
38%
WT
KO
WT
KO
NC
KD
THP-1
hCMEC/D3

Supplementary Fig 4

a, Intracellular growth in WT, *ABI1* KO, and *HA-ABI1* HeLa cells. Cells were infected with *Lm* 10403SΔ*actA*. After 1 hour, the infected monolayers were washed, and medium containing gentamicin was added. At 2-hour intervals post-infection, cells were lysed, and intracellular bacteria were enumerated by plating serial dilutions of lysates. Data are presented as the mean ± SD of one representative experiment performed in triplicate and repeated three times with similar results.
b, Intracellular growth in WT and *ABI1* KO Caco2 cells. Cells were infected with *Lm* 10403SΔ*actA*. After 1 hour, the infected monolayers were washed, and medium containing gentamicin was added. At 2-hour intervals post-infection, cells were lysed, and intracellular bacteria were enumerated by plating serial dilutions of lysates. Data are presented as the mean ± SD of one representative experiment performed in triplicate and repeated three times with similar results.
c, Intracellular growth in WT and *ABI1* KO HepG2 cells. Cells were infected with *Lm* 10403SΔ*actA*. After 1 hour, the infected monolayers were washed, and medium containing gentamicin was added. At 2-hour intervals post-infection, cells were lysed, and intracellular bacteria were enumerated by plating serial dilutions of lysates. Data are presented as the mean ± SD of one representative experiment performed in triplicate and repeated three times with similar results.
d, Intracellular growth in WT and *ABI1* KO JEG-3 cells. Cells were infected with *Lm* 10403SΔ*actA*. After 1 hour, the infected monolayers were washed, and medium containing gentamicin was added. At 2-hour intervals post-infection, cells were lysed, and intracellular bacteria were enumerated by plating serial dilutions of lysates. Data are presented as the mean ± SD of one representative experiment performed in triplicate and repeated three times with similar results.
e, Intracellular growth in NC and *ABI1* KD hCMEC/D3 cells. Cells were infected with *Lm* 10403SΔ*actA*. After 1 hour, the infected monolayers were washed, and medium containing gentamicin was added. At 2-hour intervals post-infection, cells were lysed, and intracellular bacteria were enumerated by plating serial dilutions of lysates. Data are presented as the mean ± SD of one representative experiment performed in triplicate and repeated three times with similar results.
f, Intracellular growth in BMDM isolated from WT(E) and *Abi1-EKO* mice. Cells were infected with *Lm* 10403SΔ*actA*. After 1 hour, the infected monolayers were washed, and medium containing gentamicin was added. Cells were lysed at the indicated time points, and intracellular bacteria were enumerated by plating serial dilutions of lysates. Data are presented as the mean ± SD of one representative experiment performed in triplicate and repeated three times with similar results.
g, **h**, Representative images (**g**) and quantification (**h**) of an infection focus assay measuring *Lm* 10403S cell-to-cell spread in BMDM isolated from WT(M) and *Abi1-MKO* mice. Dotted lines delineate the edge of the infection foci. Scale bar = 300 μm. Data are presented as mean ± SD with all data points shown and the percent reduction in spreading area is indicated. N = 20 foci.
i, Intracellular growth in BMDM isolated from WT(M) and *Abi1-MKO* mice. Cells were infected with *Lm* 10403S. After 1 hour, the infected monolayers were washed, and medium containing gentamicin was added. Cells were lysed at the indicated time points and intracellular bacteria were enumerated by plating serial dilutions of lysates. Data are presented as the mean ± SD of one representative experiment performed in triplicate and repeated three times with similar results.
j, Intracellular growth in BMDM isolated from WT(M) and *Abi1-MKO* mice. Cells were infected with *Lm* 10403SΔ*actA*. After 1 hour, the infected monolayers were washed, and medium containing gentamicin was added. Cells were lysed at the indicated time points and intracellular bacteria were enumerated by plating serial dilutions of lysates. Data are presented as the mean ± SD of one representative experiment performed in triplicate and repeated three times with similar results.
k, Gentamicin protection assay. WT and *ABI1* KO THP-1 cells were infected with *Lm* 10403S for 1 hour prior to intracellular bacteria being quantified by gentamicin protection assay at 2 hours post-infection. Data represent the mean ± SD of one representative experiment performed in triplicate and repeated three times with similar results.
l, Heterologous plaque formation assay. Plaque size measurements from Fig. 2j. N = 30 plaques. Data are presented as mean ± SD with all data points shown and the percent reduction in plaque size is indicated.
The repeated-measures two-way ANOVA model analysis followed by Šidák's post-hoc test was performed to assess statistical significance (**a**-**f**). The two-tailed unpaired Welch's *t*-test (unequal variance) was used to assess statistical significance (**h**). The repeated-measures two-way ANOVA model analysis followed by Šidák's post-hoc test was performed to assess statistical significance (**i**, **j**). The two-tailed unpaired student's *t*-test (equal variance) was used

to assess statistical significance (**k**). Two-way ANOVA (equal variance) analysis followed by Tukey's post-hoc test was performed to assess statistical significance (**l**). Exact *P* values are shown in the figures. Source data are provided as a Source Data file.

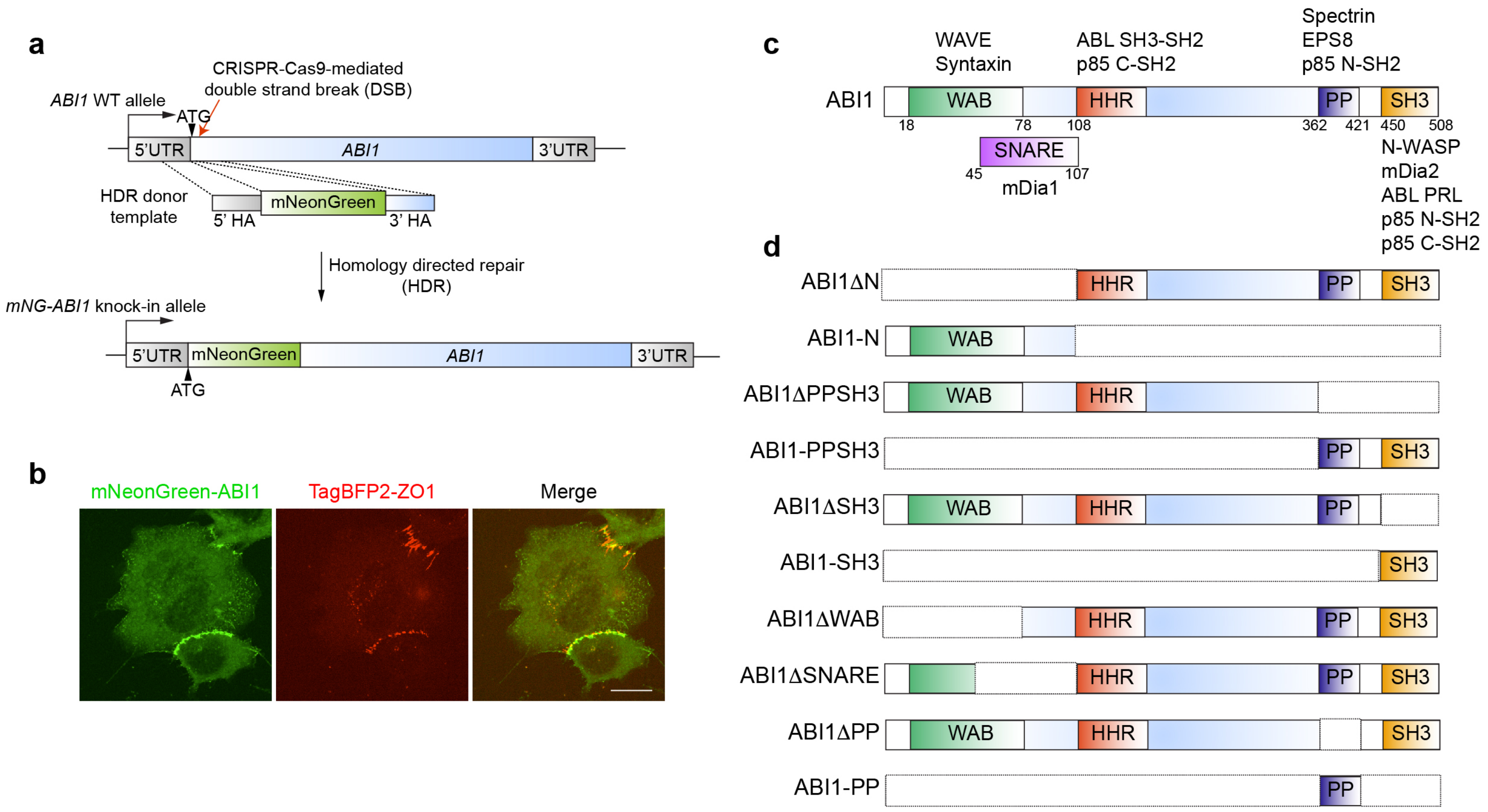
a
CRISPR-Cas9-mediated
double strand break (DSB)
ABI1 WT allele
ATG
5'UTR
ABI1
3'UTR
HDR donor
template
mNeonGreen
5' HA
3' HA
Homology directed repair
(HDR)
mNG-ABI1 knock-in allele
5'UTR
mNeonGreen
ABI1
3'UTR
ATG
b
mNeonGreen-ABI1
TagBFP2-ZO1
Merge
c
WAVE
Syntaxin
ABL SH3-SH2
p85 C-SH2
Spectrin
EPS8
p85 N-SH2
ABI1
WAB
HHR
PP
SH3
18
78
108
362
421
450
508
SNARE
45
107
mDia1
N-WASP
mDia2
ABL PRL
p85 N-SH2
p85 C-SH2
d
ABI1ΔN
ABI1-N
ABI1ΔPPSH3
ABI1-PPSH3
ABI1ΔSH3
ABI1-SH3
ABI1ΔWAB
ABI1ΔSNARE
ABI1ΔPP
ABI1-PP

Supplementary Fig 5

a, Schematic illustration for the generation of the mNeonGreen-ABI1 knock-in HeLa reporter cell line. The homology-directed repair donor (HDR) template, which carries the mNeonGreen (mNG) coding sequence along with an *ABI1* 5' homologous arm (5' HA) and 3' homologous arm (3' HA) was assembled into the pUC19 vector using the Gibson assembly method. Linearized HDR template, along with pSpCas9-sgABI1-N expressing the Cas9 protein and guide RNA targeting *ABI1*, were co-transfected into HeLa cells. Successful knock-in cells were isolated and expanded.

b, Representative live cell images of the subcellular localization of mNG-ABI1. TagBFP2-ZO1 is shown to mark cell-cell junctions. Scale bar = 20 μm.

c, Schematic representation of the domain structure of full-length ABI1. ABI1 interacting partners and their respective interacting domains are shown. WAB: WAVE Binding; HHR: Homeo-domain Homologous Region; PP: Polyproline rich region; SH2: SRC Homology 2 domain; SH3: SRC Homology 3 domain; ABL SH3-SH2: c-Abl kinase SH3-SH2 domain; ABL PRL: c-Abl kinase Proline Rich Region; p85 regulatory subunit of PI-3 kinase and its subdomains: N-SH2, N-terminal Src Homology 2 domain; C-SH2, C-terminal Src Homology 2 domain.

d, Schematic representation of ABI1 truncation variants used in this study.

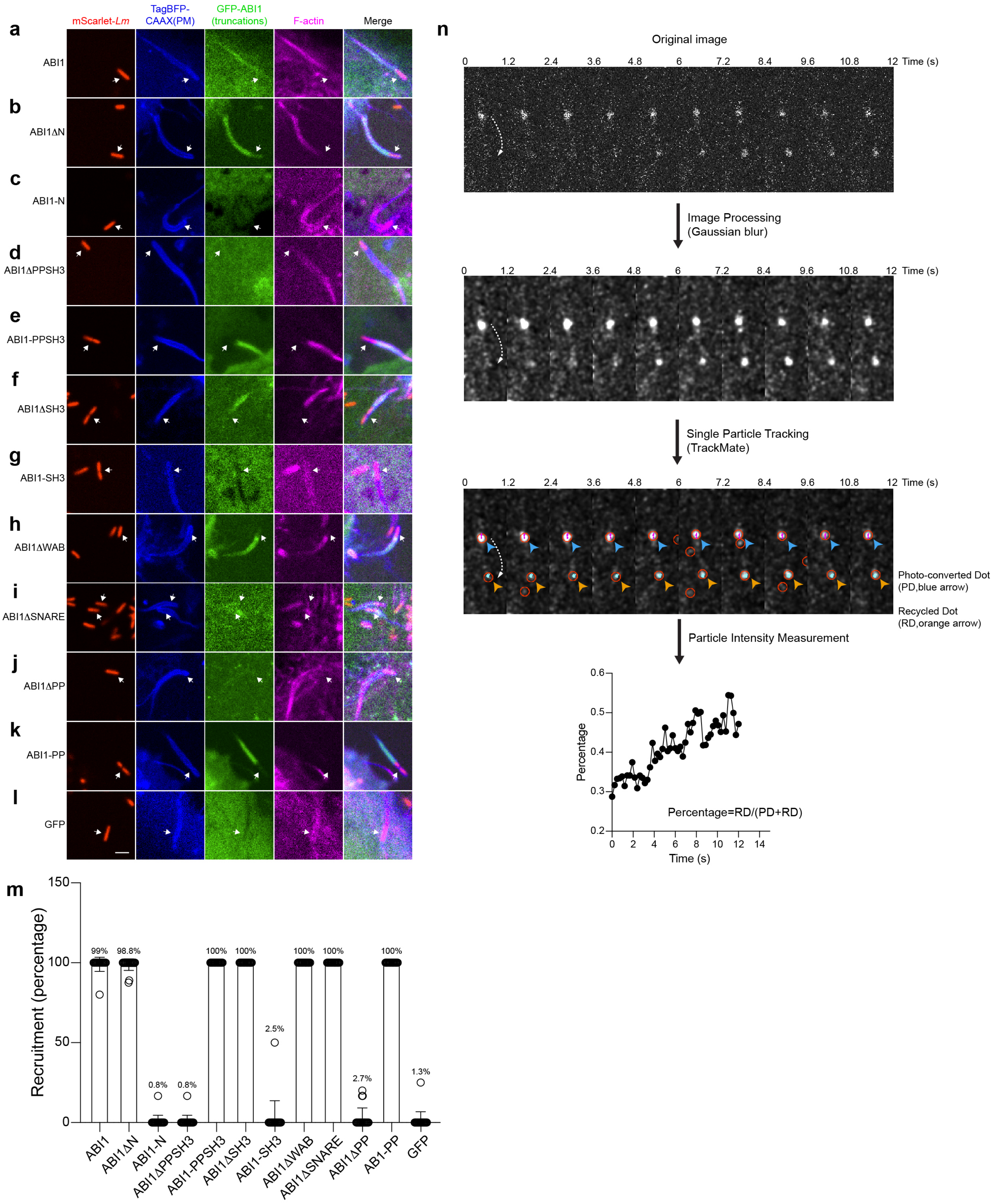

mScarlet-Lm
TagBFP-CAAX(PM)
GFP-ABI1 (truncations)
F-actin
Merge
a ABI1
b ABI1ΔN
c ABI1-N
d ABI1ΔPPSH3
e ABI1-PPSH3
f ABI1ΔSH3
g ABI1-SH3
h ABI1ΔWAB
i ABI1ΔSNARE
j ABI1ΔPP
k ABI1-PP
l GFP
m
Recruitment (percentage)
150
100
50
0
99%
98.8%
0.8%
0.8%
100%
100%
2.5%
100%
100%
2.7%
100%
1.3%
ABI1
ABI1ΔN
ABI1-N
ABI1ΔPPSH3
ABI1-PPSH3
ABI1ΔSH3
ABI1-SH3
ABI1ΔWAB
ABI1ΔSNARE
ABI1ΔPP
AB1-PP
GFP
n
Original image
0 1.2 2.4 3.6 4.8 6 7.2 8.4 9.6 10.8 12 Time (s)
Image Processing (Gaussian blur)
Single Particle Tracking (TrackMate)
Photo-converted Dot (PD,blue arrow)
Recycled Dot (RD,orange arrow)
Particle Intensity Measurement
Percentage
0.6
0.5
0.4
0.3
0.2
0 2 4 6 8 10 12 14
Time (s)
Percentage=RD/(PD+RD)

Supplementary Fig 6

a-**l**, Representative live cell images of the subcellular localization of various GFP-tagged ABI1 constructs: GFP-ABI1 (**a**), GFP-ABI1ΔN (**b**), GFP-ABI1-N (**c**), GFP-ABI1ΔPPSH3 (**d**), GFP-ABI1-PPSH3 (**e**), GFP-ABI1ΔSH3 (**f**), GFP-ABI1-SH3 (**g**), GFP-ABI1ΔWAB (**h**), GFP-ABI1ΔSNARE (**i**), GFP-ABI1ΔPP (**j**), GFP-ABI1-PP (**k**), and GFP (**l**). *ABI1* KO HeLa cells were transfected with GFP-tagged ABI1 constructs with TagBFP-CAAX (to visualize the plasma membrane (PM)), and LifeAct-iRFP670 (to visualize F-actin). The cells were subsequently infected with *Lm* 10403S expressing mScarlet (mScarlet-*Lm*). Arrowheads mark *Lm* associated with a protrusion. Scale bar = 2 μm.
m, Recruitment of full-length GFP-ABI1 (**a**), truncated GFP-ABI1 constructs (**b**-**k**), and GFP alone (**l**) to *Lm*-containing protrusions ($Actin^+$, $TagBFP\text{-}CAAX^+$). GFP-ABI1 recruitment was calculated as the number of GFP-positive *Lm*-containing protrusions divided by the total number of quantified *Lm*-containing protrusions within a single infected cell (N = 20 cells). Data are presented as mean ±SD with all data points shown and the percent GFP-ABI1 recruitment indicated.
n, Example photoconversion experiment quantification. Original image was selected and processed by applying Gaussian blur filter to enhance the contrast and trackability of the photo-converted signals. Photo-converted dot (PD, blue arrow) as well as recycled dot (RD, orange arrow) were tracked using the Fiji TrackMate plugin. The intensity of the tracked particle was measured, and the dynamic changes in the percentage of total signal intensity trafficked back to the bacterial pole (RD/(PD+RD)) over time were plotted. Dashed arrows indicate the direction of actin flow.
Source data are provided as a Source Data file.

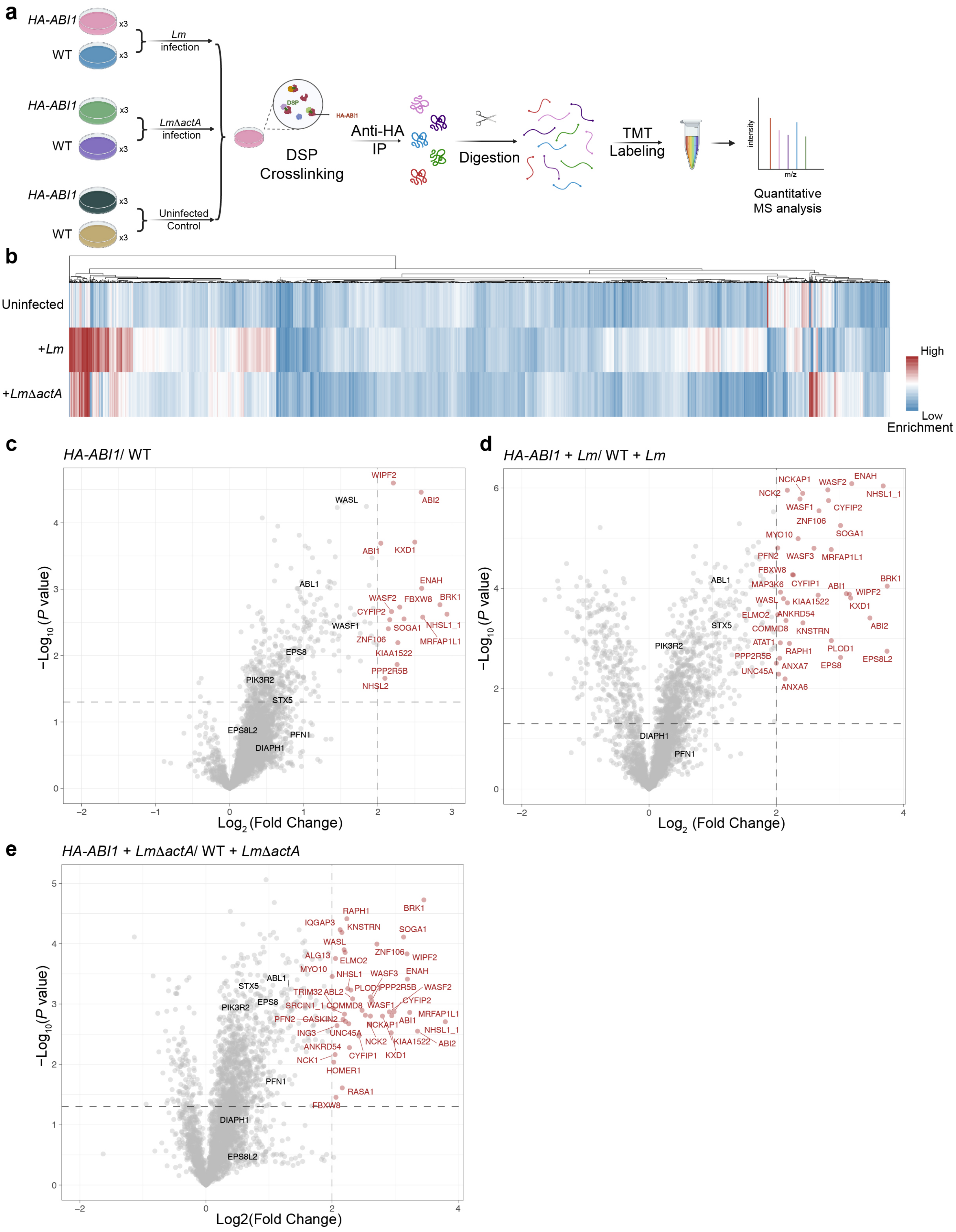
a
HA-ABI1
WT
x3
Lm
infection
LmΔactA
infection
Uninfected
Control
DSP
HA-ABI1
DSP
Crosslinking
Anti-HA
IP
Digestion
TMT
Labeling
intensity
m/z
Quantitative
MS analysis
b
Uninfected
+Lm
+LmΔactA
High
Low
Enrichment
c
HA-ABI1/ WT
−Log10(P value)
Log2 (Fold Change)
WIPF2
WASL
ABI2
ABI1
KXD1
ABL1
ENAH
WASF2
FBXW8
BRK1
CYFIP2
WASF1
SOGA1
NHSL1_1
ZNF106
MRFAP1L1
EPS8
KIAA1522
PPP2R5B
PIK3R2
NHSL2
STX5
EPS8L2
PFN1
DIAPH1
d
HA-ABI1 + Lm/ WT + Lm
−Log10(P value)
Log2 (Fold Change)
NCKAP1
WASF2
ENAH
NCK2
NHSL1_1
WASF1
CYFIP2
ZNF106
MYO10
SOGA1
PFN2
WASF3
MRFAP1L1
FBXW8
ABL1
BRK1
MAP3K6
CYFIP1
ABI1
WIPF2
WASL
KIAA1522
KXD1
ELMO2
ANKRD54
STX5
COMMD8
KNSTRN
ABI2
PIK3R2
ATAT1
RAPH1
PLOD1
PPP2R5B
EPS8L2
ANXA7
EPS8
UNC45A
ANXA6
DIAPH1
PFN1
e
HA-ABI1 + LmΔactA/ WT + LmΔactA
−Log10(P value)
Log2(Fold Change)
RAPH1
BRK1
IQGAP3
KNSTRN
SOGA1
WASL
ALG13
ZNF106
WIPF2
ELMO2
MYO10
NHSL1
WASF3
ENAH
ABL1
STX5
TRIM32
ABL2
PLOD1
PPP2R5B
WASF2
EPS8
SRCIN1_1
COMMD8
WASF1
CYFIP2
PIK3R2
PFN2
GASKIN2
ABI1
MRFAP1L1
NCKAP1
ING3
UNC45A
NHSL1_1
NCK2
KIAA1522
ABI2
ANKRD54
CYFIP1
KXD1
NCK1
HOMER1
PFN1
RASA1
FBXW8
DIAPH1
EPS8L2

Supplementary Fig 7

a, Schematic illustration of the Quantitative Immunoprecipitation Mass Spectrometry (Quant IP-MS) analysis. To explore the profile of ABI1 interacting partners during *Lm* infection, *HA-ABI1* HeLa cells were infected with *Lm* 10403S (*Lm*) and *Lm* 10403SΔ*actA* (*Lm*Δ*actA*) for 1 hour, followed by the addition of gentamicin to the media for another 6 hours. The cells were then crosslinked with DSP treatment for 30 mins to capture the ABI1 interacting partners. Following lysis, HA-ABI1 was immunoprecipitated using anti-HA beads. The resulting immunoprecipitated samples were digested and labeled with TMT for subsequent mass spectrometry analysis.
b, ABI1 interactome data obtained by Quantitative Immunoprecipitation Mass Spectrometry (Quant IP-MS) analysis. The image denotes the relative protein abundance among uninfected, *Lm* 10403S infected and *Lm* 10403SΔ*actA* infected groups. Hierarchical clustering of the MS data using $\log_2$ normalized summed S/N (Signal to noise ratio) are shown. Three biological replicates were performed for each condition.
c, Volcano plot showing all proteins detected in the uninfected sample group by Quant IP-MS with selected proteins marked. Proteins enriched in *HA-ABI1* cells compared to WT HeLa cells (no tag background control) that meet the significance thresholds (fold change > 2, $P < 0.05$; indicated by dotted lines) are highlighted in red.
d, Volcano plot showing all proteins detected in the *Lm* 10403S infected sample group by Quant IP-MS with selected proteins marked. Enriched proteins (fold change > 2, $P < 0.05$; indicated by dotted lines) are highlighted in red.
e, Volcano plot showing all proteins detected in the *Lm* 10403SΔ*actA* infected sample group by Quant IP-MS with selected proteins marked. Enriched proteins (fold change > 2, $P < 0.05$; indicated by dotted lines) are highlighted in red.
Fold change was calculated by comparing the *HA-ABI1* groups to the HeLa WT groups using the summed signal-to-noise (S/N) ratio of each detected protein. The two-tailed unpaired student's *t*-test (equal variance) was used to assess statistical significance from 3 biological repeats (**c**-**e**). The diagram in **a** was created in BioRender. Sun, H. (2026) https://BioRender.com/wwbb7ml.

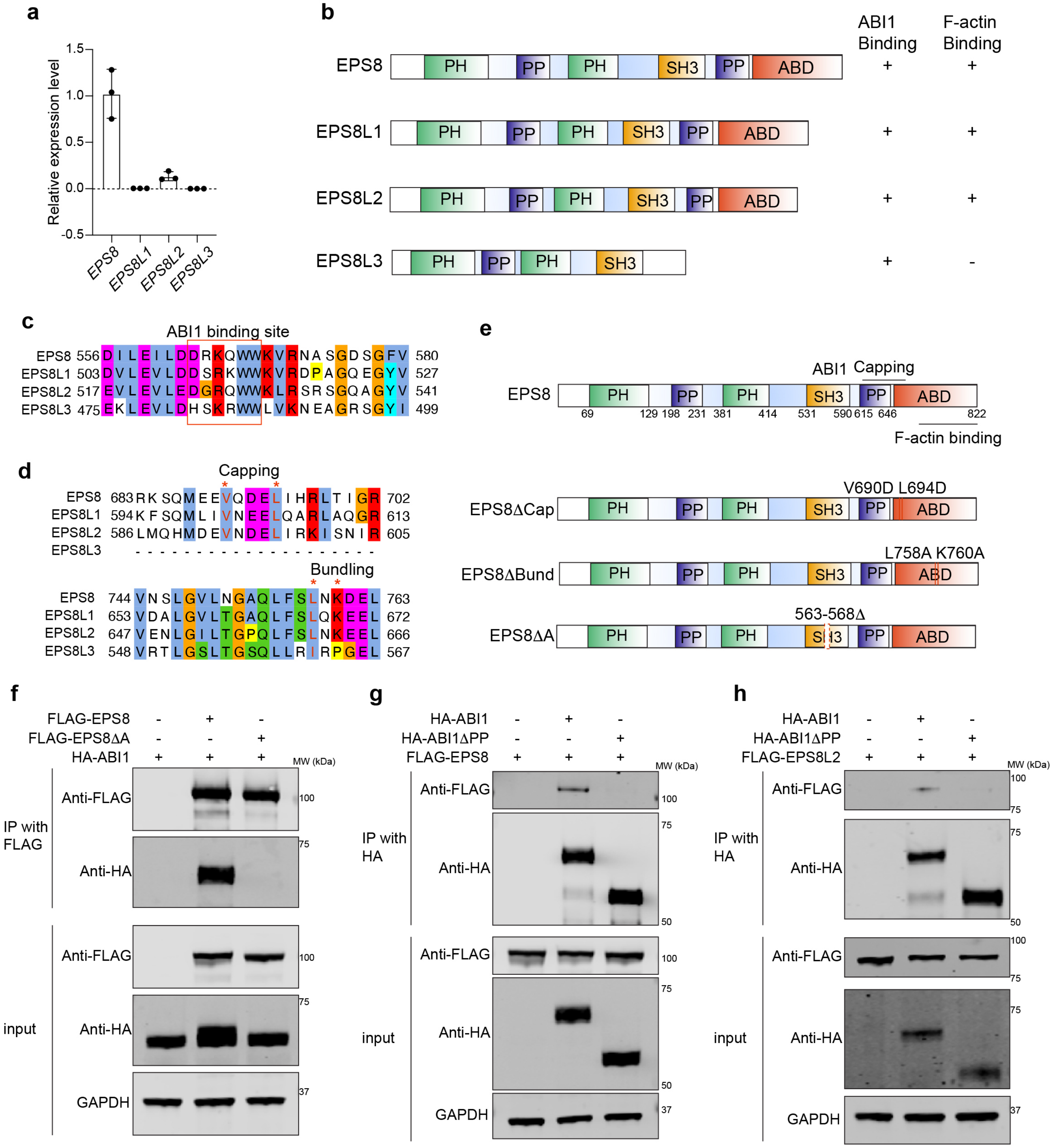

a
Relative expression level
1.5
1.0
0.5
0.0
-0.5
EPS8
EPS8L1
EPS8L2
EPS8L3
b
ABI1 Binding
F-actin Binding
EPS8
EPS8L1
EPS8L2
EPS8L3
PH
PP
SH3
ABD
+
-
c
ABI1 binding site
EPS8 556 DILEILDDRKQWWKVRNASGDSGFV 580
EPS8L1 503 DVLEVLDDSRKWWKVRDPAGQEGYV 527
EPS8L2 517 EVLEVLEDGRQWWKLRSRSGQAGYV 541
EPS8L3 475 EKLEVLDHSKRWWLVKNEAGRSGYI 499
d
Capping
EPS8 683 RKSQMEEVQDELIHRLTIGR 702
EPS8L1 594 KFSQMLIVNEELQARLAQGR 613
EPS8L2 586 LMQHMDEVNDELIRKISNIR 605
EPS8L3
Bundling
EPS8 744 VNSLGVLNGAQLFSLNKDEL 763
EPS8L1 653 VDALGVLTGAQLFSLQKEEL 672
EPS8L2 647 VENLGILTGPQLFSLNKEEL 666
EPS8L3 548 VRTLGSLTGSQLLRIRPGEL 567
e
ABI1
Capping
EPS8
69
129
198
231
381
414
531
590
615
646
822
F-actin binding
V690D L694D
EPS8ΔCap
L758A K760A
EPS8ΔBund
563-568Δ
EPS8ΔA
f
FLAG-EPS8
FLAG-EPS8ΔA
HA-ABI1
MW (kDa)
IP with FLAG
Anti-FLAG
Anti-HA
input
GAPDH
100
75
37
g
HA-ABI1
HA-ABI1ΔPP
FLAG-EPS8
IP with HA
50
h
FLAG-EPS8L2

Supplementary Fig 8

a, Relative mRNA expression levels of *EPS8*, *EPS8L1*, *EPS8L2*, and *EPS8L3* in HeLa cells as determined by qRT-PCR. N = 3 biological replicates.
b, Schematic representation comparing the domain structure of EPS8 with its isoforms EPS8L1, EPS8L2, and EPS8L3. ABI1 binding and F-actin binding abilities are indicated, respectively. PH: Pleckstrin Homology domain; PP: Polyproline rich domain; SH3: SRC Homology 3 domain; ABD: Actin Binding Domain.
c, Sequence alignment of ABI1 binding regions within EPS8 isoforms generated using the ClustalW multiple sequence alignment program with the default color scheme (Hydrophobic: light blue; Positive charge: red; Negative charge: magenta; Polar: green; Glycines: orange; Prolines: yellow; Aromatic: cyan; Unconserved: white). Conserved ABI1 binding site is indicated by a red box.
d, Sequence alignment of the sites responsible for F-actin capping and bundling activity within EPS8 isoforms generated using the ClustalW multiple sequence alignment program with the default color scheme. Key residues V690 and L694 (capping), and L758 and K760 (bundling) are highlighted in red and marked with asterisks.
e, Schematic representation of the domain structure of full-length EPS8 and EPS8 mutant variants.
f, Co-immunoprecipitation (Co-IP) assay demonstrating the interaction between ABI1 and either EPS8 or EPS8ΔA in HEK293T cells transfected with plasmids expressing the indicated proteins. GAPDH was detected as a loading control.
g, Co-IP assay demonstrating the interaction between EPS8 and either ABI1 or ABI1ΔPP in HEK293T cells transfected with plasmids expressing the indicated proteins. GAPDH was detected as a loading control.
h, Co-IP assay demonstrating the interaction between EPS8L2 and either ABI1 or ABI1ΔPP in HEK293T cells transfected with plasmids expressing the indicated proteins. GAPDH was detected as a loading control.
Source data are provided as a Source Data file.

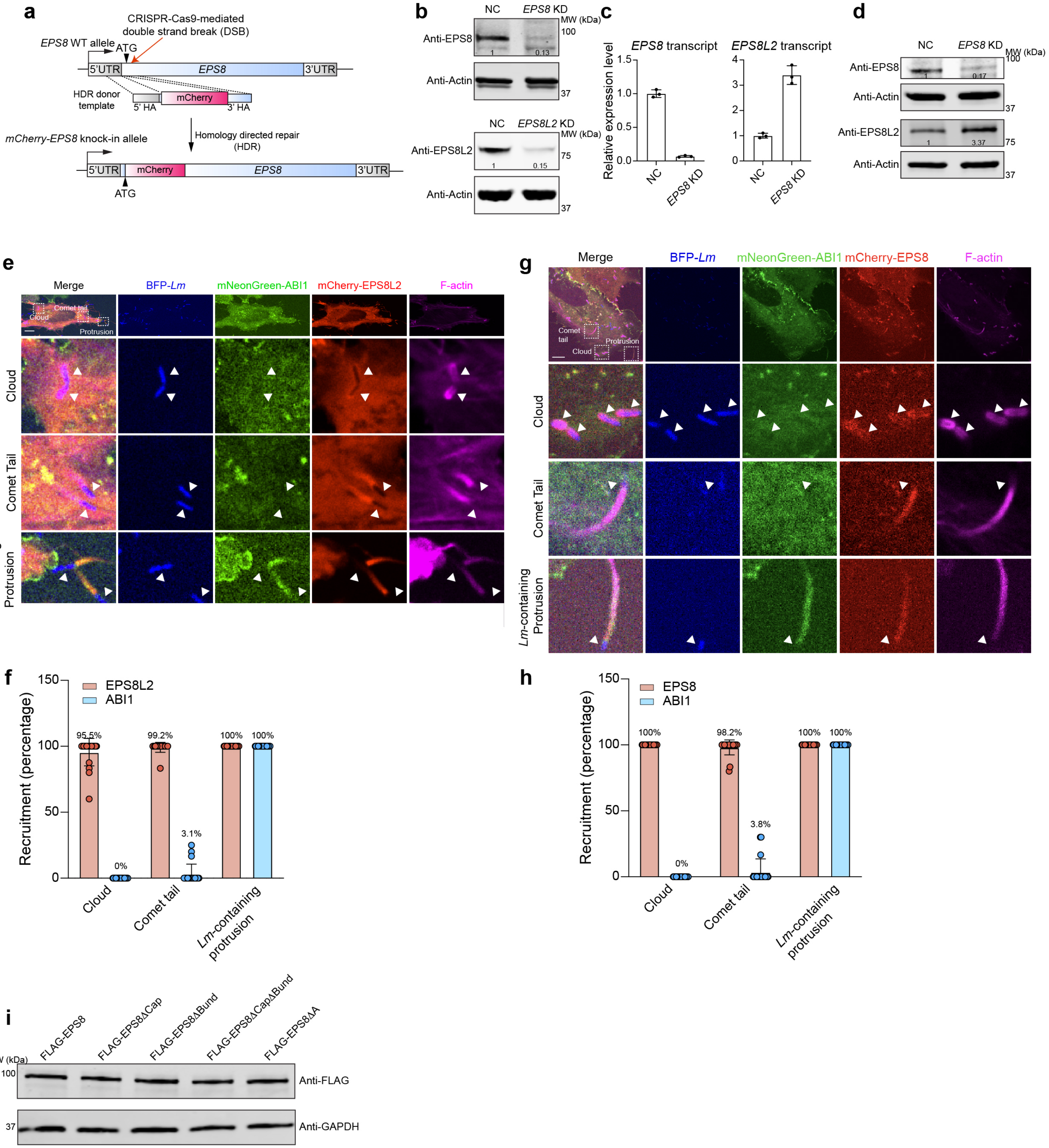
a
CRISPR-Cas9-mediated double strand break (DSB)
EPS8 WT allele
ATG
5'UTR
EPS8
3'UTR
HDR donor template
5' HA
mCherry
3' HA
Homology directed repair (HDR)
mCherry-EPS8 knock-in allele
b
NC
EPS8 KD
MW (kDa)
Anti-EPS8
Anti-Actin
EPS8L2 KD
Anti-EPS8L2
c
EPS8 transcript
EPS8L2 transcript
Relative expression level
d
e
Merge
BFP-Lm
mNeonGreen-ABI1
mCherry-EPS8L2
F-actin
Cloud
Comet Tail
Lm-containing Protrusion
f
Recruitment (percentage)
EPS8L2
ABI1
95.5%
99.2%
100%
0%
3.1%
Comet tail
Lm-containing protrusion
g
mCherry-EPS8
h
EPS8
98.2%
3.8%
i
FLAG-EPS8
FLAG-EPS8ΔCap
FLAG-EPS8ΔBund
FLAG-EPS8ΔCapΔBund
FLAG-EPS8ΔA
Anti-FLAG
Anti-GAPDH

Supplementary Fig 9

a, Schematic illustration for the generation of the mCherry-EPS8 knock-in HeLa reporter cell line. The homology-directed repair donor (HDR) template, which carries the mCherry coding sequence along with an *EPS8* 5' homologous arm (5' HA) and 3' homologous arm (3' HA) was assembled into the pUC19 vector using the Gibson assembly method. Linearized HDR template, along with pSpCas9-sgEPS8-N expressing the Cas9 protein and guide RNA targeting *EPS8*, were co-transfected into HeLa cells. Successful knock-in cells were isolated and expanded.
b, Western blot analysis detecting EPS8 or EPS8L2 protein expression using an antibody against EPS8 or EPS8L2. Detection of β-Actin was used as a loading control. HeLa cells were transfected with control endoribonuclease-prepared small interfering RNA (esiRNA, NC), esiRNA targeting *EPS8* (*EPS8* KD) or esiRNA targeting *EPS8L2* (*EPS8L2* KD). EPS8 and EPS8L2 band intensities are shown below the respective bands. EPS8 and EPS8L2 band intensities were normalized to actin with the NC sample set to 1.
c, Relative mRNA expression levels of *EPS8* and *EPS8L2* in HeLa cells transfected with control esiRNA (NC) or esiRNA targeting *EPS8* (*EPS8* KD). N = 3 biological replicates.
d, EPS8 and EPS8L2 protein expression levels after *EPS8* KD determined by Western blot analysis using antibodies against EPS8 and EPS8L2. Detection of β-Actin was used as a loading control. EPS8 and EPS8L2 band intensities are shown below the respective bands. EPS8 and EPS8L2 band intensities were normalized to actin with the NC sample set to 1.
e, Representative live-cell images of mNeonGreen-ABI1 and mCherry-EPS8L2 subcellular localization. HeLa cells expressing mNeonGreen-ABI1 were transfected with a construct encoding mCherry-EPS8L2, along with LifeAct-iRFP670 (to visualize F-actin), and subsequently infected with *Lm* 10403S expressing TagBFP (BFP-*Lm*). Scale bar = 10 μm. The enlarged insets show *Lm* associated with actin clouds, comet tails, and protrusions. Arrowheads mark *Lm* associated with actin clouds, comet tails and protrusions.
f, Recruitment of mNeonGreen-ABI1 and mCherry-EPS8L2 to actin clouds (Cloud), comet tails, and *Lm*-containing protrusions from (**e**). EPS8L2 or ABI1 recruitment was calculated as the number of mCherry-EPS8L2-positive or mNeonGreen-ABI1-positive Clouds, comet tails or *Lm*-containing protrusions divided by the total number of quantified Clouds, comet tails or *Lm*-containing protrusions within a single infected cell (N = 20 cells). Data are presented as mean ±SD with all data points shown and the percent EPS8L2 or ABI1 recruitment indicated.
g, Representative live-cell images of mNeonGreen-ABI1 and mCherry-EPS8 subcellular localization. HeLa cells expressing mNeonGreen-ABI1 were transfected with a construct encoding mCherry-EPS8, along with LifeAct-iRFP670 (to visualize F-actin), and subsequently infected with *Lm* 10403S expressing TagBFP (BFP-*Lm*). Scale bar = 10 μm. The enlarged insets show *Lm* associated with actin clouds, comet tails, and protrusions. Arrowheads mark *Lm* associated with actin clouds, comet tails and protrusions.
h, Recruitment of mNeonGreen-ABI1 and mCherry-EPS8 to actin clouds (Cloud), comet tails, and *Lm*-containing protrusions from (**g**). EPS8 or ABI1 recruitment was calculated as the number of mCherry-EPS8-positive or mNeonGreen-ABI1-positive Clouds, comet tails or *Lm*-containing protrusions divided by the total number of quantified Clouds, comet tails or *Lm*-containing protrusions within a single infected cell (N = 20 cells). Data are presented as mean ±SD with all data points shown and the percent EPS8 or ABI1 recruitment indicated.
i, Western blot analysis of EPS8 variant expression in HeLa cells. Expression levels of EPS8 mutants were detected using an anti-FLAG antibody with GAPDH as a loading control.
Source data are provided as a Source Data file.

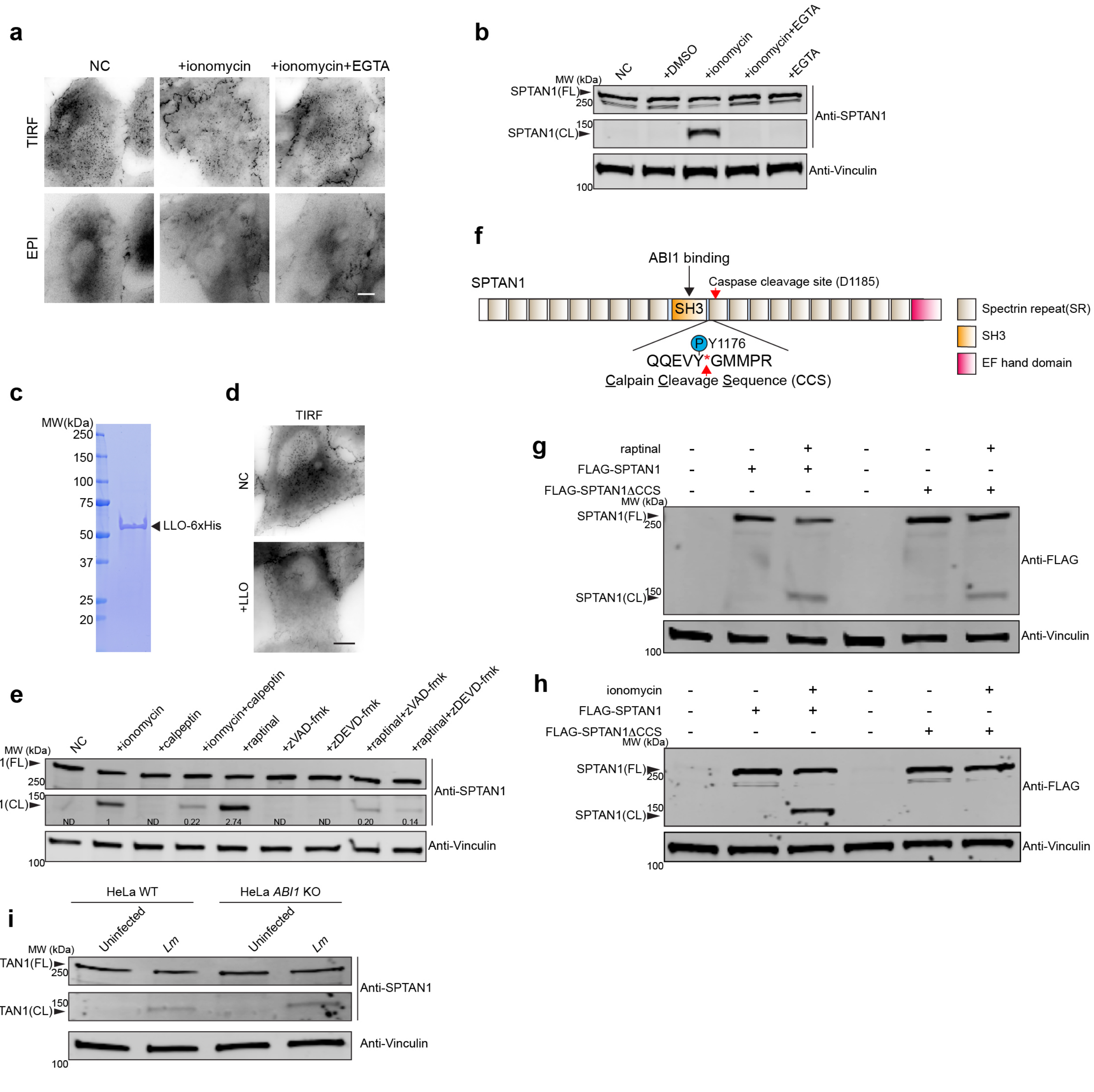

a
NC
+ionomycin
+ionomycin+EGTA
TIRF
EPI
b
NC
+DMSO
+ionomycin
+ionomycin+EGTA
+EGTA
MW (kDa)
SPTAN1(FL)
250
150
SPTAN1(CL)
Anti-SPTAN1
Anti-Vinculin
100
f
ABI1 binding
SPTAN1
Caspase cleavage site (D1185)
SH3
P Y1176
QQEVY*GMMPR
Calpain Cleavage Sequence (CCS)
Spectrin repeat(SR)
SH3
EF hand domain
c
MW(kDa)
250
150
100
75
50
37
25
20
LLO-6xHis
d
TIRF
NC
+LLO
g
raptinal
FLAG-SPTAN1
FLAG-SPTAN1ΔCCS
MW (kDa)
SPTAN1(FL)
250
150
SPTAN1(CL)
Anti-FLAG
Anti-Vinculin
100
e
NC
+ionomycin
+calpeptin
+ionmycin+calpeptin
+raptinal
+zVAD-fmk
+zDEVD-fmk
+raptinal+zVAD-fmk
+raptinal+zDEVD-fmk
MW (kDa)
SPTAN1(FL)
250
150
SPTAN1(CL)
ND 1 ND 0.22 2.74 ND ND 0.20 0.14
Anti-SPTAN1
Anti-Vinculin
100
h
ionomycin
FLAG-SPTAN1
FLAG-SPTAN1ΔCCS
MW (kDa)
SPTAN1(FL)
250
150
SPTAN1(CL)
Anti-FLAG
Anti-Vinculin
100
i
HeLa WT
HeLa ABI1 KO
Uninfected
Lm
Uninfected
Lm
MW (kDa)
SPTAN1(FL)
250
150
SPTAN1(CL)
Anti-SPTAN1
Anti-Vinculin
100

Supplementary Fig 10

a, HeLa cells expressing mNeonGreen-ABI1 were treated with 5 μM ionomycin alone or ionomycin in combination with 1 mM EGTA. Images were captured one-hour post-treatment using either TIRF (upper panels) or epifluorescence (EPI, lower panels) microscopy. Scale bar = 10 μm.
b, Western blot analysis of SPTAN1 cleavage in HeLa cells treated for 30 minutes with 5 μM ionomycin, ionomycin in combination with 1 mM EGTA, EGTA alone, DMSO (solvent control) or untreated (NC). Full-length (FL) or cleaved (CL) SPTAN1 were detected using an anti-SPTAN1 antibody with Vinculin as a loading control.
c, Purified recombinant LLO with a C-terminal 6xHis tag was analyzed by SDS-PAGE and visualized by Coomassie blue staining.
d, HeLa cells expressing mNeonGreen-ABI1 were treated with 0.5 nM purified recombinant LLO. Images were captured 30 minutes post-treatment using TIRF microscopy. Scale bar=10 μm.
e, Western blot analysis of SPTAN1 cleavage in HeLa cells treated for 1 hour with ionomycin (5 μM), raptinal (10 μM, caspase-3 activator), or the indicated inhibitors. Calpeptin (20 μM) was used as a calpain inhibitor, while zVAD-fmk (20 μM, pan-caspase inhibitor) and zDEVD-fmk (30 μM, caspase-3 inhibitor) were used to block caspase activity. Full-length (FL) and cleaved (CL) SPTAN1 were detected using an anti-SPTAN1 antibody with Vinculin as a loading control. SPTAN1(CL) band intensities are shown below the respective bands. SPTAN1(CL) band intensities were normalized to Vinculin, with the ionomycin treatment sample set to 1. ND = not detected.
f, Schematic representation of the domain structure of SPTAN1. The Calpain Cleavage Sequence (CCS) are shown, along with the ABI1-binding and caspase cleavage site (D1185). SH3: SRC Homology 3 domain.
g, Western blot analysis of SPTAN1 cleavage in WT HeLa cells or HeLa cells expressing FLAG-SPTAN1 or cleavage-resistant FLAG-SPTAN1ΔCCS in response to raptinal (10 μM, caspase-3 activator). Full-length (FL) and cleaved (CL) SPTAN1 were detected using an anti-FLAG antibody, with Vinculin serving as a loading control.
h, Western blot analysis of SPTAN1 cleavage in WT HeLa cells or HeLa cells expressing FLAG-SPTAN1 or cleavage-resistant FLAG-SPTAN1ΔCCS in response to ionomycin (5 μM). Full-length (FL) and cleaved (CL) SPTAN1 were detected using an anti-FLAG antibody, with Vinculin serving as a loading control.
i, Western blot analysis of SPTAN1 cleavage in WT or *ABI1* KO HeLa cells at 6 hpi with *Lm* 10403S (*Lm*). Full-length (FL) and cleaved (CL) SPTAN1 were detected using an anti-SPTAN1 antibody, with Vinculin serving as a loading control.
Source data are provided as a Source Data file.
