# Supplementary material for: A pore-forming toxin initiates ABI1 complex switching to promote bacterial cell-to-cell spread
Source: Nat Commun. 2026 Apr 13;17:5129. doi: 10.1038/s41467-026-71510-z (PMC13247217; doi:10.1038/s41467-026-71510-z)
Supplement: Supplementary file 2 — Description of Additional Supplementary Files [file 41467_2026_71510_MOESM2_ESM.pdf]

Description of Additional Supplementary Files:

Supplementary Data 1: Bacterial strains and plasmids used in this study.

Supplementary Data 2: Primers, oligonucleotides, and antibodies used in this study.

Supplementary Data 3: Raw data of quantitative mass spectrometry.

Supplementary Movie 1: Representative time-lapse video depicting mNeonGreenABI1 subcellular localization. HeLa cells expressing mNeonGreen-ABI1 (green channel) and EZRIN-TagRFP (red channel) were infected with BFP-*Lm* (blue channel). Actin was visualized using SiR-actin (magenta channel). Images were captured at 5-7 hours post-infection. Scale bar = 20 μm.

Supplementary Movie 2: Representative time-lapse video of mEOS3.2-Actin (green) and photo-converted mEOS3.2-Actin (red) dynamics within 10 seconds after photo-conversion in *Lm*-containing protrusions in WT and *ABI1* KO HeLa cells. Scale bar = 2 μm.

Supplementary Movie 3: Representative time-lapse video depicting mNeonGreenABI1 and mCherry-EPS8 subcellular localization. HeLa cells expressing mNeonGreen-ABI1 (green channel) and mCherry-EPS8 (red channel) were infected with BFP-*Lm* (blue channel). Actin was visualized using SiR-actin (magenta channel). Images were captured at 5-7 hours post-infection. Scale bar = 20 μm.

Supplementary Movie 4: Representative time-lapse video depicting mNeonGreenABI1 dynamics using TIRF microscopy. HeLa cells expressing mNeonGreenABI1 were treated with 5 μM ionomycin alone or ionomycin in combination with 1 mM EGTA. Images were captured one-hour post-treatment. Scale bar = 2 μm.

Supplementary Movie 5: Representative time-lapse video depicting mNeonGreenABI1 dynamics using TIRF microscopy. HeLa cells expressing mNeonGreenABI1 were infected with Lm 10403S (*Lm*), *Lm Δhly*, or complemented *Lm Δhly+hly*. Images were captured at 5-7 hours post-infection. Scale bar = 2 μm.

Supplementary Movie 6: Representative time-lapse video depicting mNeonGreenABI1 dynamics using TIRF microscopy. HeLa cells expressing mNeonGreenABI1 were treated for 30 minutes with purified recombinant LLO. Scale bar = 2 μm.
