# Supplementary material for: A pore-forming toxin initiates ABI1 complex switching to promote bacterial cell-to-cell spread
Source: Nat Commun. 2026 Apr 13;17:5129. doi: 10.1038/s41467-026-71510-z (PMC13247217; doi:10.1038/s41467-026-71510-z)
Supplement: Supplementary file 12 — Reporting Summary [file 41467_2026_71510_MOESM12_ESM.pdf]

nature portfolio

Corresponding author(s): Darren E. Higgins

Last updated by author(s): Feb 27, 2026

Reporting Summary

Nature Portfolio wishes to improve the reproducibility of the work that we publish. This form provides structure for consistency and transparency in reporting. For further information on Nature Portfolio policies, see our Editorial Policies and the Editorial Policy Checklist.

Statistics

For all statistical analyses, confirm that the following items are present in the figure legend, table legend, main text, or Methods section.

n/a	Confirmed	
☐	☒	The exact sample size (*n*) for each experimental group/condition, given as a discrete number and unit of measurement
☐	☒	A statement on whether measurements were taken from distinct samples or whether the same sample was measured repeatedly
☐	☒	The statistical test(s) used AND whether they are one- or two-sided<br>*Only common tests should be described solely by name; describe more complex techniques in the Methods section.*
☒	☐	A description of all covariates tested
☒	☐	A description of any assumptions or corrections, such as tests of normality and adjustment for multiple comparisons
☐	☒	A full description of the statistical parameters including central tendency (e.g. means) or other basic estimates (e.g. regression coefficient) AND variation (e.g. standard deviation) or associated estimates of uncertainty (e.g. confidence intervals)
☐	☒	For null hypothesis testing, the test statistic (e.g. *F*, *t*, *r*) with confidence intervals, effect sizes, degrees of freedom and *P* value noted<br>*Give P values as exact values whenever suitable.*
☒	☐	For Bayesian analysis, information on the choice of priors and Markov chain Monte Carlo settings
☒	☐	For hierarchical and complex designs, identification of the appropriate level for tests and full reporting of outcomes
☒	☐	Estimates of effect sizes (e.g. Cohen's *d*, Pearson's *r*), indicating how they were calculated

Our web collection on statistics for biologists contains articles on many of the points above.

Software and code

Policy information about availability of computer code

Data collection	Microscopy data were collected using vendor-provided acquisition software, including Nikon NIS-Elements for spinning-disk confocal imaging, Zeiss Zen Blue for point-scanning confocal and Airyscan imaging, and Olympus VS200 software for whole-slide scanning. Immunoblot images were acquired using LI-COR Image Studio on the Odyssey® DLx Imaging System. No custom code was used for data collection.
Data analysis	Image processing and quantitative analyses were performed using Fiji (ImageJ,version: 2.3.0/1.54m, NIH), including plaque area quantification, fluorescence intensity measurements, and single-particle tracking using the TrackMate plugin. Statistical analyses and data visualization were performed using GraphPad Prism (v9.1.1), Microsoft Excel(version 16.106), and R (version 4.4.2). Gene Ontology (GO) enrichment analysis was performed using the enrichGO function in the R package ClusterProfiler (v4.14.6). Figure assembly was performed using Adobe Illustrator 2024 and Adobe Photoshop 2024. No custom-written code was used.

For manuscripts utilizing custom algorithms or software that are central to the research but not yet described in published literature, software must be made available to editors and reviewers. We strongly encourage code deposition in a community repository (e.g. GitHub). See the Nature Portfolio guidelines for submitting code & software for further information.

Data

Policy information about availability of data

All manuscripts must include a data availability statement. This statement should provide the following information, where applicable:

- Accession codes, unique identifiers, or web links for publicly available datasets
- A description of any restrictions on data availability
- For clinical datasets or third party data, please ensure that the statement adheres to our policy

All data supporting the findings of this study are available within the Article and its Supplementary Information. Mass spectrometry data used for analysis can be found in Supplementary Data 3. The protein mass spectrometry raw data generated in this study have been deposited in the ProteomeXchange Consortium via the PRIDE partner repository under accession code PXD074581 (https://www.ebi.ac.uk/pride/archive/projects/PXD074581). Bacterial strains, plasmids, cell lines, and mouse lines will be made available from the authors upon request. Further information and requests for resources and reagents should be directed to the lead contact, Dr. Darren Higgins (darren_higgins@hms.harvard.edu). Source data are provided with this manuscript.

No software or code was generated for this study.

Research involving human participants, their data, or biological material

Policy information about studies with human participants or human data. See also policy information about sex, gender (identity/presentation), and sexual orientation and race, ethnicity and racism.

Reporting on sex and gender	Human research participants are not applicable to this study.
Reporting on race, ethnicity, or other socially relevant groupings	Human research participants are not applicable to this study.
Population characteristics	Human research participants are not applicable to this study.
Recruitment	Human research participants are not applicable to this study.
Ethics oversight	Human research participants are not applicable to this study.

Note that full information on the approval of the study protocol must also be provided in the manuscript.

Field-specific reporting

Please select the one below that is the best fit for your research. If you are not sure, read the appropriate sections before making your selection.

[x] Life sciences [ ] Behavioural & social sciences [ ] Ecological, evolutionary & environmental sciences

For a reference copy of the document with all sections, see nature.com/documents/nr-reporting-summary-flat.pdf

Life sciences study design

All studies must disclose on these points even when the disclosure is negative.

Sample size	No sample size calculation was performed and samples sizes were chosen based on common practice in the field and the variability within an experiment.
Data exclusions	All data were obtained using optimized protocols, and no data were excluded from analyses.
Replication	Findings were reproduced at least 3 times with biological replicates. All attempts to replicate experiments were successful. Mass spectrometry (MS) was performed once, with hits subsequently verified by co-immunoprecipitation (Co-IP).
Randomization	The experiments were not randomized. The experiments investigated specific mice lines, cell types and treatments and required precise control over the experimental conditions, aspects that are not conditions amenable to randomization.
Blinding	Investigators were not blinded to group allocation during experiments or outcome assessment. However, control and experimental samples were processed equally and in parallel to ensure consistency.

Reporting for specific materials, systems and methods

We require information from authors about some types of materials, experimental systems and methods used in many studies. Here, indicate whether each material, system or method listed is relevant to your study. If you are not sure if a list item applies to your research, read the appropriate section before selecting a response.

Materials & experimental systems

n/a	Involved in the study	
[ ]	[x]	Antibodies
[ ]	[x]	Eukaryotic cell lines
[x]	[ ]	Palaeontology and archaeology
[ ]	[x]	Animals and other organisms
[x]	[ ]	Clinical data
[x]	[ ]	Dual use research of concern
[x]	[ ]	Plants

Methods

n/a	Involved in the study	
[x]	[ ]	ChIP-seq
[x]	[ ]	Flow cytometry
[x]	[ ]	MRI-based neuroimaging

Antibodies

Antibodies used

Rabbit anti-ABI1 Proteintech Cat.# 27387-1-AP
Rabbit anti-EPS8 Proteintech Cat.# 12455-1-AP
Rabbit anti-EPS8L2 Proteintech Cat.# 20461-1-AP
Mouse anti-EZR Invitrogen Cat.# 35-7300
Mouse anti-GAPDH Invitrogen Cat.# AM4300
Mouse anti-Actin Millipore Sigma Cat.# A5316
Rabbit anti-LLO Abcam Cat.# ab200538
Rabbit anti-Vinculin Proteintech Cat.# 26520-1-AP
Mouse anti-SPTAN1 Enzo Life Sciences Cat.# BML-FG6090
Listeria O Antisera Fisher Scientific Cat.# DF2302-50-0
IRDye® 680RD Goat anti-Mouse IgG Secondary Antibody LI-COR Bio Cat.# 926-68070
IRDye® 680RD Goat anti-Rabbit IgG Secondary Antibody LI-COR Bio Cat.# 926-68071
IRDye® 800CW Goat anti-Mouse IgG Secondary Antibody LI-COR Bio Cat.# 926-32210
IRDye® 800CW Goat anti-Rabbit IgG Secondary Antibody LI-COR Bio Cat.# 926-32211
Goat anti-Rabbit IgG (H+L) Highly Cross-Adsorbed Secondary Antibody, Alexa Fluor™ Plus 488 Invitrogen Cat.# A32731
FluoTag®-X4 anti-Rabbit IgG-Atto488 NanoTag Biotechnologies Cat.# N2404-At488-S
FluoTag®-X2 anti-Mouse IgG1-Alexa647 NanoTag Biotechnologies Cat.# N2002-AF647-S

Validation

The rabbit anti-ABI1 antibody used in this study has been previously validated and independently used in multiple peer-reviewed publications across different biological contexts. This antibody has been applied to detect endogenous ABI1 protein in ovarian cancer cells, neuronal cells, and colorectal cancer models, where ABI1 expression or abundance was experimentally modulated and corresponding changes in signal were reported (Yamamoto et al., J Ovarian Res; Jin et al., J Psychiatr Res; Li et al., Transl Oncol). These studies support the specificity of the antibody for ABI1 under immunoblotting conditions. In addition, the manufacturer provides validation data including application-specific performance and immunoblotting specificity (Proteintech, rabbit anti-ABI1, Cat. No. 27387-1-AP; https://www.ptglab.com/products/ABI1-Antibody-27387-1-AP.htm). The antibody was used according to the manufacturer's recommendations.

The EPS8 polyclonal antibody used in this study has been previously validated and independently used in multiple peer-reviewed publications across diverse biological systems. This antibody has been applied to detect endogenous EPS8 protein in cancer cell models, including pancreatic cancer and gastric cancer, where experimental perturbation of EPS8 expression or function was associated with corresponding changes in the detected signal (Tan et al., Experimental Cell Research; Zhang et al., Acta Biochimica et Biophysica Sinica). In addition, the antibody has been used to examine EPS8-related cytoskeletal functions in sensory hair cells (Acta Otolaryngologica, Li et al.). These prior studies support the specificity of the antibody for EPS8 under immunoblotting and related applications. Further validation data, including application-specific performance, are provided by the manufacturer (Proteintech, EPS8 polyclonal antibody, Cat. No. 12455-1-AP; https://www.ptglab.com/products/EPS8-Antibody-12455-1-AP.htm). The antibody was used according to the manufacturer's recommendations.

The EPS8L2 polyclonal antibody used in this study has been previously validated and independently used in multiple peer-reviewed publications. This antibody has been applied to detect endogenous EPS8L2 in mouse brain tissues and brain endothelial cells in studies examining neuronal function, learning and memory, and neurovascular biology (Ouyang et al., Laboratory Investigation; Yu et al., Cell Reports). In these studies, EPS8L2 expression was assessed in physiologically relevant contexts, supporting the utility and specificity of the antibody for detection of EPS8L2 protein. Additional validation data, including application-specific performance, are provided by the manufacturer (Proteintech, EPS8L2 polyclonal antibody, Cat. No. 20461-1-AP; https://www.ptglab.com/products/EPS8L2-Antibody-20461-1-AP.htm). The antibody was used according to the manufacturer's recommendations.

The Ezrin monoclonal antibody (clone 3C12) used in this study has been previously validated and independently used in multiple peer-reviewed publications. This antibody has been applied to detect endogenous Ezrin in diverse cellular contexts, including studies of lysosomal biogenesis and retinal cell clearance (The EMBO Journal), podocyte signaling and apoptosis under high-glucose conditions (Biology Open), and epithelial junction integrity and cell-to-cell spread of Listeria monocytogenes (Nature Cell Biology). In these studies, Ezrin localization and/or abundance was assessed in physiologically relevant settings, supporting the specificity and utility of the antibody for detection of Ezrin protein. Additional validation data, including application-specific performance, are provided by the manufacturer (Thermo Fisher Scientific, Ezrin monoclonal antibody, clone 3C12, Cat. No. 35-7300; https://www.thermofisher.com/antibody/product/Ezrin-Antibody-clone-3C12-Monoclonal/35-7300). The antibody was used according to the manufacturer's recommendations.

The GAPDH monoclonal antibody (clone 6C5) used in this study has been previously validated and independently used in multiple peer-reviewed publications. This antibody has been applied to detect endogenous GAPDH in diverse cellular contexts, including studies of cytosolic DNA extraction and quantification (STAR Protocols), centrosome and cilia biology (The Journal of Cell Biology), and meiotic chromosome dynamics and infertility (eLife). In these studies, GAPDH was used as a cytosolic and loading control protein, and the antibody reliably detected GAPDH at the expected molecular weight across multiple mammalian cell types, supporting its specificity and suitability for immunoblotting applications. Additional validation data, including application-specific performance, are provided by the manufacturer (Thermo Fisher Scientific, GAPDH monoclonal antibody, clone 6C5, Cat. No. AM4300; https://www.thermofisher.com/antibody/product/AM4300.html). The antibody was used according to the manufacturer's recommendations.

The anti-β-Actin (ACTB) antibody used in this study has been previously validated and independently used in multiple peer-reviewed publications across diverse experimental systems. This antibody has been applied to detect endogenous β-Actin in studies of liver regeneration and cell cycle progression (Journal of Biological Chemistry; Mitchell et al.), androgen-independent prostate cancer growth (Journal of Clinical Investigation; Bernard et al.), and translational read-through mechanisms in genetic disease models (Nucleic Acid Therapeutics; Huang et al.). In these studies, β-Actin was consistently detected as a cytoskeletal and loading control protein at the expected molecular weight, supporting the specificity and suitability of the antibody for immunoblotting applications. Additional validation data, including application-specific performance, are provided by the manufacturer (Sigma-Aldrich, anti-β-Actin antibody, Cat. No. A5316; https://www.sigmaaldrich.com/US/en/product/sigma/a5316). The antibody was used according to the manufacturer's recommendations.

The anti-Listeriolysin O (LLO) antibody used in this study has been previously validated and independently used in multiple peer-reviewed publications investigating Listeria monocytogenes virulence and LLO-dependent mechanisms. This antibody has been employed in studies examining antibody–toxin conjugates for cancer immunotherapy (Nature Cancer, 2025), immunopeptidomics-guided mRNA vaccine design against Listeria monocytogenes (Nature Communications, 2022), and pharmacological inhibition of LLO-dependent pathogenicity (British Journal of Pharmacology, 2022). Across these studies, detection of LLO was performed in biologically relevant contexts where LLO expression or function was central to the experimental design, supporting the utility and specificity of the antibody for LLO detection. Additional validation data and product information are provided by the manufacturer (Abcam, anti-Listeriolysin O antibody, Cat. No. ab200538; https://www.abcam.com/en-us/products/primary-antibodies/listeriolysin-llo-antibody-ab200538). The antibody was used according to the manufacturer's recommendations.

The Vinculin polyclonal antibody used in this study has been previously validated and independently used in multiple peer-reviewed publications across diverse biological contexts. This antibody has been applied to detect endogenous Vinculin in human cells by immunoblotting and immunofluorescence in studies of cellular metabolism (Cell Metabolism; Wu et al.), epigenetic regulation and drug response in acute myeloid leukemia (Zhou et al.), endothelial cell stress responses (ACS Nano; Zhang et al.), and RNA processing mechanisms (Molecular Cell; Sun et al.). In these studies, Vinculin was detected in physiologically relevant settings consistent with its established role as a cytoskeletal and focal adhesion protein, supporting the specificity and utility of the antibody for vinculin detection. Additional validation data, including application-specific performance, are provided by the manufacturer (Proteintech, Vinculin polyclonal antibody, Cat. No. 26520-1-AP; https://www.ptglab.com/products/Vinculin-Antibody-26520-1-AP.htm). The antibody was used according to the manufacturer's recommendations.

The SPTAN1(α-Fodrin) monoclonal antibody (clone AA6) used in this study has been previously validated and independently used in multiple peer-reviewed studies examining neuronal injury, protein degradation, and calcium-dependent cell death mechanisms. This antibody has been applied for immunoblotting to detect endogenous α-Fodrin and its proteolytic fragments in studies of millimeter-wave–induced neural injury (Golpich et al., Research Square, 2025), neuronal ferroptosis following intracerebral hemorrhage (Sun et al., Cell Death & Disease, 2025), and trimethyltin-induced neurodegeneration (Onaka et al., Neuropsychopharmacology Reports, 2025). In these studies, α-Fodrin detection was used to assess cytoskeletal integrity and protease-mediated cleavage under physiologically relevant conditions, supporting the specificity and suitability of the antibody for Western blot applications. Additional validation data and product information are provided by the manufacturer (Enzo Life Sciences, α-Fodrin monoclonal antibody, clone AA6; https://www.enzo.com/product/α-fodrin-monoclonal-antibody-aa6/). The antibody was used according to the manufacturer's recommendations.

BD Difco™ Listeria O Antisera used in this study are commercially produced polyclonal antisera designed for the serological identification and classification of Listeria species based on somatic (O) antigens. These antisera are widely used for Listeria serotyping in microbiological and clinical laboratories and have been validated by the manufacturer for specificity toward defined Listeria O antigens. The reagent was used in accordance with its intended application for serological detection of Listeria O antigens. Detailed validation and usage information are provided by the manufacturer (BD Difco™, Listeria O Antisera; https://www.fishersci.com/shop/products/bd-difco-i-listeria-i-o-antisera-3/DF2300502).

The following secondary antibodies were used in this study: IRDye® 680RD Goat anti-Mouse IgG (LI-COR Biosciences, Cat. No. 926-68070), IRDye® 680RD Goat anti-Rabbit IgG (Cat. No. 926-68071), IRDye® 800CW Goat anti-Mouse IgG (Cat. No. 926-32210), IRDye® 800CW Goat anti-Rabbit IgG (Cat. No. 926-32211), Goat anti-Rabbit IgG (H+L) Highly Cross-Adsorbed Alexa Fluor™ Plus 488 (Invitrogen, Cat. No. A32731), FluoTag®-X4 anti-Rabbit IgG–Atto488 (NanoTag Biotechnologies, Cat. No. N2404-At488-S), and FluoTag®-X2 anti-Mouse IgG1–Alexa Fluor™ 647 (NanoTag Biotechnologies, Cat. No. N2002-AF647-S).

These secondary antibodies are commercially validated by the manufacturers for fluorescence-based detection of species- and isotype-specific primary antibodies and are widely used for quantitative immunoblotting and immunofluorescence applications. Cross-adsorbed secondaries were used where indicated to minimize species cross-reactivity. All secondary antibodies were used according to the manufacturers' recommendations.

Eukaryotic cell lines

Policy information about cell lines and Sex and Gender in Research

Cell line source(s)	HeLa (female, Cat.# CCL-2), HEK293T (female, Cat.# CRL-3216), HepG2 (male, Cat.# HB-8065), JEG-3 (female, Cat.# HTB-36), THP-1 (male, Cat.# TIB-202), L929 (female, murine,Cat.# CCL-1), hCMEC/D3 (female,Millipore Sigma, Cat.# SCC066), and Caco2 (male, Cat.# HTB-37) cell lines were originally obtained from ATCC or commercial vendors as indicated, or from academic sources (Dr. Judy Lieberman and Dr. Marcia Goldberg). Cell lines generated in this study include ABI1 knockout (KO), knockdown (KD), and stable expression derivatives of HeLa, Caco2, HepG2, JEG-3, hCMEC/D3, and THP-1 cells, as well as HeLa cells expressing tagged ABI1, EPS8, EZRIN, or mEOS3.2-actin constructs. All derivative cell lines retain the sex of the parental line.<br>Bone marrow–derived macrophages (BMDM) were isolated from C57BL/6J mice using standard differentiation protocols. Both male and female mice were used for BMDM isolation.
Authentication	All cells used were authenticated and tested negative for mycoplasma by ATCC.
Mycoplasma contamination	All lines tested negative for Mycoplasma contamination using the Lookout® One-Step Mycoplasma PCR Detection Kit (Sigma Aldrich, MP0050-25TST)
Commonly misidentified lines<br>(See ICLAC register)	No commonly misidentified lines were used in this study.

Animals and other research organisms

Policy information about studies involving animals; ARRIVE guidelines recommended for reporting animal research, and Sex and Gender in Research

Laboratory animals	Esr1-cre mice (B6.Cg-Tg(CAG-cre/Esr1*)5Amc/J, The Jackson Laboratory, Cat.# 004682) and Mx1-cre mice (B6.Cg-Tg(Mx1-cre)1Cgn/J, The Jackson Laboratory, Cat.# 003556) were bred in-house at The Harvard Institutes of Medicine BL-1 Animal Facility at Harvard Medical School. Abi1fl/fl mice (Floxed alleles for Abi1) were kindly provided by Dr. Leszek Kotula (SUNY Upstate Medical University). All experiments were performed with 7-12-week-old sex- and age-matched mice that were maintained on a 12-hours light-dark cycle, with food and water available ad libitum. All animals were bred separately and housed in specific pathogen-free barrier rooms kept at 22-26 °C and 45-55% humidity.
Wild animals	This study did not involve the use of any wild animals.
Reporting on sex	Both male and female mice were used in this study.
Field-collected samples	No field samples were collected for this study.
Ethics oversight	All animal care and experiments were conducted in compliance with the Association for Assessment and Accreditation of Laboratory Animal Care International regulations. All experimental protocols were approved by the Harvard Medical School Institutional Animal Care and Use Committee and were in compliance with all federal, state and local laws.

Note that full information on the approval of the study protocol must also be provided in the manuscript.

Plants

Seed stocks	No plants were used in this study.
Novel plant genotypes	No plants were used in this study.
Authentication	No plants were used in this study.
