# Supplementary material for: A pore-forming toxin initiates ABI1 complex switching to promote bacterial cell-to-cell spread
Source: Nat Commun. 2026 Apr 13;17:5129. doi: 10.1038/s41467-026-71510-z (PMC13247217; doi:10.1038/s41467-026-71510-z)
Supplement: Supplementary file 13 — Transparent Peer Review file [file 41467_2026_71510_MOESM13_ESM.pdf]

nature portfolio

Peer Review File

A pore-forming toxin initiates ABI1 complex switching to promote bacterial cell-to-cell spread

Corresponding Author: Professor Darren Higgins

This file contains all reviewer reports in order by version, followed by all author rebuttals in order by version.

Version 0:

Reviewer comments:

Reviewer #1

(Remarks to the Author)
The manuscript "A pore-forming toxin initiates ABI1 complex switching to promote bacterial cell-to-cell spread" by Sun et al. starts from in vivo models and then identify the molecular mechanisms behind the initial phenotype. The authors present a new cellular pathway targeted by Listeria monocytogenes to allow its cell-to-cell spread, going from the bacterial component involved to the full cellular cascade that is triggered.

The manuscript by Sun et al. is very well written and clear. The results and interpretations progress in a clear and logical manner, with strong results and well measured conclusions. The quantity and quality of their results are impressive. Understanding how the pathogens spread from cell to cell is a keystone in the fight against them and this study is undisputedly bringing a breakthrough for the field. I have no doubt that this manuscript is an important work for the host-pathogen interaction community and deserve to be published in Nature communication after having solved the few concerns mentioned below.

Major comments:
1- For each foci pictures (Figure 1f, 2d, 2f, 6k, Ext2a, Ext3g, Ext3l, Ext3q, Ext3v, Ext4g): Could the authors improve the GFP signal to allow to clearly identify the bacteria inside the foci because it is difficult to see them at the moment.
2- Line 152/153: The authors claim "our findings revealed no significant difference in plaque numbers, suggesting that ABI1 may not be directly involved in bacterial uptake". Did they authors clearing quantify the numbers of plaque that they obtained? I agreed that the pictures tend to show similar numbers of plaques but it is also difficult to evaluate correctly the number with the difference in size. Moreover, in their in vivo models, the authors clearly identified a reduced number of foci per square millimetre in their ABI1 KO mice showing that, in contrary, ABI1 seems to induce a bacterial uptake defect but they do not discuss about it. Please, could the authors explain this discrepancy between their in vivo and in cellulo models.
3- Figure 2 f,g,h: At which time point post-infection the authors analysed the foci size in the BMDM? In this cell type, the authors identified an important spreading defect, but the intracellular growth is affected only at 16-18hpi. If the authors succeeded to identity the spreading defect at 6hpi, the same than for the other cell lines, then, it does not seem logical to have a nice defect in foci size but the same intracellular growth for still around 10 hours. Could the authors comment on that please.
4- When the authors use microscopy to look at the actin tail and protrusions, the data are not as strong as the rest of the manuscript as it is representative pictures without quantification (Figure 3a, 3b, 3c, 5c, Ext6a-l, Ext9e-f).
Did the authors quantify the presence of ABI1 in the comet tails vs the protrusions? The percentage of protrusions with ABI1? Moreover, did the authors notice different localizations of ABI1 in the protrusion? Looking at their pictures, with the antibodies, ezrin and then ABI1 seem to be recruited at the bacteria, in the contrary to the cell lines expressing the markers where we see ABI1 near of the bacterial pole and then ezrin.
5- Figure 6k, i: The authors found that adding calpeptin to ABI1 KO cells do not change the spreading defect, but they never demonstrated that in absence of ABI1, SPTAN was cleaved. In AB1 KO cells, do you still have the SPTAN cleavage?

Minor comment:
1- Figure 1b: Did the authors monitored the weight of the mice during the 10 days of survival monitoring? It could also be informative about the level of sickness if the authors have these data and add them to the manuscript.
2- Line 120: the authors mentioned a very interesting hypothesis to explain the bacterial burden observed in the brain of the MKO mice despite a non-efficient KO in this organ: "ABI1 may be specifically depleted in brain endothelial cells". By

curiosity, I was wondering if the authors ever tried to validate this hypothesis by any technics.

Reviewer #2

(Remarks to the Author)
Review report for the manuscript NCOMMS-25-89727-T by Sun He at al.

Reviewer summary of key results
The authors investigate how Listeria monocytogenes coordinates host and bacterial factors to drive formation of actin-rich protrusions essential for cell-to-cell spread. In this manuscript they present a comprehensive and mechanistically elegant study where they identify the host protein ABI1 as a critical contributor to efficient protrusion formation and bacteria dissemination. First, using conditional Abi1 knockout mice, they show that loss of ABI1 markedly reduces susceptibility to L. monocytogenes infection. Mechanistically, they demonstrate that LLO-mediated Ca^{2+} influx activates calpain, leading to spectrin cleavage and release of ABI1 from the cortical cytoskeleton. Free ABI1 is then recruited to protrusions, where it associates with EPS8, switching EPS8 activity toward actin barbed-end capping to enhance local actin recycling and protrusion elongation. The study integrates in vivo mouse infections, multiple cellular infection models, advanced live-cell imaging, and quantitative proteomics to build a compelling mechanistic model. Overall, the authors describe a previously unrecognized mechanism by which a bacterial toxin promotes spatially restricted host cytoskeletal remodeling to enhance cell-to-cell spread.

Significance and Impact
This is a strong, well-written, and conceptually novel study with broad implications for host–pathogen interactions and actin-based motility. The findings are likely to be of substantial interest to both the microbiology and cytoskeleton research communities. This work makes several important conceptual advances: it redefines pore-forming toxins as spatial signaling modulators rather than solely cytolytic virulence determinants, it introduces the concept of pathogen-driven complex switching of ABI1 as a regulator of host cytoskeleton, and it establishes a mechanistic link between membrane damage, calpain activation, spectrin remodeling and actin dynamics during bacterial dissemination.

Methods and Conclusions
This study employs a comprehensive and technically rigorous experimental strategy that integrates conditional mouse genetics, quantitative proteomics, advanced live-cell imaging, and multiple in vitro and in vivo infection models. The use of two independent conditional Abi1 knockout mouse lines, coupled with infection using multiple bacterial strains and both intravenous and oral routes, provides strong physiological relevance and robust validation of the in vivo findings. The in vivo data are of particularly high quality and convincingly demonstrate a critical role for ABI1 in bacterial virulence and cell-to-cell spread. In addition, the mEOS3.2-based photo-convertible actin recycling assay represents a technically sophisticated and innovative approach, allowing for direct, quantitative measurement of local actin dynamics within protrusions. Importantly, the conclusions are well supported by the breadth of experimental approaches and the consistency of the data across systems.

Minor concerns
The reviewer identifies only minor points that the authors may consider addressing to further strengthen and clarify the manuscript. The comments below are intended to be addressed primarily through discussion and clarification rather than additional experimentation.

Infection foci spreading area quantification
It is not detailed in the Figure 1g legend or in the methods section how the spreading area of infection foci was determined, nor how the normalization was performed. Was the foci delimited manually or segmented using any software?

ABI1 KO impairs protrusion dynamics
The quantification (in percentage) of photo-converted mEOS3.2-Actin that migrates to the bacterial pole in relation to the total photo-converted mEOS3.2-Actin is approximately 20% at time 0 (before photo-conversion; Figure 4i). However, the images show that virtually no photoconverted signal is present at time 0 (Figure 4g, h). Is this the basal photoconversion level or does this value correspond to 1.2 sec (the first timepoint measured)?. Also, the statistical analysis of this experiment is not described in Figure 4 legend.

ABI1 interaction with EPS8 and EPS8 activity switching in Lm-containing protrusions
While the data strongly implicate the ABI1–EPS8 interaction in promoting cell-to-cell spread, the current experiments do not fully resolve whether ABI1 and EPS8 act strictly within the same linear pathway or retain partially independent contributions. The authors are encouraged to explicitly discuss this point, including whether a combined loss of ABI1 and EPS8 would be expected to yield additive or epistatic effects. In addition, because differences in protein expression levels can significantly influence the interpretation of the EPS8 mutant overexpression experiments (Figure 5f,g), the authors should clarify whether the wild-type and mutant EPS8 constructs are expressed at comparable levels and discuss how potential expression differences might affect the conclusions.

Calcium influx–mediated ABI1 redistribution
The data support a role for calcium signaling in regulating ABI1 redistribution; however, the authors may wish to slightly

temper the interpretation that LLO-induced calcium influx alone is sufficient to account for the observed ABI1 dynamics. Ionomycin elevates cytosolic calcium through both extracellular influx and release from intracellular stores (e.g., the endoplasmic reticulum), whereas LLO primarily triggers calcium entry from the extracellular space. It would therefore be helpful for the authors to briefly discuss this distinction and consider whether additional LLO-triggered, calcium-independent signaling pathways might also contribute to ABI1 redistribution.

Cytoplasmic activity of LLO
The authors propose that LLO may act locally from within the host cytoplasm to induce pore formation at the plasma membrane. Given that several host- and pathogen-mediated mechanisms are known to limit intracellular LLO activity—most notably the PEST-dependent degradation pathway that restricts LLO stability in the cytosol—it would be helpful for the authors to discuss how these regulatory mechanisms may intersect with their model. Specifically, a brief discussion of how localized LLO activity might be achieved despite these well-established inactivation processes would further strengthen the interpretation of the data.

ABI1 interactome
Could the authors clarify the rationale for the mass-spectrometry analyses performed? Why not compare HA-ABI1 + Lm / HA-ABI1 or HA-ABI1 + LmΔactA / HA-ABI1?

Other minor points
Line 467: “constitutive” should read “consecutive”
Figure 1a, Extended Figure 1g: “adminstration” should read “administration”
Figure 6a: the distribution of ABI1, EPS8 and Lm is difficult to observe. Could the authors provide larger images and separate the different color channels?
In the plaque formation assay, did the authors overlay the cells with a semi-solid medium (e.g. agarose or avicel?)
Line 1051: Please detail the microscope used for imaging
Why add b-mercaptoethanol to BMDM cultures?
Many reagents mentioned in the materials and methods section are missing the brand and reference (e.g. antibiotics)

Reviewer #3

(Remarks to the Author)
Summary
Based on a previous screen that identified ABI1 as a host factor required for Listeria monocytogenes infection, the authors investigated the role of ABI1 in mice and tissue culture cells. Using conditional knockout mice, they found that ABI1 played an important role in virulence and organ colonization in vivo. Furthermore, ABI1 contributed to cell-to-cell spread between various cell types in vivo and in vitro. In cultured cells, ABI1 localizesd to plasma membrane protrusions and played a role in protrusion elongation. ABI1 also interacted with the actin-binding protein EPS8, and EPS8 also contributed to cell-to-cell spread. The role of calcium influx and LLO activity was also investigated, and LLO was found to promote calcium influx, cleavage of spectrin, and a switch in ABI1 binding from ABI1-spectrin towards ABI1-EPS8 complexes.

Overall evaluation
The study presents a large amount of data and for the most part is very thorough. In general, the conclusions are well supported by the data. Overall, the study will make a valuable contribution to the literature. That said, there are a few practices related to rigor and reproducibility that need more attention to detail, for example listing the number of independent replicates, number of animals examined, and specific p values rather than ranges. Sources for key reagents like antibodies should also be listed in the methods section. Western blots should be quantified when possible. Moreover, the role of ABI1 in the WAVE complex should be more thoroughly considered and discussed.

Specific suggestions
1. Introduction and Results, section on ABI1 complex switching – a major fraction of ABI1 in cells is likely associated with the WAVE complex, yet this is not really mentioned until the discussion section. It is relevant to the idea of ABI1 complex switching and should be discussed earlier.
2. Fig. 1g, i: The legend says N=20 examples. How many mice were included in this analysis? How many independent replicates were carried out?
3. Fig. 3a, b, top rows: The images of whole cells are very small and it’s hard to see much detail. Moreover, information should be provided for the sources and specificity (whole images of Westerns) for key antibodies.
4. Videos 1 and 3: It’s very hard to see ABI1 in protrusions in these videos. The signal to noise is not great and the videos are rather small. Magnified images might help.
5. Fig. 4f-h: It seems there are a number of parameters that could be quantified in addition to “recycling,” including depolymerization and retrograde flux rates. The authors should consider doing additional analyses, which might add to their conclusions regarding actin dynamics in protrusions.
6. Fig. 6a and similar TIRF images: These images are very small and hard to see the data. Bigger images would be helpful.

Reviewer #4

(Remarks to the Author)
I co-reviewed this manuscript with one of the reviewers who provided the listed reports. This is part of the Nature Communications initiative to facilitate training in peer review and to provide appropriate recognition for Early Career Researchers who co-review manuscripts.

Open Access This Peer Review File is licensed under a Creative Commons Attribution 4.0 International License, which permits use, sharing, adaptation, distribution and reproduction in any medium or format, as long as you give appropriate credit to the original author(s) and the source, provide a link to the Creative Commons license, and indicate if changes were made.
In cases where reviewers are anonymous, credit should be given to 'Anonymous Referee' and the source.
The images or other third party material in this Peer Review File are included in the article's Creative Commons license, unless indicated otherwise in a credit line to the material. If material is not included in the article's Creative Commons license and your intended use is not permitted by statutory regulation or exceeds the permitted use, you will need to obtain permission directly from the copyright holder.
To view a copy of this license, visit https://creativecommons.org/licenses/by/4.0/

Response to Reviewers' comments

We appreciate the time and constructive suggestions provided by the editorial team and reviewers, which have greatly helped us improve the manuscript. Reviewer comments are shown in black, and our responses are provided in dark red.

REVIEWER COMMENTS

Reviewer #1 (Remarks to the Author):

The manuscript "A pore-forming toxin initiates ABI1 complex switching to promote bacterial cell-to-cell spread" by Sun et al. starts from in vivo models and then identify the molecular mechanisms behind the initial phenotype. The authors present a new cellular pathway targeted by Listeria monocytogenes to allow its cell-to-cell spread, going from the bacterial component involved to the full cellular cascade that is triggered.
The manuscript by Sun et al. is very well written and clear. The results and interpretations progress in a clear and logical manner, with strong results and well measured conclusions. The quantity and quality of their results are impressive.
Understanding how the pathogens spread from cell to cell is a keystone in the fight against them and this study is undisputedly bringing a breakthrough for the field. I have no doubt that this manuscript is an important work for the host-pathogen interaction community and deserve to be published in Nature communication after having solved the few concerns mentioned below.

Major comments:
1- For each foci pictures (Figure 1f, 2d, 2f, 6k, Ext2a, Ext3g, Ext3l, Ext3q, Ext3v, Ext4g): Could the authors improve the GFP signal to allow to clearly identify the bacteria inside the foci because it is difficult to see them at the moment.

We thank the reviewer for raising this point. The reduced visibility of the GFP signal in the originally submitted figures resulted from file compression during the initial submission process, which affected image resolution. In the original, uncompressed microscopy images, GFP-labeled bacteria within foci are clearly discernible. In the revised manuscript, we have provided the uncompressed higher-resolution images to ensure that the GFP signal and bacterial localization within foci are clearly visible.

2- Line 152/153: The authors claim "our findings revealed no significant difference in plaque numbers, suggesting that ABI1 may not be directly involved in bacterial uptake". Did they authors clearing quantify the numbers of plaque that they obtained? I agreed that the pictures tend to show similar numbers of plaques but it is also difficult to evaluate correctly the number with the difference in size. Moreover, in their in vivo models, the authors clearly identified a reduced number of foci per square millimetre in their ABI1 KO mice showing that, in contrary, ABI1 seems to induce a bacterial uptake defect but they do not discuss about it. Please, could the authors explain this discrepancy between their in vivo and in cellulo models.

We thank the reviewer for this important and thoughtful comment. In response, we have now quantified plaque numbers across all cell lines tested and included these data in the revised manuscript. The corresponding analyses are shown in Supplementary Fig. 3d, f, k, p, and u.

Consistent with our original statement, we observe no significant defect in bacterial uptake in *ABI1* knockout (KO) HeLa cells when assessed using the *in vitro* plaque formation assay (Supplementary Fig. 3d). Notably, however, we do detect a significant reduction in plaque numbers in *ABI1* KO HepG2 cells (Supplementary Fig. 3k), derived from a hepatocellular carcinoma, whereas no such reduction is observed in the other cell lines tested. These results indicate that the requirement for ABI1 in bacterial uptake may be cell-type dependent.

With respect to the apparent discrepancy between the *in vitro* plaque assay results and the reduced number of liver infection foci observed in *Abi1* KO mice *in vivo*, we believe this can be explained by two non-mutually exclusive factors. First, cell-type differences likely contribute to this phenotype. The reduced plaque numbers observed specifically in *ABI1* KO HepG2 cells (a liver hepatocyte cell line model) provide a plausible explanation for the decreased number of infection foci observed in liver tissue *in vivo*.

Second, fundamental differences between *in vitro* cell-based infection models and *in vivo* infection dynamics are also likely to play an important role. Following intravenous *Listeria monocytogenes* (*Lm*) infection, Kupffer cells (liver-resident macrophages) are the primary cell type responsible for early active bacterial uptake and hepatocyte infection can occur through heterologous cell-to-cell spread, thereby contributing to the establishment of infection foci[1, 2]. Thus, the reduced number of liver infection foci observed in *Abi1* KO mice likely reflects a combination of impaired hepatocyte uptake and reduced cell-to-cell spread from infected macrophages to hepatocytes. Consistent with this model, we propose that a major function of ABI1 during *Lm* infection is to promote efficient bacterial cell-to-cell spread. We have revised the manuscript to clarify these distinctions. (Lines 180-184)

3- Figure 2 f,g,h: At which time point post-infection the authors analysed the foci size in the BMDM? In this cell type, the authors identified an important spreading defect, but the intracellular growth is affected only at 16-18hpi. If the authors succeeded to identity the spreading defect at 6hpi, the same than for the other cell lines, then, it does not seem logical to have a nice defect in foci size but the same intracellular growth for still around 10 hours. Could the authors comment on that please.

We thank the reviewer for this thoughtful comment. The foci size analysis shown in Fig. 2f,g was performed at 18 hour-post-infection (hpi) in BMDM. (Line 197)

In contrast to epithelial cell lines, *Lm* spreads substantially more slowly and less efficiently in primary macrophages. This behavior is well documented and reflects several macrophage-specific features, including rapid phagosomal acidification, accumulation of antimicrobial effectors, efficient sensing of membrane damage, and activation of autophagic and inflammatory responses that limit bacterial survival and cytosolic access[3]. As a consequence, productive cell-to-cell spread events in BMDMs occur predominantly at later time points compared with epithelial cells.

Accordingly, assessing foci size at 18 hpi is necessary to reliably capture spreading phenotypes in this cell type. This timing also explains why a clear defect in foci size is observed at 18 hpi, whereas

intracellular bacterial growth curves appear largely similar between genotypes at earlier time points. We have now clarified the timing of the foci size analysis and the macrophage-specific infection kinetics in the revised manuscript. (Lines 193-196)

4- When the authors use microscopy to look at the actin tail and protrusions, the data are not as strong as the rest of the manuscript as it is representative pictures without quantification (Figure 3a, 3b, 3c, 5c, Ext6a-l, Ext9e-f).
Did the authors quantify the presence of ABI1 in the comet tails vs the protrusions? The percentage of protrusions with ABI1? Moreover, did the authors notice different localizations of ABI1 in the protrusion? Looking at their pictures, with the antibodies, ezrin and then ABI1 seem to be recruited at the bacteria, in the contrary to the cell lines expressing the markers where we see ABI1 near of the bacterial pole and then ezrin.

We thank the reviewer for this important comment. In the revised manuscript, we have now quantified ABI1 recruitment to *Lm*-induced actin structures, including actin comet tails and protrusions. These quantifications have been incorporated into the revised figures (Fig. 3b, 3d, 5d, and Supplementary Fig. 6m, 9f, 9h). Together, these analyses further strengthen our conclusion that EPS8/L2 is recruited to actin clouds and comet tails, whereas ABI1 is selectively recruited at *Lm*-containing protrusions during protrusion formation.

With respect to the reviewer's question regarding differential ABI1 localization within protrusions between antibody-based immunofluorescence and cell lines expressing fluorescent markers, we believe these differences primarily reflect methodological rather than biological effects. Immunofluorescence relies on antibody specificity and epitope accessibility, which can be limited in dense, actin-rich protrusions, thereby reducing spatial resolution and potentially affecting the apparent distribution or order of protein recruitment. To overcome these limitations, we employed a CRISPR-mediated endogenous tagging strategy combined with live-cell imaging, which enables real-time visualization of ABI1 and EZRIN dynamics with higher spatial and temporal resolution and avoids potential artifacts associated with antibody penetration or epitope masking (Fig. 3c).

Importantly, results obtained using both approaches are consistent in demonstrating a close association of ABI1 with *Lm*-containing protrusions. When considered together, the immunofluorescence and live-cell imaging data support a model in which ABI1 is preferentially recruited to *Lm*-induced protrusions, whereas EZRIN accumulates along the protrusion membrane as a marker of protrusion structure. We have clarified this interpretation and added the corresponding quantifications in the revised manuscript. (Lines 225-228)

5- Figure 6k, i: The authors found that adding calpeptin to ABI1 KO cells do not change the spreading defect, but they never demonstrated that in absence of ABI1, SPTAN was cleaved. In AB1 KO cells, do you still have the SPTAN cleavage?
the SPTAN1 cleavage is independent of ABI1.

We thank the reviewer for raising this important point. We have performed experiments and determined that SPTAN1 cleavage occurs in the absence of ABI1. We have added the new data in the revised manuscript. As shown in Supplementary Fig. 10i, SPTAN1 cleavage is readily detected in

ABI1 KO cells upon *Lm* infection, at levels comparable to those observed in WT cells. These data demonstrate that SPTAN1 cleavage is independent of ABI1.

Therefore, the inability of calpeptin treatment to rescue the spreading defect in *ABI1* KO cells does not reflect a failure of SPTAN1 cleavage but rather indicates that ABI1 functions downstream of spectrin cleavage in promoting bacterial cell-to-cell spread. We have clarified this important point in the revised manuscript. (Lines 404-406)

Minor comment:

1- Figure 1b: Did the authors monitored the weight of the mice during the 10 days of survival monitoring? It could also be informative about the level of sickness if the authors have these data and add them to the manuscript.

We thank the reviewer for this helpful suggestion. Body weight was not monitored during the LD_{50} survival experiments and therefore these data are not available. Given time and resource constraints, we did not repeat these experiments to include body weight measurements.

While body weight can provide additional information regarding disease severity, we do not anticipate that its inclusion would alter the overall conclusions of the survival analysis. In this study, disease severity was assessed using multiple complementary readouts, including bacterial burdens in multiple organs, survival over the 10-day monitoring period, and deduced LD_{50} calculations, which together provide a robust assessment of infection outcome.

2- Line 120: the authors mentioned a very interesting hypothesis to explain the bacterial burden observed in the brain of the MKO mice despite a non-efficient KO in this organ: “ABI1 may be specifically depleted in brain endothelial cells”. By curiosity, I was wondering if the authors ever tried to validate this hypothesis by any technics.

We thank the reviewer for the interest in this hypothesis. Preferential depletion of ABI1 in brain endothelial cells has been reported previously[4]. In the present study, we did not directly test this hypothesis, as doing so would require cell-type-specific approaches that are beyond the scope of the current work. We have clarified this point in the revised manuscript and note that future studies using brain endothelial cell-specific *ABI1* knockout models will be of great interest and hold strong promise for directly addressing this interesting question. (Lines 122-127)

Reviewer #2 (Remarks to the Author):

Review report for the manuscript NCOMMS-25-89727-T by Sun He at al.

Reviewer summary of key results

The authors investigate how Listeria monocytogenes coordinates host and bacterial factors to drive formation of actin-rich protrusions essential for cell-to-cell spread. In this manuscript they present a comprehensive and mechanistically elegant study where they identify the host protein ABI1 as a

critical contributor to efficient protrusion formation and bacteria dissemination. First, using conditional Abi1 knockout mice, they show that loss of ABI1 markedly reduces susceptibility to L. monocytogenes infection. Mechanistically, they demonstrate that LLO-mediated Ca^{2+} influx activates calpain, leading to spectrin cleavage and release of ABI1 from the cortical cytoskeleton. Free ABI1 is then recruited to protrusions, where it associates with EPS8, switching EPS8 activity toward actin barbed-end capping to enhance local actin recycling and protrusion elongation. The study integrates in vivo mouse infections, multiple cellular infection models, advanced live-cell imaging, and quantitative proteomics to build a compelling mechanistic model. Overall, the authors describe a previously unrecognized mechanism by which a bacterial toxin promotes spatially restricted host cytoskeletal remodeling to enhance cell-to-cell spread.

Significance and Impact

This is a strong, well-written, and conceptually novel study with broad implications for host–pathogen interactions and actin-based motility. The findings are likely to be of substantial interest to both the microbiology and cytoskeleton research communities. This work makes several important conceptual advances: it redefines pore-forming toxins as spatial signaling modulators rather than solely cytolytic virulence determinants, it introduces the concept of pathogen-driven complex switching of ABI1 as a regulator of host cytoskeleton, and it establishes a mechanistic link between membrane damage, calpain activation, spectrin remodeling and actin dynamics during bacterial dissemination.

Methods and Conclusions

This study employs a comprehensive and technically rigorous experimental strategy that integrates conditional mouse genetics, quantitative proteomics, advanced live-cell imaging, and multiple in vitro and in vivo infection models. The use of two independent conditional Abi1 knockout mouse lines, coupled with infection using multiple bacterial strains and both intravenous and oral routes, provides strong physiological relevance and robust validation of the in vivo findings. The in vivo data are of particularly high quality and convincingly demonstrate a critical role for ABI1 in bacterial virulence and cell-to-cell spread. In addition, the mEOS3.2-based photo-convertible actin recycling assay represents a technically sophisticated and innovative approach, allowing for direct, quantitative measurement of local actin dynamics within protrusions. Importantly, the conclusions are well supported by the breadth of experimental approaches and the consistency of the data across systems.

Minor concerns

The reviewer identifies only minor points that the authors may consider addressing to further strengthen and clarify the manuscript. The comments below are intended to be addressed primarily through discussion and clarification rather than additional experimentation.

Infection foci spreading area quantification

It is not detailed in the Figure 1g legend or in the methods section how the spreading area of infection foci was determined, nor how the normalization was performed. Was the foci delimited manually or segmented using any software?

We apologize for the lack of detail in the original manuscript. The spreading area of liver infection foci was quantified by manually delineating individual foci in Fiji based on the fluorescent signal, followed by measurement of the selected area. Manual delineation is commonly used for this type of analysis, as infection foci often display heterogeneous shapes and variable fluorescence intensities that are difficult to capture reliably using automated segmentation approaches. Moreover, in our experiments, differences in spreading area between conditions were robust and readily apparent, rather than subtle or marginal. To minimize potential bias, all images were analyzed using identical criteria in Fiji, and quantification was performed in a consistent manner across all conditions. For normalization, the mean spreading area of WT(E) infection foci was calculated and used as the reference value, with values from all other conditions expressed relative to this mean. We have now clarified these procedures in both the Fig. 1g legend and the Methods section. (Lines 1172-1174)

ABI1 KO impairs protrusion dynamics
The quantification (in percentage) of photo-converted mEOS3.2-Actin that migrates to the bacterial pole in relation to the total photo-converted mEOS3.2-Actin is approximately 20% at time 0 (before photo-conversion; Figure 4i). However, the images show that virtually no photoconverted signal is present at time 0 (Figure 4g, h). Is this the basal photoconversion level or does this value correspond to 1.2 sec (the first timepoint measured)?. Also, the statistical analysis of this experiment is not described in Figure 4 legend.

We thank the reviewer for this careful observation. The quantification of photo-converted mEOS3.2-Actin was initiated from the first frame acquired immediately after photo-conversion. In the quantification, this frame is defined as time 0. Images were acquired at a temporal resolution of 0.24 s, whereas representative images in Fig. 4g,h were displayed at 1.2 s intervals for clarity and presentation purposes. As a result, the "time 0" point used for quantification is not shown in the representative images, which explains the discrepancy between the quantified values and the displayed panels. The ~20% value at time 0 therefore reflects the initial redistribution of photo-converted actin immediately following photo-conversion, rather than basal photoconversion prior to illumination. Mixed-effects modeling was used to account for repeated measurements over time within *Lm*-containing protrusions, as well as variability across individual cells and independent experiments, when comparing local actin recycling across different genetic backgrounds. We have now added a detailed description of the analysis used for this experiment in the revised figure legend for Fig 4i. (Lines 618-619)

ABI1 interaction with EPS8 and EPS8 activity switching in Lm-containing protrusions
While the data strongly implicate the ABI1–EPS8 interaction in promoting cell-to-cell spread, the current experiments do not fully resolve whether ABI1 and EPS8 act strictly within the same linear pathway or retain partially independent contributions. The authors are encouraged to explicitly discuss this point, including whether a combined loss of ABI1 and EPS8 would be expected to yield additive or epistatic effects. In addition, because differences in protein expression levels can significantly influence the interpretation of the EPS8 mutant overexpression experiments (Figure 5f,g), the authors should clarify whether the wild-type and mutant EPS8 constructs are expressed at comparable levels and discuss how potential expression differences might affect the conclusions.

We thank the reviewer for this insightful comment. Our data strongly support a functional interaction between ABI1 and EPS8 in promoting *Lm* cell-to-cell spread; however, we agree that the current

experiments do not definitively resolve whether ABI1 and EPS8 act strictly within a single linear pathway or retain partially independent contributions. At present, our data are most consistent with a model in which ABI1 functions upstream of EPS8 to regulate *Lm*-containing protrusion dynamics, while not excluding the possibility that each protein may also exert additional, context-dependent roles. We have now explicitly discussed this point in the revised manuscript and noted that genetic epistasis experiments, such as combined loss of ABI1 and EPS8, would be an important future direction to distinguish between linear and partially independent models (Lines 429-432). Based on our current results, we hypothesize that combined loss of ABI1 and EPS8 may result in a phenotype similar to that observed upon loss of EPS8 family function.

With respect to the EPS8 mutant overexpression experiments (now Fig. 5g,h), we verified the expression levels of wild-type EPS8 and the mutant EPS8 constructs by immunoblotting and confirmed that they are expressed at comparable levels (Reviewer Fig. 1), assessed in parallel with the plaque formation assays. Due to space limitations, these data were not included in the revised manuscript. Nevertheless, this validation supports the conclusion that the observed functional differences reflect altered EPS8 activity rather than differences in protein abundance.

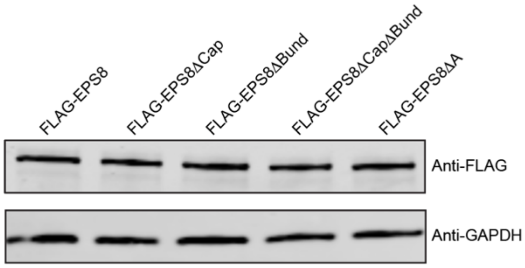

Reviewer Fig. 1 Western blot analysis of EPS8 variant expression in HeLa cells. Expression levels of EPS8 mutants were detected using an anti-FLAG antibody with GAPDH as a loading control.

Calcium influx–mediated ABI1 redistribution

The data support a role for calcium signaling in regulating ABI1 redistribution; however, the authors may wish to slightly temper the interpretation that LLO-induced calcium influx alone is sufficient to account for the observed ABI1 dynamics. Ionomycin elevates cytosolic calcium through both extracellular influx and release from intracellular stores (e.g., the endoplasmic reticulum), whereas LLO primarily triggers calcium entry from the extracellular space. It would therefore be helpful for the authors to briefly discuss this distinction and consider whether additional LLO-triggered, calcium-independent signaling pathways might also contribute to ABI1 redistribution.

We thank the reviewer for this thoughtful and insightful comment. We agree that ionomycin and LLO elevate cytosolic Ca^{2+} through distinct mechanisms and that this distinction is important for interpretation. As the reviewer pointed out, ionomycin increases cytosolic Ca^{2+} by promoting both extracellular Ca^{2+} influx and release from intracellular stores, such as the endoplasmic reticulum, whereas LLO primarily induces Ca^{2+} entry from the extracellular space through pore formation at the plasma membrane. In our study, ionomycin was used as a tool to test whether elevated cytosolic Ca^{2+} is sufficient to drive ABI1 redistribution, rather than to precisely mimic the mechanism of LLO action. To more directly assess the contribution of extracellular Ca^{2+} influx, we performed EGTA chelation experiments. EGTA selectively chelates extracellular Ca^{2+} and does not block Ca^{2+} release from

intracellular stores. Under these conditions, we observed that EGTA treatment effectively blocked ionomycin-induced spectrin (SPTAN1) cleavage, indicating that extracellular Ca^{2+} influx is required for SPTAN1 cleavage (Supplementary Fig. 10b).

Together, these data support a model in which LLO-induced extracellular Ca^{2+} influx is a key trigger for ABI1 redistribution during infection. At the same time, we agree that Ca^{2+} influx alone may not fully account for all aspects of ABI1 dynamics, and that additional LLO-triggered, Ca^{2+}-independent signaling pathways may also contribute. We have now tempered our interpretation accordingly and added a discussion of these points in the revised manuscript. (Lines 433-436)

Cytoplasmic activity of LLO

The authors propose that LLO may act locally from within the host cytoplasm to induce pore formation at the plasma membrane. Given that several host- and pathogen-mediated mechanisms are known to limit intracellular LLO activity—most notably the PEST-dependent degradation pathway that restricts LLO stability in the cytosol—it would be helpful for the authors to discuss how these regulatory mechanisms may intersect with their model. Specifically, a brief discussion of how localized LLO activity might be achieved despite these well-established inactivation processes would further strengthen the interpretation of the data.

We thank the reviewer for raising this important point. We agree that multiple host- and pathogen-mediated mechanisms limit the stability and activity of LLO within the host cytosol and are critical for preventing excessive cytotoxicity during infection.

Our model does not imply sustained or global LLO activity within the cytoplasm. Rather, we propose that LLO may act in a highly localized and transient manner, in close spatial proximity to the plasma membrane, before efficient cytosolic inactivation occurs. Such localized activity could allow LLO to induce restricted pore formation and Ca^{2+} influx sufficient to trigger downstream signaling events, including spectrin cleavage and ABI1 redistribution, without requiring prolonged cytosolic stability.

In addition, LLO activity is known to be tightly regulated by factors such as cholesterol-dependent membrane binding, pH sensitivity, and host-mediated clearance mechanisms, including endocytic pathways that limit toxin persistence at the plasma membrane. Together, these regulatory processes are likely to confine LLO function both spatially and temporally, thereby permitting transient, localized signaling at the plasma membrane while minimizing broader cytosolic toxicity.

We have now expanded the Discussion to explicitly address how established LLO regulatory and inactivation mechanisms are compatible with a model in which transient and spatially restricted LLO activity contributes to host signaling during infection. (Lines 439-450)

ABI1 interactome

Could the authors clarify the rationale for the mass-spectrometry analyses performed? Why not compare HA-ABI1 + Lm / HA-ABI1 or HA-ABI1 + LmΔactA / HA-ABI1?

We thank the reviewer for this question and apologize if the experimental rationale was not sufficiently clear. The goal of the mass spectrometry analysis was to identify ABI1 interaction partners that are

specifically associated with *Lm*-containing protrusion formation. To this end, we compared HA-ABI1 immunoprecipitates from cells infected with WT *Lm* versus *Lm∆actA*, which lack actin-based motility and therefore do not form protrusions, with both conditions normalized to HA-ABI1 expressed in uninfected cells.

This comparison, shown in Fig. 5a, directly corresponds to the conditions suggested by the reviewer and allows us to isolate possible protrusion-specific changes in ABI1-associated protein complexes that depend on cell-to-cell spread. We have revised the manuscript to clarify this experimental logic explicitly. (Lines 289-292)

Other minor points
Line 467: “constitutive” should read “consecutive”

We thank the reviewer for pointing out this error. The term “constitutive” has been corrected to “consecutive” in the revised manuscript. (Line 494)

Figure 1a, Extended Figure 1g: “adminstration” should read “administration”

We thank the reviewer for noting this typographical error. “Adminstration” has been corrected to “administration” in Fig. 1a and Supplementary Fig. 1g in the revised manuscript.

Figure 6a: the distribution of ABI1, EPS8 and Lm is difficult to observe. Could the authors provide larger images and separate the different color channels?

We thank the reviewer for this suggestion and agree that, at the scale presented, the distribution of ABI1, EPS8, and *Lm* can be difficult to resolve in Fig. 6a. We would like to clarify that the purpose of this panel is not to provide a detailed analysis of the relative subcellular localization of ABI1 and EPS8, but rather to serve as a control demonstrating that *Lm∆hly* can escape the vacuole, access the cytosol, and recruit EPS8, consistent with previous observations.

We have enlarged Fig 6a for clearer presentation. However, providing separate individual color channels for this panel would substantially increase the figure size and detract from the overall layout. Where detailed localization is central to our conclusions, higher-magnification images and single-channel views are provided in other panels and figures. We have clarified this point in the revised figure legend to better guide the reader. (Line 673)

In the plaque formation assay, did the authors overlay the cells with a semi-solid medium (e.g. agarose or avicel?)

In our plaque formation assays, cells were not overlaid with a semi-solid medium such as agarose or Avicel. Instead, plaques were formed under liquid culture conditions, and infected monolayers were subsequently fixed and stained with crystal violet to visualize areas of cell clearance resulting from *Lm* infection.

This approach allowed robust visualization and quantification of plaque formation while preserving cell viability and monolayer integrity under our experimental conditions. Similar crystal violet plaque assays have been used previously to assess plaque formation[5].

Line 1051: Please detail the microscope used for imaging

We thank the reviewer for pointing this out. We have now added detailed information about the microscope for these experiments to the Methods section. (Lines 1122-1125)

Why add b-mercaptoethanol to BMDM cultures?

β-mercaptoethanol is routinely added to primary macrophage and myeloid progenitor cultures to maintain a reduced extracellular environment. This reduces oxidative stress, improves cysteine availability, and supports redox-sensitive metabolic processes, thereby enhancing macrophage survival, proliferation, and differentiation.

Many reagents mentioned in the materials and methods section are missing the brand and reference (e.g. antibiotics)

We thank the reviewer for pointing this out. We have now added the manufacturer names and catalog numbers for all reagents mentioned in the Methods section, including antibiotics, to ensure clarity and reproducibility.

Reviewer #3 (Remarks to the Author):

Summary

Based on a previous screen that identified ABI1 as a host factor required for Listeria monocytogenes infection, the authors investigated the role of ABI1 in mice and tissue culture cells. Using conditional knockout mice, they found that ABI1 played an important role in virulence and organ colonization in vivo. Furthermore, ABI1 contributed to cell-to-cell spread between various cell types in vivo and in vitro. In cultured cells, ABI1 localizesd to plasma membrane protrusions and played a role in protrusion elongation. ABI1 also interacted with the actin-binding protein EPS8, and EPS8 also contributed to cell-to-cell spread. The role of calcium influx and LLO activity was also investigated, and LLO was found to promote calcium influx, cleavage of spectrin, and a switch in ABI1 binding from ABI1-spectrin towards ABI1-EPS8 complexes.

Overall evaluation

The study presents a large amount of data and for the most part is very thorough. In general, the conclusions are well supported by the data. Overall, the study will make a valuable contribution to the literature. That said, there are a few practices related to rigor and reproducibility that need more attention to detail, for example listing the number of independent replicates, number of animals examined, and specific p values rather than ranges. Sources for key reagents like antibodies should also be listed in the methods section. Western blots should be quantified when possible. Moreover,

the role of ABI1 in the WAVE complex should be more thoroughly considered and discussed.

We thank the reviewer for this overall positive and constructive assessment of our study. We agree that rigor and reproducibility are essential, and we have taken several steps to address the points raised.

Specifically, we have now provided the number of independent biological replicates and, where applicable, the number of animals analyzed for each experiment. Exact *P* values have been reported instead of ranges throughout the manuscript. In addition, the sources, manufacturers, and catalog numbers for key reagents, including antibodies, have been added to the Methods section. Where Western blot data are used to support quantitative interpretations, we have now included densitometric quantification and incorporated these data into the revised figures. Finally, we have added the role of ABI1 within the WAVE complex in the revised manuscript. (Lines 59-60)

Together, we believe these revisions strengthen the rigor, transparency, and interpretability of the study, and we thank the reviewer for these valuable suggestions.

Specific suggestions

1. Introduction and Results, section on ABI1 complex switching – a major fraction of ABI1 in cells is likely associated with the WAVE complex, yet this is not really mentioned until the discussion section. It is relevant to the idea of ABI1 complex switching and should be discussed earlier.

We thank the reviewer for this important suggestion. We agree that the association of ABI1 with the WAVE complex is highly relevant to the concept of ABI1 complex switching. We have therefore revised the manuscript to explicitly acknowledge that a substantial fraction of cellular ABI1 is associated with the WAVE complex and contributes to actin regulatory functions and added a reference citation. (Lines 59-60)

2. Fig. 1g, i: The legend says N=20 examples. How many mice were included in this analysis? How many independent replicates were carried out?

We thank the reviewer for this question and apologize for the lack of clarity. For both Fig. 1g and Fig. 1i, five mice per group were analyzed, which constitute the biological replicates. For Fig. 1g, N = 20 refers to 20 individual infection foci quantified from liver sections derived from these five mice. For Fig. 1i, N = 20 refers to 20 whole liver sections obtained from the same five mice. This also applies to the Supplementary Fig. 2b,d.

We have now clarified these details in the corresponding figure legends.

3. Fig. 3a, b, top rows: The images of whole cells are very small and it's hard to see much detail. Moreover, information should be provided for the sources and specificity (whole images of Westerns) for key antibodies.

We thank the reviewer for raising this point. We believe the image clarity issue is in part due to figure compression during the initial submission process. We have therefore enlarged these images and provided higher-resolution versions in the revised manuscript to improve clarity.

In addition, information regarding the sources of all antibodies used in this study has now been added to the Methods section and Table S2. Full, uncropped Western blot images corresponding to all figures are provided in the Source Data file. Furthermore, validation data for the antibodies used for immunofluorescence, including anti-ABI1 (Proteintech, Cat.# 27387-1-AP) and anti-EZRIN (Invitrogen, Cat.# 35-7300), are now included as Reviewer Fig. 2.

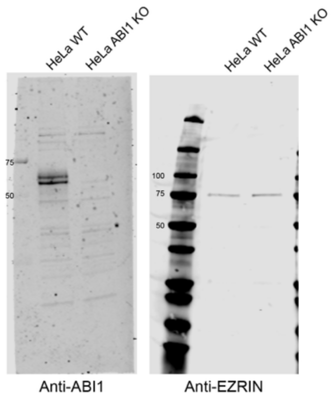

Reviewer Fig. 2 Uncropped full Western blot images for validation of the antibodies used for immunofluorescence, including anti-ABI1 (Proteintech, Cat.# 27387-1-AP) and anti-EZRIN (Invitrogen, Cat.# 35-7300)

4. Videos 1 and 3: It’s very hard to see ABI1 in protrusions in these videos. The signal to noise is not great and the videos are rather small. Magnified images might help.

We thank the reviewer for this comment. The original, uncompressed videos provide substantially improved signal quality. In the revised submission, we have therefore replaced Videos 1 and 3 with higher-resolution versions and additionally generated magnified views focusing on protrusion regions to better illustrate ABI1 dynamics. These changes improve the visibility of ABI1 within protrusions and facilitate clearer interpretation of the data.

5. Fig. 4f-h: It seems there are a number of parameters that could be quantified in addition to “recycling,” including depolymerization and retrograde flux rates. The authors should consider doing additional analyses, which might add to their conclusions regarding actin dynamics in protrusions

We thank the reviewer for this thoughtful suggestion. We agree that additional parameters of actin dynamics, such as depolymerization rates and retrograde flow, are important and could provide further mechanistic insight into protrusion behavior.

However, the primary goal of the analyses in Fig. 4f-h was to assess local actin recycling as a readout of productive actin-based protrusion dynamics, which is the parameter most directly linked to efficient cell-to-cell spread in this context. While additional quantitative metrics would undoubtedly be

informative, we believe that they would not alter the central conclusions of the present study regarding the role of ABI1 in regulating actin remodeling within protrusions.

Given the scope of the current work, we therefore focused on local actin recycling as a robust and biologically relevant measure. We view a more comprehensive quantitative dissection of actin dynamics as an important direction for future studies.

6. Fig. 6a and similar TIRF images: These images are very small and hard to see the data. Bigger images would be helpful.

We thank the reviewer for this comment. We believe this was largely due to figure compression during the initial submission process. In the revised manuscript, we have provided higher-resolution images and enlarged the images to improve clarity and facilitate visualization of the TIRF data.

Reviewer #4 (Remarks to the Author):

I co-reviewed this manuscript with one of the reviewers who provided the listed reports. This is part of the Nature Communications initiative to facilitate training in peer review and to provide appropriate recognition for Early Career Researchers who co-review manuscripts.

We would like to thank the reviewer for their careful, constructive, and insightful comments. Their feedback significantly improved the clarity, rigor, and presentation of our study, and we are grateful for the time and effort invested in this review.

References

1. Bleriot, C. *et al.* Liver-resident macrophage necroptosis orchestrates type 1 microbicidal inflammation and type-2-mediated tissue repair during bacterial infection. *Immunity* 42, 145-158 (2015).
2. Gregory, S.H., Barczynski, L.K. & Wing, E.J. Effector function of hepatocytes and Kupffer cells in the resolution of systemic bacterial infections. *J Leukoc Biol* 51, 421-424 (1992).
3. Pamer, E.G. Immune responses to *Listeria monocytogenes*. *Nat Rev Immunol* 4, 812-823 (2004).
4. Schneider, A., Zhang, Y., Guan, Y., Davis, L.S. & Breyer, M.D. Differential, inducible gene targeting in renal epithelia, vascular endothelium, and viscera of *Mx1Cre* mice. *Am J Physiol Renal Physiol* 284, F411-417 (2003).
5. Mendoza, E.J., Manguiat, K., Wood, H. & Drebot, M. Two Detailed Plaque Assay Protocols for the Quantification of Infectious SARS-CoV-2. *Curr Protoc Microbiol* 57, ecpmc105 (2020).
